# Synthesis of Glycopeptide Antibiotic Peptide Substrates Using Knorr‐Pyrazole Chemistry

**DOI:** 10.1002/cmdc.70487

**Published:** 2026-09-05

**Authors:** Jemma Gullick, W. Ramani H. Weerasinghe, Ralf B. Schittenhelm, Julien Tailhades, Max J. Cryle

**Affiliations:** ^1^ Department of Biochemistry and Molecular Biology The Monash Biomedicine Discovery Institute Monash University Clayton Victoria Australia; ^2^ ARC Centre of Excellence for Innovations in Peptide and Protein Science Clayton Victoria Australia; ^3^ The Monash Proteomics and Metabolomics Platform Monash University Clayton Victoria Australia

**Keywords:** antibiotic peptide, chemistry, glycopeptide antibiotic, peptide, peptide synthesis, pyrazole, teicoplanin, vancomycin

## Abstract

The glycopeptide antibiotics (GPAs) remain clinical antibiotics used against drug‐resistant Gram‐positive infections. The discovery of GPAs continues, with new type IIa and V GPAs, the kineomicins, the rimomycins, and corbomycin, being recently identified. Despite this larger repertoire of GPA scaffolds and crosslinking types, limitations remain with the in vitro exploration of the cytochrome P450 enzymes that install the essential crosslinks between the aromatic side chain residues of GPAs. While the chemoenzymatic synthesis of the more hydrophilic type I GPAs like vancomycin has aided our understanding of GPA crosslinking pathways, type II–V GPAs remain underexplored. This is due to the hydrophobic nature of these GPAs that makes access to peptidyl‐CoA substrates difficult and reduces in vitro crosslinking activity. Here, we explore the use of modified Knorr‐pyrazole chemistry to provide access to hydrophobic peptidyl‐CoA substrates of the type II/IIa GPAs, kineomicin and actinoidin, and the type IV GPA, teicoplanin. Yields of peptidyl‐CoAs were improved 3–7‐fold through careful tuning of reaction solvents and the arylthiol used for displacement of the pyrazole. The type II/IIa GPA scaffolds explored in this study displayed the highest in vitro OxyC activity seen to date, likely due to both improved synthesis and their structural properties.

## Graphical abstract

The glycopeptide antibiotics (GPAs) are important clinical antibiotics. While chemoenzymatic synthesis of more hydrophilic GPAs like vancomycin has aided our understanding of GPA crosslinking, hydrophobic GPAs remain underexplored. Here, we show that modified Knorr‐pyrazole chemistry improves access to hydrophobic peptidyl‐CoAs, with such substrates affording the highest in vitro OxyC activity seen to date.

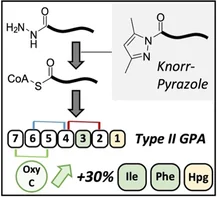

## 1 Introduction

**FIGURE 1:**
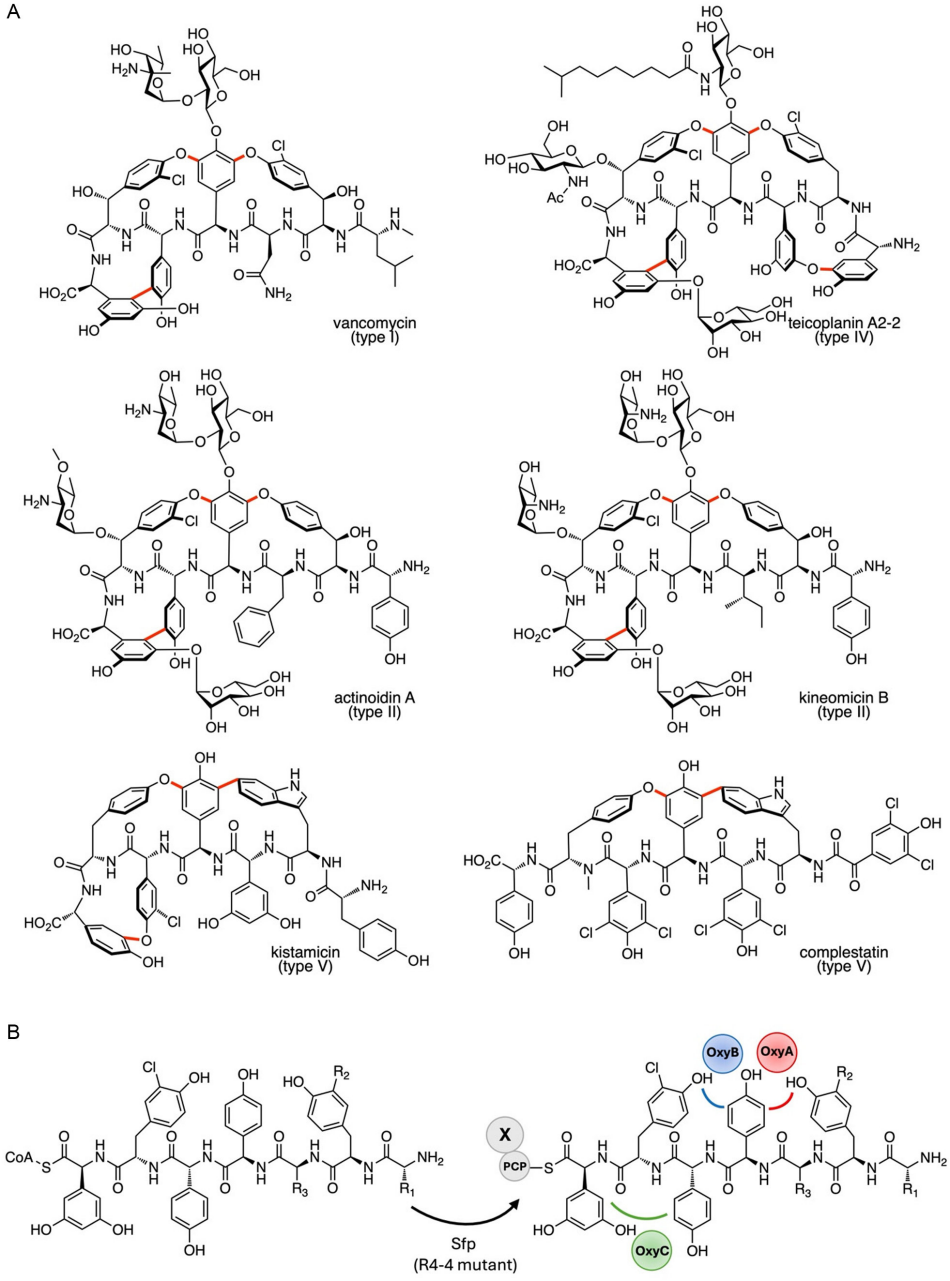
(A) Exemplars of the glycopeptide antibiotics, including vancomycin (type I GPA), teicoplanin A2‐2 (type IV GPA), actinoidin A (type II GPA), kineomicin B (type IIa GPA), kistamicin (type V GPA), and complestatin (type V GPA) with crosslinks installed by Oxy enzymes shown in red. (B) Schematic of peptidyl‐CoA loading onto PCP‐X didomain with Sfp (R4‐4 mutant) and sites of crosslinking by Oxy enzymes shown for a model type I/II GPA peptide.

The glycopeptide antibiotics (GPAs) are structurally complex nonribosomal peptide antibiotics used as last‐resort treatments against drug‐resistant Gram‐positive bacterial infections [1, 2, 3]. GPAs have remained in clinical use for many years thanks to their activity against methicillin‐resistant *Staphylococcus aureus*, *Streptococcus pneumoniae*, and *Clostridioides difficile* infections, although there are concerns of growing bacterial resistance toward GPAs [1, 2, 3]. Since the discovery of vancomycin in 1952, many structurally diverse GPAs have been reported, which span five unique subtypes of this class of antibiotic (Figure 1A) [2, 4]. While the GPAs are separated into different subtypes based on structure (also supported by evolutionary studies) [5, 6, 7], a conserved feature of all GPAs is extensive side‐chain crosslinking of aromatic residues [8, 9, 10]. Type I–IV GPAs target cell wall biosynthesis by binding to the d‐alanyl‐d‐alanine (d‐Ala‐d‐Ala) termini of the cell wall precursor Lipid II, thus blocking the enzymes involved in the formation of the bacterial cell wall. Side chain crosslinking is essential for the activity of Lipid II binding GPAs, with C‐*O*‐D, D‐*O*‐E, and A‐B rings found in all type I–IV GPAs [5, 6]. Type I and II GPAs share 3 crosslinks, with type I GPAs possessing aliphatic residues at position 1 and 3 of the peptide [5, 6]. Type II GPAs contain aromatic residues at P1 and P3, and the recently identified kineomicins exist as a hybrid of these two classes (termed type IIa) [11]. An additional crosslink, the F‐*O*‐G ring, is present between aromatic residues at P1 and P3 in the type III–IV GPAs, although this ring is not essential for antibiotic activity [5, 6]. The crosslinks found in GPAs are installed by cytochrome P450 enzymes (known as Oxys) [12] that are recruited to the precursor peptide while it is attached to the final module of the nonribosomal peptide synthetase (NRPS) machinery (Figure 1B) [13, 14, 15, 16]. Oxy recruitment to the NRPS is mediated by a specialized recruitment domain known as the X‐domain, which is unique to GPA biosynthesis [16, 17, 18]. The Oxy enzymes have been shown to perform their oxidative crosslinking reactions in a specific order, beginning with the action of OxyB that installs the C‐*O*‐D crosslink [13, 15, 19, 20, 21]. The next enzyme to act is the F‐*O*‐G crosslinking enzyme OxyE, which is found in the biosynthesis of type III and IV GPAs [22, 23]. The last two crosslinking steps are performed by OxyA, which forms the D‐*O*‐E ring [18, 20, 21, 24], and finally OxyC installation of the strained A‐B ring [25, 26].

Recent advances in genome mining have led to the discovery of new GPAs, notably in the type II and V GPA families, and include molecules such as the kineomicins (type IIa) [11] or corbomycin (type V) [27, 28]. The type V GPA family (e.g., kistamicin [29] and complestatin [27, 28, 30], Figure 1A) is considerably different from type I–IV GPAs, with these GPAs bearing no glycosyl groups, and containing very different crosslinking patterns, such as the 4‐hydroxyphenylglycine (Hpg)‐tryptophan (Trp) (D‐E) ring in kistamicin and complestatin, and other exemplars with different crosslinks not found in the type I–IV GPAs [31, 32, 33]. The antibiotic activity of type V GPAs differs from Lipid II binding and instead prevents cell wall remodeling by inhibiting autolysin activity [28].

To understand the crosslinking of GPAs, in vitro chemoenzymatic assays have been established using a shortened peptidyl carrier protein/ X‐domain (PCP‐X) didomain from the final module of various NRPS assembly lines together with Oxy enzymes to perform crosslinking steps of GPA biosynthesis (Figure 1B) [18, 23, 25, 34]. This method relies on the ability to access GPA peptidyl‐coenzyme A (CoA) substrates via chemical methods [35, 36, 37] and to subsequently load these peptides enzymatically onto the apo‐PCP domain using a promiscuous mutant of the phosphopantetheinyl transferase Sfp [38]. While such peptidyl‐CoA substrates can typically be accessed for the more hydrophilic GPAs such as vancomycin, thanks to their improved solubility in aqueous buffers, challenges remain in synthesizing peptidyl‐CoA substrates for the more hydrophobic GPAs such as teicoplanin, actinoidin, or type V scaffolds (Figure 1A). Previous studies using kistamicin and teicoplanin peptides [23, 29] could partially overcome peptide hydrophobicity through the conversion of a peptide hydrazide to the corresponding peptidyl‐CoA using conditions first reported by Liu [39]. A recent study utilized an alternative total synthetic approach rather than solid‐phase peptide synthesis (SPPS) to synthesize a hydrophobic complestatin peptidyl‐CoA substrate [30], whilst earlier SPPS methods developed by the Robinson group had utilized temporary Alloc protection in place of Fmoc protection [13, 40, 41, 42, 43]. While peptidyl‐CoAs can be accessed using either solution‐based or solid‐phase methods, the levels of in vitro crosslinking using such peptides as substrates has remained limited, possibly due to the hydrophobicity of these substrates [29, ‍^30^]. To overcome the difficulties seen for hydrophobic GPA peptide sequences, here we report an improved synthetic route to access such substrates for in vitro GPA chemoenzymatic crosslinking assays that significantly improves the levels of enzymatic activity seen for these peptides.

## 2 Results and Discussion

### 2.1 Synthesis of GPA Peptidyl‐Coenzyme A Substrates via Modified Liu and Dawson Methodologies

**SCHEME 1:**
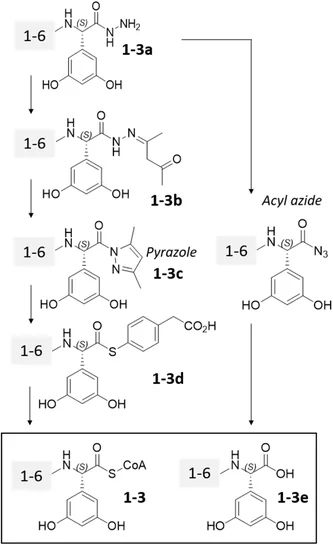
Synthetic routes and intermediate species involved in the synthesis of peptidyl‐CoA thioester conjugates using protocols commencing from peptidyl‐hydrazides adapted from Liu (acyl azide) [39] and Dawson (pyrazole) [45]. 1–6: hexapeptide sequence from teicoplanin (**1**), actinoidin (**2**), or kineomicin (**3**) GPAs. For reagents, see Scheme 2.

**SCHEME 2:**
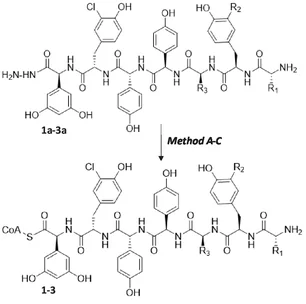
Methods to access peptidyl‐CoAs. Method A [39]: (i) NaNO_2_, 6 M GnHCl, pH 3, 15 min, −15 °C; (ii) CoA, GnHCl (pH 3), 15 min, −15 °C; (iii) NaOH 0.5 M until pH 7, 16 h, −15 °C to RT. Method B [45]: (i) acetylacetone, 6 M GnHCl, pH 3, 3 h, RT; (ii) MPAA, CoA, GnHCl (pH 3); (iii) NaOH 0.5 M until pH 7, RT; (iv) TCEP, GnHCl (pH ‍7), 16 h, RT. Method C: (i) acetylacetone, DMF, pH 3, 3 h, RT, then precipitation with diethyl ether; (ii) MPAA, CoA, GnHCl (pH 3); (iii) NaOH 0.5 M until pH 7, RT; (iv) TCEP, GnHCl (pH 7), 16 h, RT. Yields and identities of *R*_1_, *R*_2_, and *R*_3_ are defined in Table 1.

The fragment coupling between a protected peptide and unprotected coenzyme A raises two challenges: the epimerization of the C‐terminal residue and the limited solubility of either coenzyme A in an organic solvent or the peptide counterpart in an aqueous buffer. Consequently, the method initially chosen for the conversion of peptidyl‐hydrazides into peptidyl‐coenzyme A conjugates was adapted from conditions reported by Liu [39]. After synthesizing more than 50 different peptidyl‐CoA thioesters, we observed several problems with this method, namely the poor solubility of hydrophobic peptides into 6 M urea (or 6 M guanidium HCl) buffers and the sensitivity of several side chain functional groups to the sodium nitrite oxidation step. As a result, we decided to explore a different strategy to access GPA peptidyl‐CoA thioesters by replacing the sodium nitrite oxidation with formation of a Knorr‐pyrazole group, as this reaction is compatible with the use of organic solvents (Scheme 1) [44]. This reaction requires the addition of acetylacetone, which forms the corresponding hydrazone (**1–3b**) upon the reaction with the C‐terminal hydrazide (**1–3a**). Subsequent dehydration leads to the formation of the pyrazole group (**1–3c**), which can be displaced by arylthiols such as 4‐mercaptophenyl acetic acid (MPAA) to form the thioaryl ester (**1–3d**) [45, 46]. Herein, we report a comprehensive study regarding the optimization of formation of peptidyl‐coenzyme A conjugates (**1–3**) from GPA sequences and the difficulties related to the presence of the C‐terminal 3,5‐dihydroxyphenylglycine (l‐Dpg) residue [47]. After optimization of the synthesis route, we then tested the efficacy of the in vitro Oxy cyclization cascade with hydrophobic type II GPA peptides based on the structures of kineomicin and actinoidin (Figure 1A). SPPS was used to prepare all the desired peptide hydrazides using the teicoplanin peptide sequence (**1a**) as a proof of concept to optimize pyrazole formation with a C‐terminal Dpg residue. Next, the peptide sequences of actinoidin (**2a**) and kineomicin (**3a**) were synthesized to compare the yield of the different synthetic methods (Scheme 2). All peptides were synthesized on hydrazino‐2‐chlorotrityl resin, using DIC/Oxyma as coupling reagents in DMF and deprotection cycles using a solution of DBU in DMF (Figures S1–S3). Peptide hydrazides were cleaved from the resin using TFA and purified by reversed‐phase HPLC, leading to the isolation of peptide hydrazides **1a–3a**.

First, we synthesized peptidyl‐CoAs **1–3** using our previous methodology [35], which was adapted from the method reported by Liu (Scheme 2, Method A) [39]. We had previously identified that this method resulted in low isolated yields of teicoplanin‐CoA, which is commonly seen for GPA sequences containing a high proportion of hydrophobic, aromatic residues. Using the control teicoplanin sequence along with the type II GPA sequences of kineomicin and actinoidin, we again obtained a low yield of the corresponding peptidyl‐CoAs, with isolated yields under 10% after final purification (Table 1, Method A).

**TABLE 1:** Summary of the yields obtained for the preparation of 1–3 using Methods A–C. Method A (acyl azide intermediary), Method B (incomplete pyrazole formation in 6 M GnHCl pH 3), and Method C (pyrazole formation in DMF at pH 3). Positions of *R*_2_ and *R*_3_ are shown in Scheme 2.

**Peptide**	***R***_**1**_	***R***_**2**_	***R***_**3**_	**Method A**	**Method B**	**Method C**	**Fold difference**
							**Method C/A**
1	d‐Hpg	‒Cl	l‐Dpg	3.5%	17.5%	26.0%	× 7.4
2	d‐Hpg	‒H	l‐Phe	2.1%	N.D	6.3%	× 3.0
3	d‐Hpg	‒H	l‐Ile	6.9%	N.D	23.6%	× 3.9
5^a^	d‐Leu	‒Cl	l‐Asn	15.0%	N.D	N.D	N.D

a Previously reported yield [35].

**FIGURE 2:**
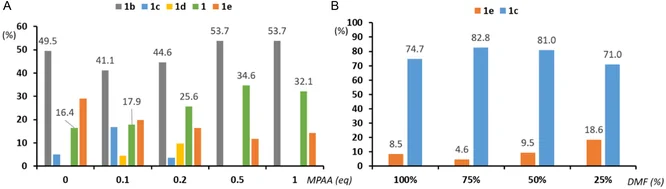
Summary of optimization experiments for the Knorr‐pyrazole synthesis of linear teicoplanin‐CoA (**1**—green). (A) The effects of altering the ratio of MPAA to the peptide, highlighting the incomplete conversion of the hydrazone (**1b**—gray). (B) The effect of different DMF/H_2_O mixtures on the level of conversion of peptidyl‐hydrazone (**1b**) into peptidyl‐pyrazole (**1c**—blue). Color code: thioarylester (**1d**—yellow); hydrolyzed peptide (**1e**—orange).

For our first attempts exploring the use of pyrazole chemistry, we utilized the teicoplanin hydrazide **1a** and maintained the equivalents of acetylacetone (2.5 eq) and the buffer (6 M GnHCl, pH 3) but varied the quantity of 4‐mercaptophenylacetic acid (MPAA) added to form the activated thioester. In native chemical ligation, which occurs between a C‐terminal thioester and a sequence bearing an N‐terminal cysteine residue, MPAA is often used in excess to assist the desired S─N acyl shift, generating a more reactive thioester intermediate. To access peptidyl‐CoAs using a Knorr‐pyrazole strategy, we instead opted to keep the equivalents of MPAA either below or at a stoichiometric ratio with the peptide hydrazide **1a** (Figures S4–S6). In all test reactions (Figure 2A), we observed the complete conversion of the hydrazide starting material (**1a**), with the majority forming the corresponding hydrazone (**1b**). When 0.5–1 eq of MPAA was added, the yield of the hydrolyzed product (**1e**) remains at around 10%, while the formation of the peptidyl‐CoA (**1**) corresponds to around 30% of the starting material (based on LC integration at 254 nm). Addition of MPAA also leads to almost complete consumption of pyrazole (**1c**) and thioaryl ester (**1d**). It appears, therefore, that incomplete formation of the pyrazole was the main obstacle to efficient yields of peptidyl‐CoAs via this route, possibly due to the presence of the bulky C‐terminal Dpg residue. Teicoplanin‐CoA (**1**) was isolated with a 17.5% yield, but the same strategy did not result in the isolation of **2** or **3** (Scheme 2, Table 1, Method B). This was due in part to the incomplete conversion into the pyrazole and the precipitation of the most hydrophobic intermediaries (**2–3c** and **2–3d**) in the reaction buffer.

#### 2.1.1 Conditions for the Complete Conversion Into Peptidyl‐Pyrazole

We next investigated various solvent conditions to maximize the conversion of the hydrazone **1b** into pyrazole **1c**. When using different aqueous DMF mixtures spiked with HCl to reach a pH 3 (Figure 2B), we could observe the complete conversion of the starting material **1a** (Figure S7). The results obtained when using DMF alone consistently afforded high conversions to pyrazole **1c** (>70%), with the proportion of hydrolysis increasing with water content, leading to 18.6% of hydrolyzed product **1e** when employing a ratio of DMF/H_2_O (25:75). We observed similar results when we used aqueous ACN mixtures containing 0.1% TFA (Figure S8). Overall, these conditions demonstrated that increased hydrolysis was observed when using >50% H_2_O in the solvent mixture.

#### 2.1.2 Conditions for Resolubilizing Peptidyl‐Pyrazoles in 6 M GnHCl Buffer

After optimizing peptidyl‐pyrazole formation, we explored if there were any effects of varying the arylthiol used for peptidyl‐CoA synthesis (Figure 2A). Using the teicoplanin pyrazole **1c** once more as our model system obtained in DMF at pH 3 (Figure S9), we selected 4 alternate arylthiols to test, including 4‐aminothiophenol (MPA), 4‐mercaptophenol (MP), 4‐methoxythiophenol (MMP), and a MPAA‐peptide (MPAA‍‐‍KR2, **4**).

**FIGURE 3:**
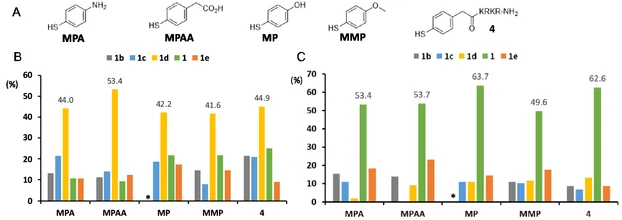
Summary of the usage of different arylthiols applied to the synthesis of the linear teicoplanin‐CoA **1**. (A) Arylthiols used in this study. (B) Proportion of all the intermediaries determined by LCMS after 1 h of reaction. (C) Proportion of all the intermediaries determined by LCMS after 15 h of reaction. Color code: hydrazone (**1b**—gray), pyrazole (**1c**—blue) thioarylester (**1d**—yellow), the peptidyl‐CoA (**1**—green), hydrolyzed peptide (**1e**—orange); * MP and **1b** are overlapping.

The MPAA‐peptide **4** was synthesized by SPPS (Figure S10), adding trityl‐mercaptophenylacetic acid [48] at the N‐terminus of a hydrophilic tetrapeptide sequence (KRKR). After a 1‐h (Figure 3B) or 15‐h reaction (Figure 3C), LCMS monitoring (Figures S11–S20) showed that all the arylthiols had similar effects on the conversion of peptidyl‐pyrazole **1c**. The major product observed at 1 h in all cases were the aryl thioesters (40%–55%), and the desired peptidyl‐CoA (**1**) was produced in high conversion at 15 h (>50% determined by LCMS). All conversions observed were also superior to the result obtained with the original MPAA arylthiol when using the original peptidyl‐pyrazole formation conditions in GnHCl at pH 3 (Figure 2A, entry 1 eq MPAA (32.1%) using Method B).

Given these results, we then utilized a synthesis route employing DMF with no added water to isolate the peptidyl‐pyrazoles (**1c–‍3c**) by precipitation in diethyl ether (Figures S9, S21, and S22). The compounds were resuspended in 6 M GnHCl (pH 3) containing MPAA (1 eq) as we observed no clear advantages in using other arylthiols. The isolated yields of the peptidyl‐CoAs **1–3** were then seen to be significantly improved (Table 1, Method C) for all peptide sequences. The actinoidin sequence (**2**) remains more challenging, due in part to a partial solubility of the peptidyl‐pyrazole (**2c**) in diethyl ether (Figure S23). Nonetheless, we isolated sufficient peptidyl‐CoAs (Figures S24–S26) to conduct chemoenzymatic assays for all probes thanks to this improved synthetic route, with 3–7 times higher yields observed compared to previous attempts (Table 1. Fold difference Method C/A).

### 2.2 Cytochrome P450‐Catalyzed Crosslinking of Type II GPA Peptides

The newly accessed type II/IIa GPA peptidyl‐CoAs (actinoidin and kineomicin) prepared using Methods A and C were subjected to enzymatic crosslinking assays after their initial loading on a PCP‐X didomain construct from teicoplanin biosynthesis [25] via the activity of the engineered variant of the phosphopantetheinyl transferase Sfp [38]. With the peptidyl‐PCP‐X substrates in hand, these were then subjected to sequential P450‐mediated crosslinking reactions, commencing with OxyB_bal_ (balhimycin, type I GPA), followed by OxyA_ris_ (ristomycin, type III GPA), and finally concluding with OxyC_cep_ (chloroeremomycin, type I GPA). These Oxy enzymes were chosen due to their performance in previous studies of the chemoenzymatic crosslinking of GPA peptides [25, 49, 50].

**FIGURE 4:**
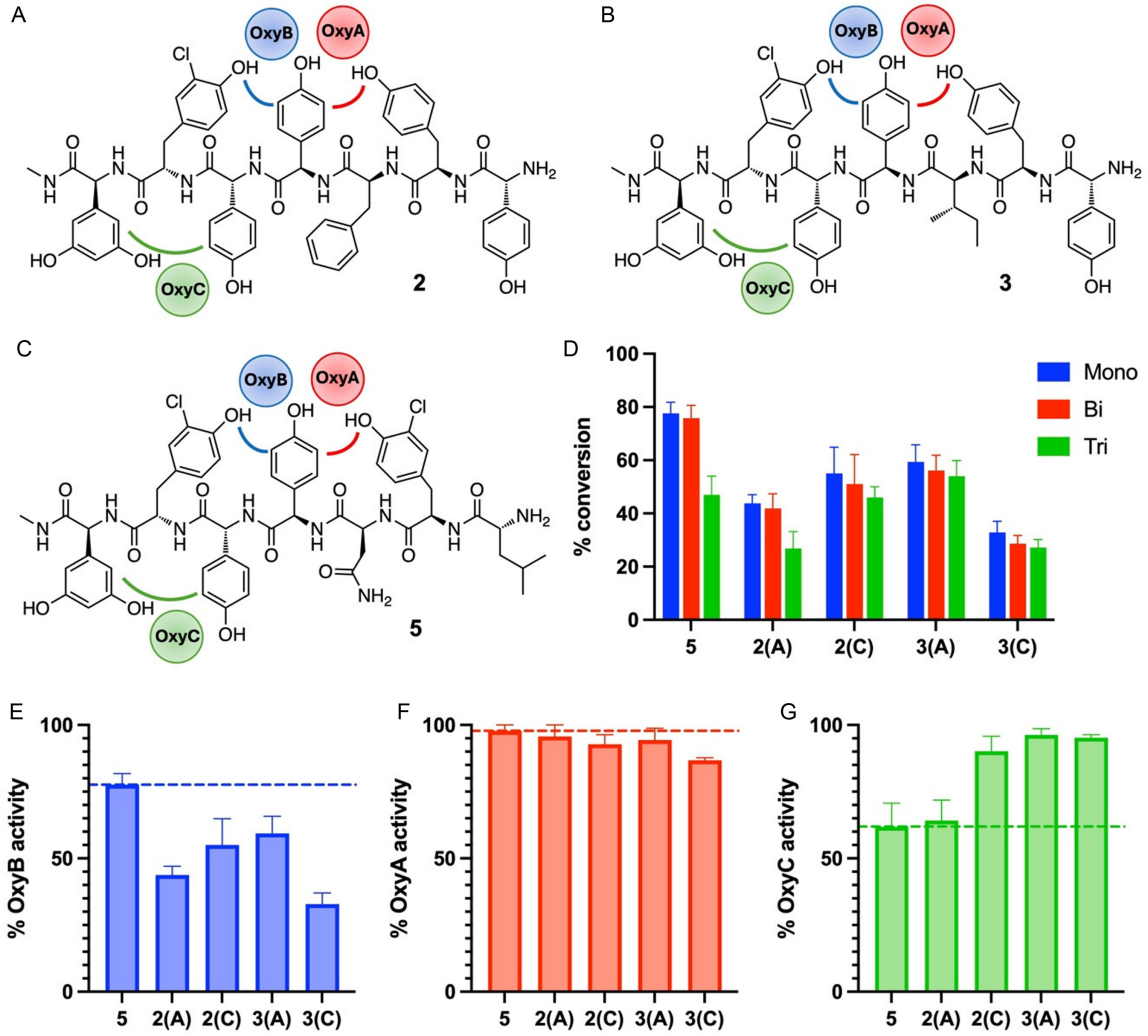
Enzymatic crosslinking assay data of PCP‐X didomain conjugates loaded with the peptidyl‐CoAs generated in this study, employing sequential addition of OxyB_bal_, OxyA_ris_, and OxyC_cep_. (A) Generic structure of peptide **2** with crosslinking positions shown. (B) Generic structure of peptide **3** with crosslinking positions shown. (C) Generic structure of peptide **5** with crosslinking positions shown. (D) % relative conversion to mono‐, bi‐, and tricyclic products for peptides **2** and **3** synthesized using method A or C compared to control (peptide **5**), error bars indicate SD, *n* = 3. (E) % OxyB_bal_ activity, error bars indicate SD, *n* = 3. (F) % OxyA_ris_ activity, error bars indicate SD, *n* = 3. (G) % OxyC_cep_ activity, error bars indicate SD, *n* = 3. % relative conversion and enzyme activity calculated based on area under the curve of TIC for *m/z* of respective products.

When compared to the standard type I GPA control (vancomycin), the type II GPA peptides show reduced OxyB_bal_ activity [25]. The probes do see high levels of subsequent OxyA_ris_ activity and show an unprecedented improvement in activity for the final enzyme in the crosslinking cascade, OxyC_cep_ (Figure 4). While the initial crosslinking of the vancomycin control peptide was lower than we had previously observed [25], possibly due to batch‐to‐batch variability during the heterologous expression, the more hydrophobic type II/IIa peptide substrates **2–3** showed a third to a half reduction in crosslinking compared to the standard (the crosslinking of substrate **1** was not tested again having been previously and extensively characterized). One possible explanation for this reduction in activity, aside from the bulky nature of the amino acid residues at positions 1 and 3 of the peptide, could be the lack of modifications in these type II peptides that are conserved in type I peptides, principally the lack of a chlorine substituent on the position 2 tyrosine residue of peptides **2–3** [51]. The importance of chlorine atoms on the conserved Tyr‐2 and Tyr‐6 residues for efficient Oxy enzyme activity has been previously observed [34, 52] and provides a possible explanation for this reduced OxyB activity. The high OxyA_ris_ activity seen with the type II substrates is not incompatible with this explanation, given that the position 2 tyrosine in the type III ristomycin backbone is not chlorinated [53]. The altered position 3 residue—phenylalanine (actinoidin) or isoleucine (kineomicin) compared to asparagine (vancomycin)—also does not seem to impact the crosslinking ability of OxyA_ris_, which is in line with previous experiments that have shown OxyA is more sensitive to changes in the C‐terminal region of the peptide as opposed to the N‐terminus where it installs the crosslink [16, 35, 54]. The most marked improvement noted in this study is the increase in OxyC_cep_ activity, which was observed for three of the four type II GPA samples tested. The single instance of low activity (Figure 4, peptide **2(A)**) suggests that peptide batch‐to‐batch and assay variabilities could still be plaguing specific assays when using these hydrophobic peptides, possibly exacerbated by the small scale of these enzymatic reactions (~8 μg). Nevertheless, the results seen with the majority of assays show the highest in vitro activity for an OxyC homolog reported to date, which is an important step toward a more effective chemoenzymatic route to GPA aglycones [23, 25, 26]. Also, the presence of l‐Hpg (avoparcin—OxyC ~40%) [30] and l‐Dpg (teicoplanin—OxyC ~35%) [23] in position 3 was previously tested and showed a clear reduction in OxyC activity when these aryl side chains were present compared to the sequences tested here (>60% OxyC). This could indicate an OxyC_cep_ preference for either shorter polar/alkyl residues (vancomycin—Asn; peptide library screen [25]—Ala, His, Thr) or large alkyl residues (actinoidin—Phe; kineomicin—Ile; peptide library screen [25]—Met, Leu) in position 3 of the GPA peptide sequence.

The improved activity shown by OxyC_cep_ in these experiments could also be due to the altered peptide structures in the circumstance that partially crosslinked intermediates do not act as potent inhibitors of the other Oxy homologs; this phenomenon has been noted for type I and type IV peptides, perhaps due to the bulk of the P1 and P3 side chains. A further explanation for the improved OxyC_cep_ activity can be found in the evolution of Lipid II binding GPAs, which has shown that the ancestor of the GPAs found today was comprised completely of aromatic residues [5]. Given the properties of this ancestral peptide sequence, it remains possible that such substrates are generally well tolerated by the Oxy enzymes, which have also evolved from those that crosslinked the hydrophobic, purely aromatic ancestral GPA.

## 3 Conclusion

Despite almost a decade passing since the first complete chemoenzymatic crosslinking cascade was reported for GPAs, many challenges remain with the in vitro cyclization of hydrophobic GPA peptide substrates. While a great increase in the diversity of these hydrophobic GPA scaffolds has been reported, including the expansion of the diversity of the type V GPAs and the discovery of the new type IIa GPA, kineomicin, understanding the crosslinking of these GPAs has been hindered by significant difficulties in substrate peptidyl‐CoA synthesis. In this work, peptidyl‐CoA substrates were accessed via a peptidyl‐pyrazole intermediate rather than through oxidation of a peptidyl‐hydrazide, with this optimized synthetic route providing improved yields of peptidyl‐CoAs by up to 7.4‐fold. In addition to improved access of peptidyl‐CoA substrates for the type II/IIa GPAs, actinoidin and kineomicin, a drastic improvement of OxyC_cep_ activity was observed compared to that for these experiments when compared to the crosslinking seen for the type I GPA, vancomycin. The OxyC_cep_ activity reported here is the highest in vitro crosslinking conversion observed to date, possibly due to a combination of improved substrate synthesis and peptide structural features. The improved synthetic route reported here opens the door to explore the in vitro biosynthesis and cytochrome P450‐catalyzed crosslinking of more hydrophobic GPA systems that have remained relatively unexplored until now. This will be particularly important in efforts to understand the complex multistep crosslinking of the type V GPAs such as kistamicin [29, 55] and the newly discovered type V GPAs such as those found in the fumamycins, rimomycins, and varsomycins [31, 32, 33].

## Funding

This study was supported by the Australian Research Council (CE200100012).

## Conflicts of Interest

The authors declare no conflicts of interest.

## Supporting information

Supplementary Material

## Data Availability

The data that support the findings of this study are available from the corresponding author upon reasonable request.
