# Supplementary material for: Synthesis of Glycopeptide Antibiotic Peptide Substrates Using Knorr‐Pyrazole Chemistry
Source: ChemMedChem. 2026 Sep 5;21(17):e70487. doi: 10.1002/cmdc.70487 (PMC13546370; doi:10.1002/cmdc.70487)
Supplement: Supplementary file 1 [file CMDC-21-e70487-s001.pdf]

Supporting Information

Synthesis of glycopeptide antibiotic peptide substrates using Knorr-pyrazole chemistry

Jemma Gullick,[a,b] W. Ramani H. Weerasinghe,[a,b] Ralf B. Schittenhelm,[a,c] Julien Tailhades[a,b]* and Max J. Cryle[a,b]*

[a] Department of Biochemistry and Molecular Biology, The Monash Biomedicine Discovery Institute, Monash University, Clayton, VIC 3800, Australia. E-mail: julien.tailhades@monash.edu, max.cryle@monash.edu;

[b] ARC Centre of Excellence for Innovations in Peptide and Protein Science.

[c] The Monash Proteomics and Metabolomics Platform, Monash University, Clayton, VIC 3800, Australia.

Supplementary Methods 2

Chemicals and reagents 2

Preparation of Fmoc-amino acids 2

Preparation of resin 2

Solid-phase peptide synthesis 2

Peptidyl thioester synthesis 3

LCMS characterisation 3

Preparative RP-HPLC 4

Protein expression and purification 4

Enzymatic crosslinking reactions 4

HRMS characterisation 5

Supplementary Figures 6

Supplementary References 53

Supplementary Methods

Methods

Preparation of Fmoc-amino acids.[1] To a solution of unprotected amino acid (12 mmol) in 1:1 acetonitrile/10% Na_2CO_3 (50 mL) stirring at room temperature was added Fmoc-OSu (4.8 g, 14 mmol) in acetonitrile (50 mL) dropwise over 30 min at 4 °C. The solution was stirred overnight before being diluted with 1 M HCl (100 mL) and extracted with ethyl acetate (3 x 100 mL). The combined organic layers were washed with brine (2 x 100 mL), dried over Na_2SO_4 and the solvent removed in vacuo after filtration of Na_2SO_4. The crude products were recrystallized from ethyl acetate (50 mL) with hexanes added to the point of precipitation for Fmoc-D-Hpg-OH. Fmoc-L-Dpg-OH and Fmoc-L/D-Tyr(3-Cl)-OH was obtained after sonication of the crude powder into a 1:9 mixture of diethyl ether/hexane to remove the Fmoc derivatives (2x).

Synthesis of 4-(tritylmercapto)phenylacetic acid (4).[2] Adapted from the protocol reported by Offer et al., trityl chloride (1.0 g, 3.59 mmol) was added to a suspension of 4-mercaptophenylacetic acid (0.559 g, 3.32 mmol) in DCM (10 mL), and stirred overnight at room temperature. Next, the reaction was quenched with the addition of water (10 mL) and extracted with ethyl acetate (3 x 20 mL). The organic phase was washed with brine (3 x 20 mL), dried over sodium sulphate and the solvent evaporated *in vacuo*. Flash chromatography (100% hexane to 100% ethyl acetate) afforded 4-(tritylmercapto)phenylacetic acid (**4**) (0.85 g, 58%) as a white solid.

Preparation of resin.[3] 2-chlorotrityl chloride resin (200 mg) was swelled in DCM (8 mL, 30 min), washed with DMF (3x), and incubated with a 5% hydrazine solution in DMF (6 mL, 2 x 30 min). The resin was washed with DMF (3x) and a solution of DMF/TEA/MeOH (7:2:1) (4 mL, 15 min) was added to cap unreacted 2-chlorotrityl groups. Fmoc-L-Dpg-OH (30 mg, 0.075 mmol) was coupled to the resin using DIC (11.6 mL, 0.075 mmol) and Oxyma (21 mg, 0.15 mmol) overnight at room temperature. Next, unreacted hydrazine or amino groups were capped with Boc-glycine (26 mg, 0.15 mmol) that had been activated using DIC (33.2 mL, 0.15 mmol) and Oxyma (42 mg, 0.3 mmol) for 1 h for the linear GPA precursors containing unprotected phenolic groups.

Solid phase peptide synthesis.[4] Using Fmoc-L-Dpg pre-loaded resin, iterative cycles of amino acid addition were performed using a 1% DBU solution (3 mL, 3 x 30 s) in DMF for Fmoc removal, washing with DMF (3x), coupling of the subsequent Fmoc-protected amino acid (0.15 mmol) after activation with DIC (0.15 mmol) and Oxyma (0.3 mmol) for 5 min at

room temperature, followed by 10 min at 50 °C (conventional heating) and washing with DMF (3x). Fmoc-D-Hpg-OH and Fmoc-L-Dpg-OH were pre-activated at 4 °C. Cleavage of the peptidyl hydrazide from resin and removal of protecting groups (tBu, Boc, Trt) was accomplished using a solution of TFA/TIS/H_2O (95:2.5:2.5 v/v′/v″, 4 mL) with shaking at room temperature for 1 h. All peptides were concentrated under a stream of nitrogen to ~1 mL volume and precipitated with ice-cold diethyl ether (9 mL). The peptide products were washed by centrifugation (3x) and collected by centrifugation in a flame-resistant centrifuge.

Peptidyl thioester synthesis

Buffer 1: GnHCl (6 M) and NaH_2PO_4 (0.2 M), pH 3 (obtained via addition of HCl).
Buffer 2: GnHCl (6 M) and NaH_2PO_4 (0.2 M), pH 7.4 (obtained via addition of NaOH).

Method A (acyl azide – Liu et al.).[3, 5] The purified peptidyl hydrazide (**1a-3a**) was dissolved in buffer 1 to a final concentration of 5 mM. The solution was cooled to −15 °C using a salt/ice bath. Subsequently, 0.5 M $NaNO_2$ (0.95 equivalents) was added, and the mixture stirred for 10 min. Then, coenzyme A (1.2 eq) was added to the reaction. After 15 minutes, the pH was slowly adjusted to 7 using NaOH (0.5 M). The reaction mixture was stirred on ice for 2 h, before the final peptidyl-coenzyme A product was purified using preparative RP-HPLC, gradient 15−55% ACN over 40 min.

Method B (original pyrazole – Dawson et al.).[6] The crude peptidyl-hydrazide (**1a**) was dissolved in buffer 1 to a final concentration of 5 mM. Subsequently, acetylacetone (2.5 eq) was added and the reaction was stirred for 3 h at room temperature. Next, MPAA (1 eq) and coenzyme A (1.2 eq) were added as a solution made with buffer 1. After 1 h, TCEP (10 eq dissolved in buffer 2 – pH adjusted to 7.4) was added to reduce any disulfide bonds. After 15 hours at room temperature, another solution of TCEP (10 eq in dissolved buffer 2 – pH adjusted to 7.4) was added and **1** was isolated after preparative RP-HPLC purification.

Method C. Purified peptidyl-hydrazide (**1a-3a**) was dissolved in DMF at pH 3 (obtained via addition of HCl) to a final concentration of 5 mM. Subsequently, acetylacetone (2.5 eq) was added and the reaction was stirred for 3 h at room temperature. The peptidyl-pyrazoles (**1c-3c**) were precipitated with ice-cold diethyl ether (1.2 mL). Then MPAA (1 eq) and coenzyme A (1.2 eq) were added as a solution made with buffer 1. After 1 h, TCEP (10 eq dissolved in buffer 2 – pH adjusted to 7.4) was added to reduce any disulfide bonds. After 15 hours at room temperature, another solution of TCEP (10 eq in dissolved buffer 2 – pH adjusted to 7.4) was added, and **1-3** was isolated after preparative RP-HPLC purification.

LCMS characterisation. All LCMS analyses were carried out on a Shimadzu high performance liquid chromatography system coupled to mass spectrometer LCMS-2020 (ESI, operating both in positive and negative mode) equipped with an SPD-20A Prominence photo diode array detector and a LC-20AD solvent delivery module. Analytical runs were performed using an Agilent C18 column (300SB-C18, 5µm, 4.6 x 250 mm) with detection at 214 nm and 254 nm. LCMS-grade solvents were used: water + 0.1% FA (solvent A) and ACN + 0.1% FA (solvent B).

Preparative RP-HPLC. All purifications were performed using a Shimadzu high performance liquid chromatography system equipped with an SPD-M20A Prominence photo diode array detector and two LC-20AP pumps. Preparative runs were performed using an Agilent Zorbax SB-C18 column (7 µm, 21.2 x 250 mm) with a flow rate of 10 mL/min. Solvents used: water + 0.1% TFA (solvent A) and HPLC-grade ACN + 0.1% TFA (solvent B).

Protein expression and purification. Sfp (R4-4 mutant),[7] PCP-X_{tei} (Tcp12, UniProt ID: Q70AZ6), $OxyB_{bal}$ (UniProt ID: O87674), $OxyA_{ris}$ (UniProt ID: A0A075V230), $OxyC_{cep}$ (UniProt ID: O52825 and O52816),[8-9] PuR (UniProt ID: Q6N3B2) and PuxB A105V mutant (UniProt ID: Q6N2U2)[10] were expressed and purified as previously reported.

Enzymatic crosslinking reactions. Enzymatic crosslinking reactions were performed as previously described.[8-9] In brief, peptidyl-CoA (45 μM) was loaded onto PCP-X_{tei} (30 μM) using Sfp (R4-4 mutant, 4.5 μM) in a buffer system of HEPES (50 mM, pH 7.0), $MgCl_2$ (10 mM) and NaCl (50 mM) for one hour at 30 °C. Unloaded peptidyl-CoA was washed three times by filtration (Amicon® Ultra-0.5 mL centrifugal filters, 50,000 MWCO, Merck-Millipore). The loaded PCP-X_{tei} was split into three, ensuring that triplicate reactions contained the same amount of loaded PCP-X_{tei}, and used as substrate for subsequent cytochrome P450-catalysed crosslinking. A reaction solution of $OxyB_{bal}$ (0.5 μM), PuR (0.66 μM), PuxB A105V (2.5 μM), glucose (0.33%), glucose dehydrogenase (0.033 mg/mL), cysteamine (250 μM), TCEP (2500 μM) and NADH (2 mM) were added to the peptide loaded PCP-X_{tei} to a total volume of 105 μL and incubated at 30 °C for 20 minutes with 300 rpm shaking. $OxyA_{ris}$ (1 μM) was then added to the reaction mixture, and incubated for a further 20 minutes, before the addition of $OxyC_{cep}$ (2 μM). The reaction was incubated at 30 °C with 300 rpm shaking overnight. Following this, peptides were cleaved from the PCP-X_{tei} by the addition of methylamine (40% in H_2O, 15 μL). This was left for 15 minutes before the cleavage was quenched by the addition of water + 0.1% FA (850 μL). After this, the cleaved peptide was purified using pre-equilibrated solid phase extraction (SPE) columns (Phenomenex Strata^{TM}-X 33 μM Polymeric Reversed Phase, 10 mg, 1 mL). Columns were activated with methanol + 0.1% FA (1 mL), followed by the addition of water + 0.1% FA (1 mL) to equilibrate the column. The sample was then loaded

onto the column and washed with water + 0.1% FA (1 mL) and the cleaved peptides were eluted with methanol + 0.1% FA (0.5 mL). Cleaved peptides were concentrated, resuspended in 10% ACN/H_2O + 0.1% FA (40 μL) and analysed by HRMS. Conversion rates to monocyclic, bicyclic and tricyclic products were calculated by measuring the area under the curve of the TIC of the m/z of the linear, monocyclic, bicyclic and tricyclic peaks, written as linear, mono, bi and tri in the equations below. The % conversions and activity levels of enzymes were then calculated as follows:

$$\%\ conversion\ (monocyclic) = \frac{(mono + bi + tri) \times 100}{linear + mono + bi + tri}$$

$$\%\ conversion\ (bicyclic) = \frac{(bi + tri) \times 100}{linear + mono + bi + tri}$$

$$\%\ conversion\ (tricyclic) = \frac{tri \times 100}{linear + mono + bi + tri}$$

$$OxyB\ activity = \%\ conversion\ (monocyclic)$$

$$OxyA\ activity = \frac{\%\ conversion\ (bicyclic) \times 100}{\%\ conversion\ (mono)}$$

$$OxyC\ activity = \frac{\%\ conversion\ (tricyclic) \times 100}{\%\ conversion\ (bicyclic)}$$

HRMS analysis. High-resolution mass spectrometry measurements were performed on an Orbitrap Eclipse Tribrid mass spectrometer (Thermo Scientific) coupled to a Vanquish Neo UHPLC (Thermo Scientific), an Acclaim PepMap RSLC analytical column (75 µm × 50 cm, nanoViper, C18, 2 µm, 100Å; Thermo Scientific), and an Acclaim PepMap 100 trap column (100 µm × 2 cm, nanoViper, C18, 5 µm, 100Å; Thermo Scientific). The peptides were separated by increasing the concentrations of 80% acetonitrile/0.1% formic acid at a flow rate of 250 nL/min over 54 min. The instrument was operated in alternating data-dependent acquisition (DDA) and parallel reaction monitoring (PRM) cycles such that 10 MS2 scans were triggered per survey MS1 scan, followed by several targeted MS2 scans to ensure fragmentation of predefined, sample-dependent m/z precursors. Each survey MS1 scan (375−1575 m/z) was acquired with a resolution of 60,000 and a normalized AGC (automatic gain control) target of 200%. Dynamic exclusion was set to 30 s after one occurrence. The 10 most intense ions were selected for HCD fragmentation (normalized collision energy mode, 30% HCD collision energy) with a resolution of 30,000, an absolute AGC target of 5e4, and a dynamic scan range based on precursor m/z. Subsequent targeted MS2 scans were acquired with essentially identical settings. The raw data files were analyzed with QualBrowser

(XCalibur 3.0.63, Thermo Scientific) to view spectra and generate extracted ion chromatograms.

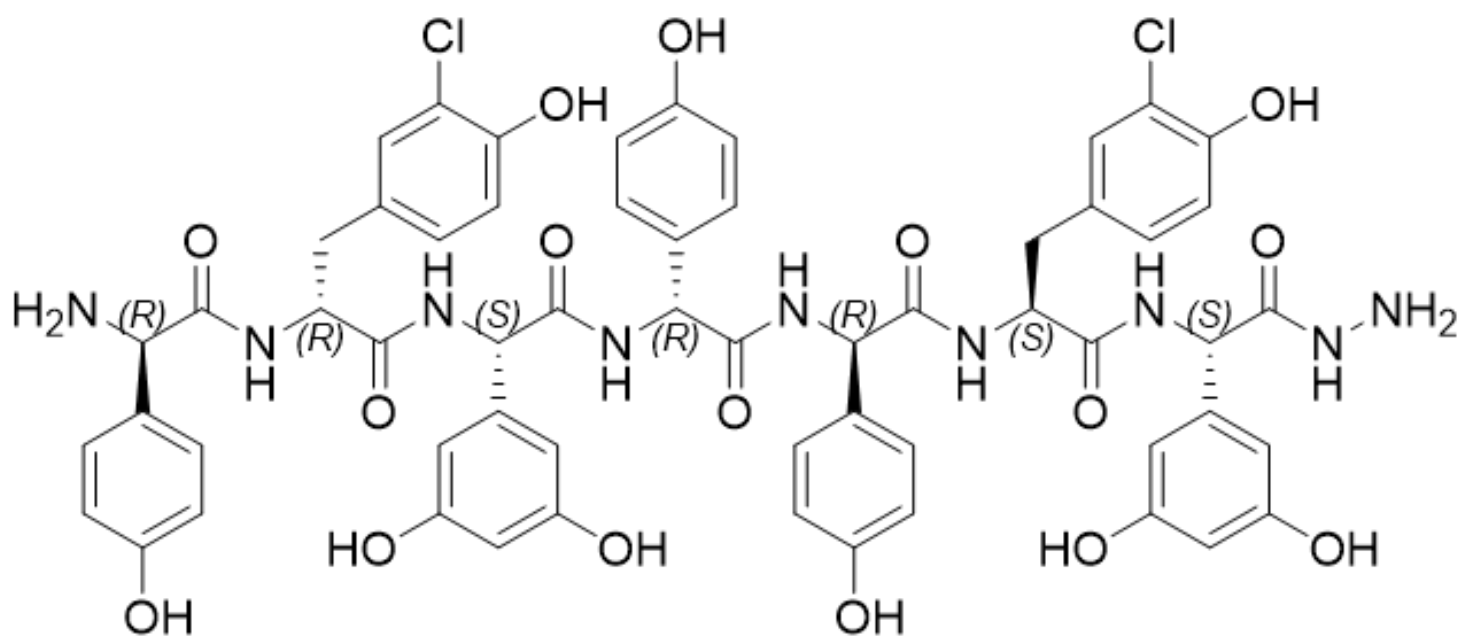

1a

Chemical Formula: $C_{58}H_{55}Cl_2N_9O_{16}$
Exact Mass: 1203.31

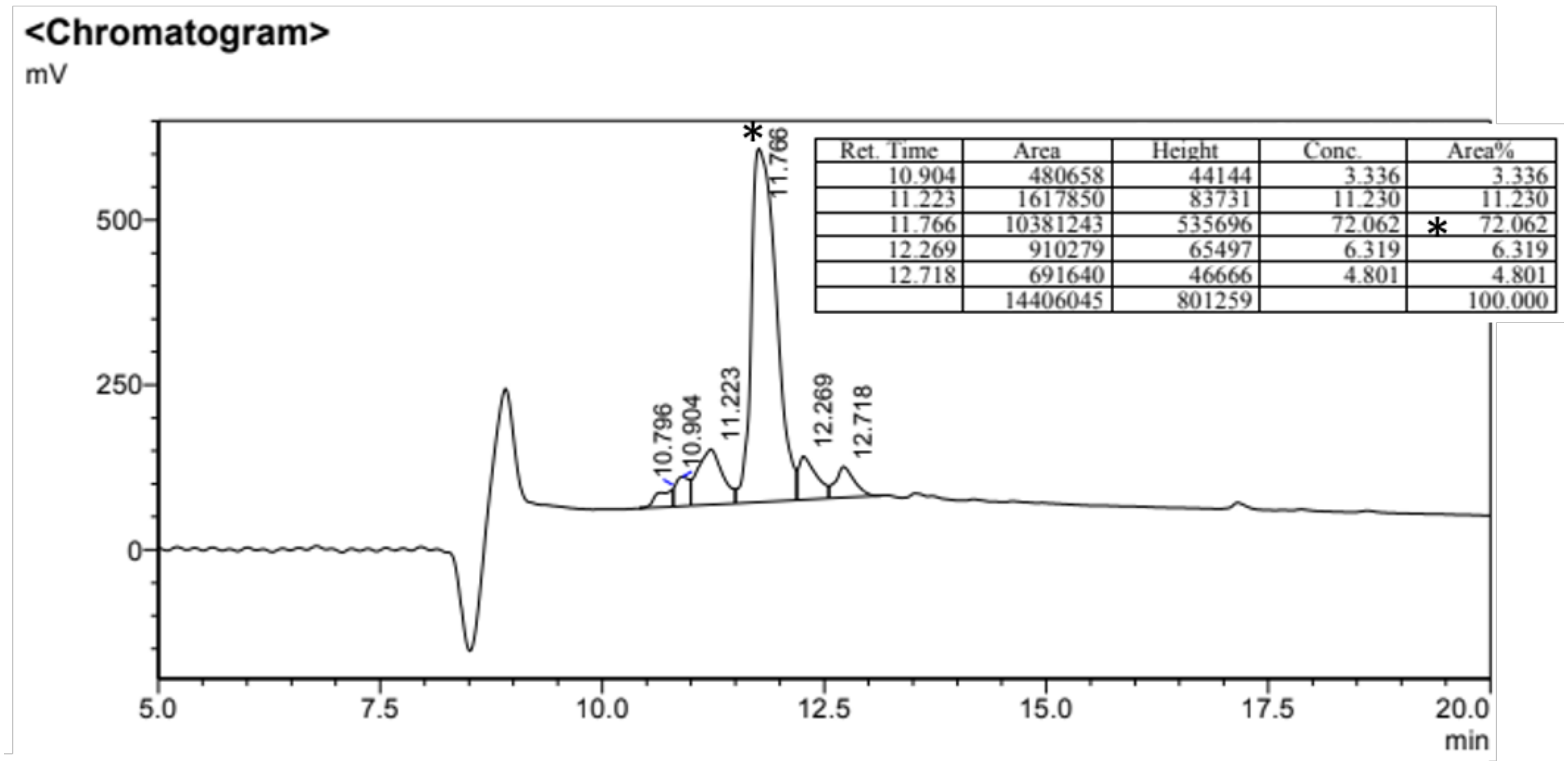

Ret. Time	Area	Height	Conc.	Area%
10.904	480658	44144	3.336	3.336
11.223	1617850	83731	11.230	11.230
11.766	10381243	535696	72.062	* 72.062
12.269	910279	65497	6.319	6.319
12.718	691640	46666	4.801	4.801
	14406045	801259		100.000

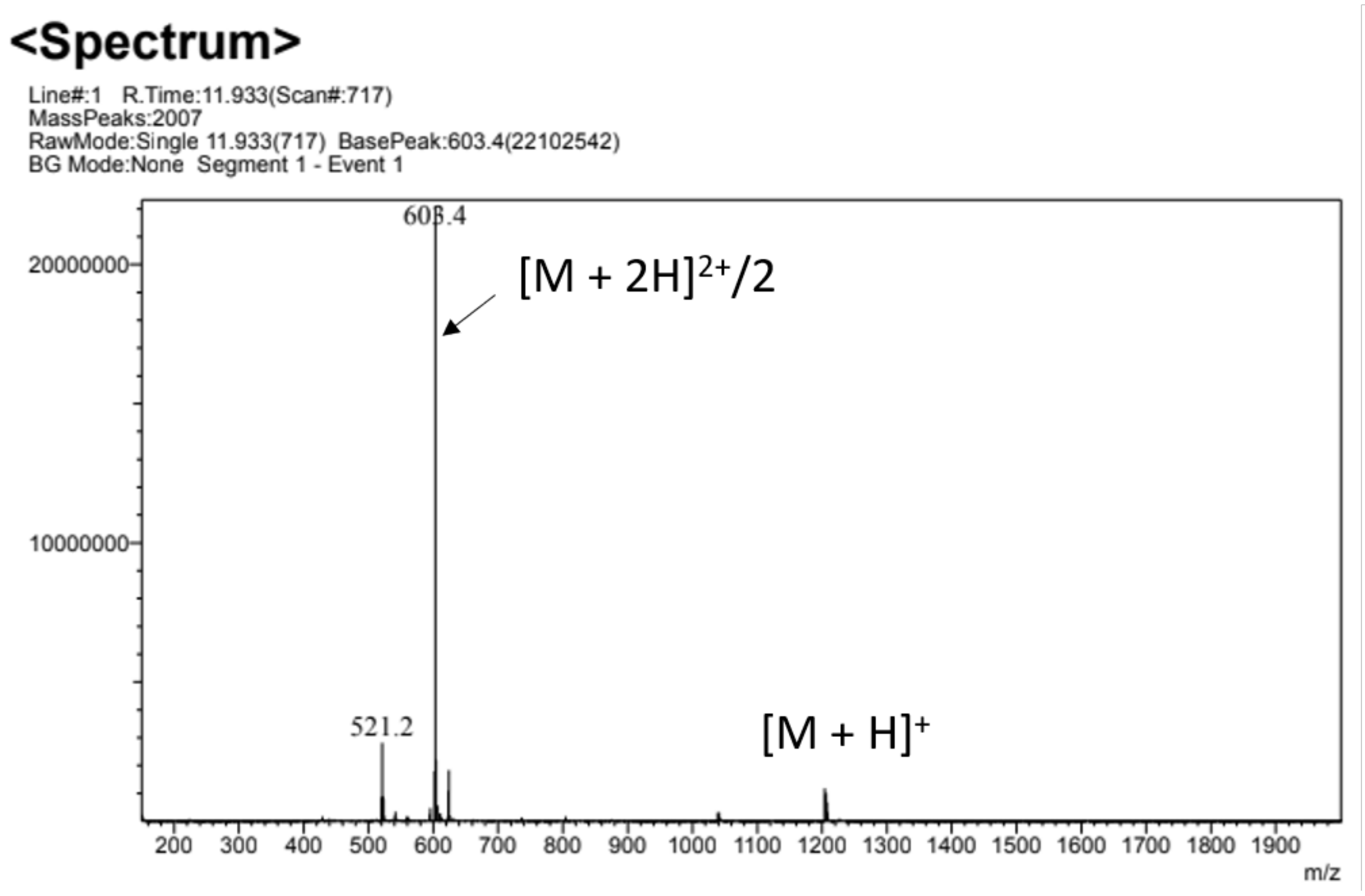

SI Figure 1. LCMS chromatograms of the linear teicoplanin peptide hydrazide (**1a**).

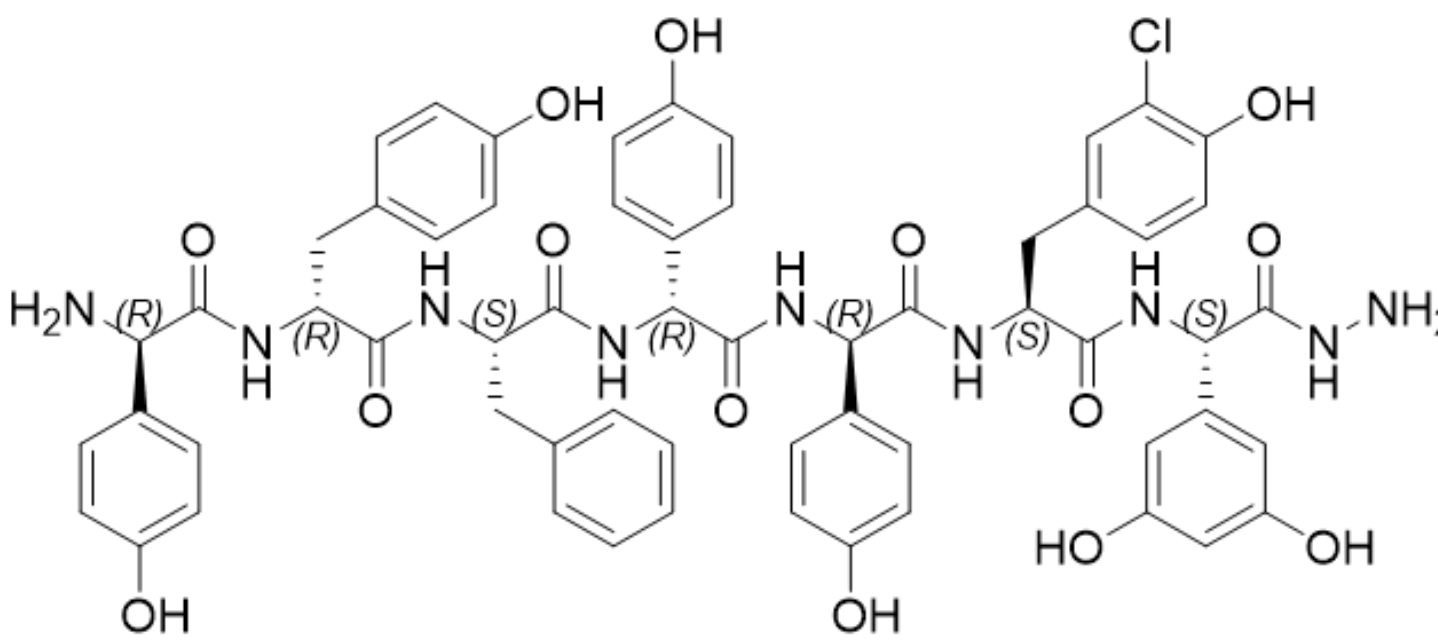

2a

Chemical Formula: $C_{59}H_{58}ClN_9O_{14}$
Exact Mass: 1151.38
Molecular Weight: 1152.61

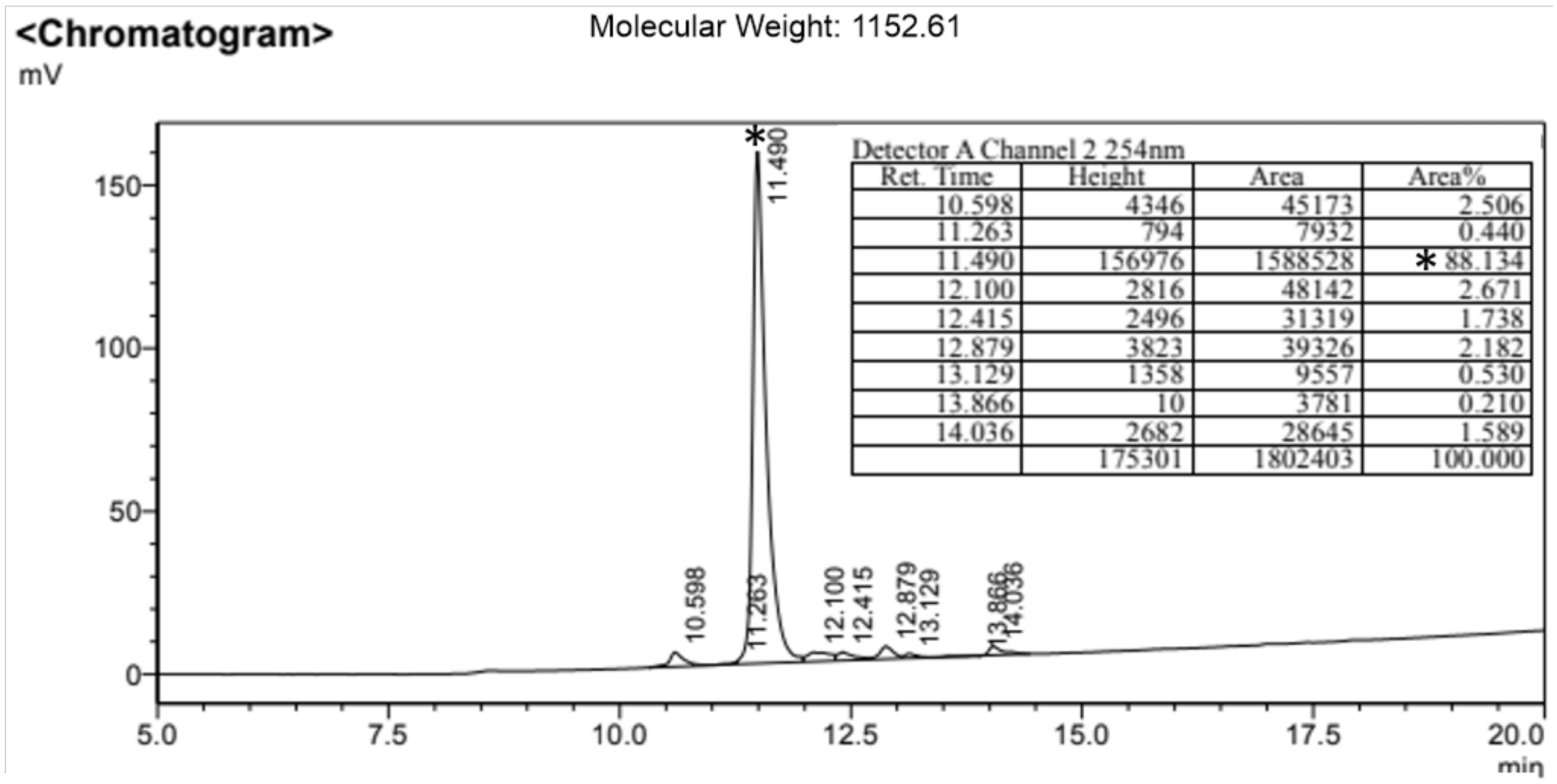

Detector A Channel 2 254nm

Ret. Time	Height	Area	Area%
10.598	4346	45173	2.506
11.263	794	7932	0.440
11.490	156976	1588528	* 88.134
12.100	2816	48142	2.671
12.415	2496	31319	1.738
12.879	3823	39326	2.182
13.129	1358	9557	0.530
13.866	10	3781	0.210
14.036	2682	28645	1.589
	175301	1802403	100.000

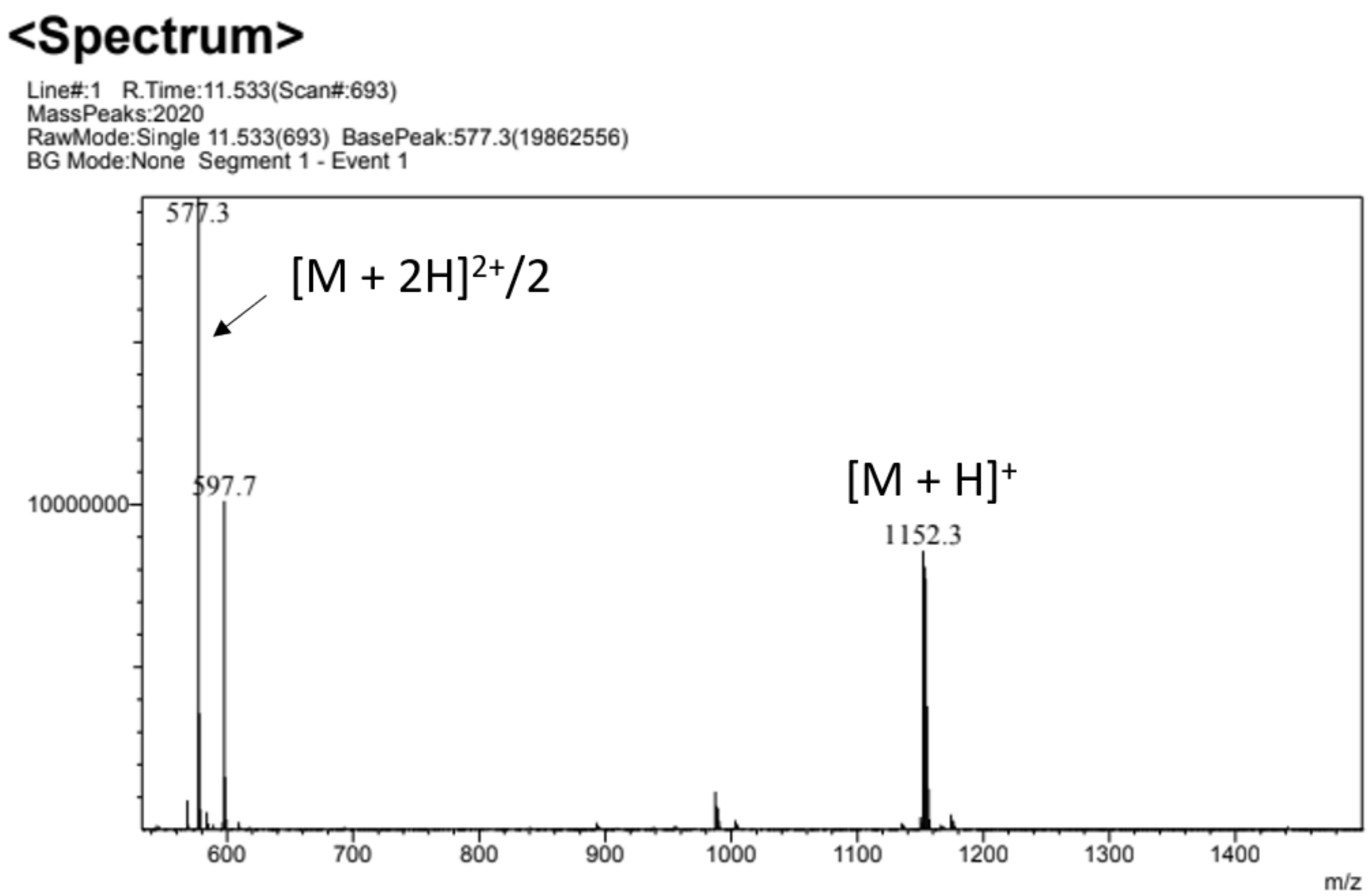

SI Figure 2. LCMS chromatograms of the linear actinoidin peptide hydrazide (**2a**).

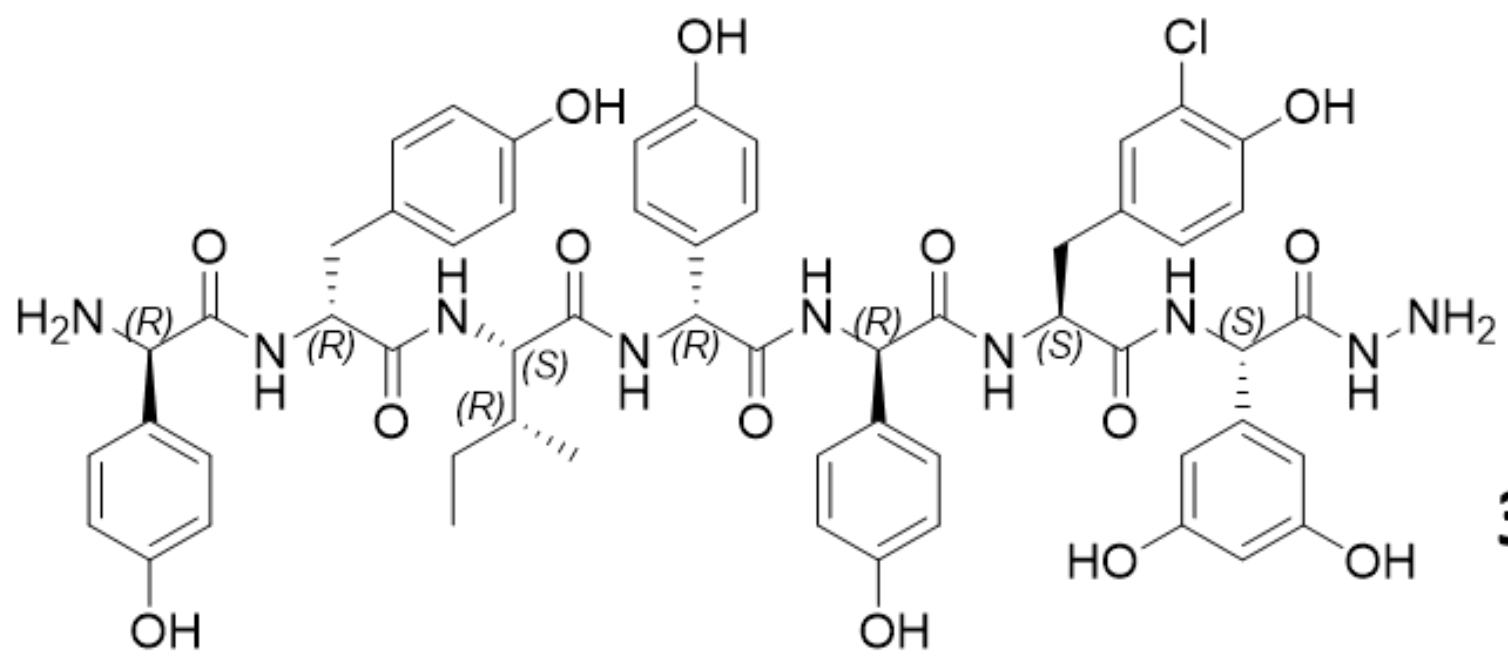

3a

Chemical Formula: $C_{56}H_{60}ClN_9O_{14}$
Exact Mass: 1117.39
Molecular Weight: 1118.60

<Chromatogram>

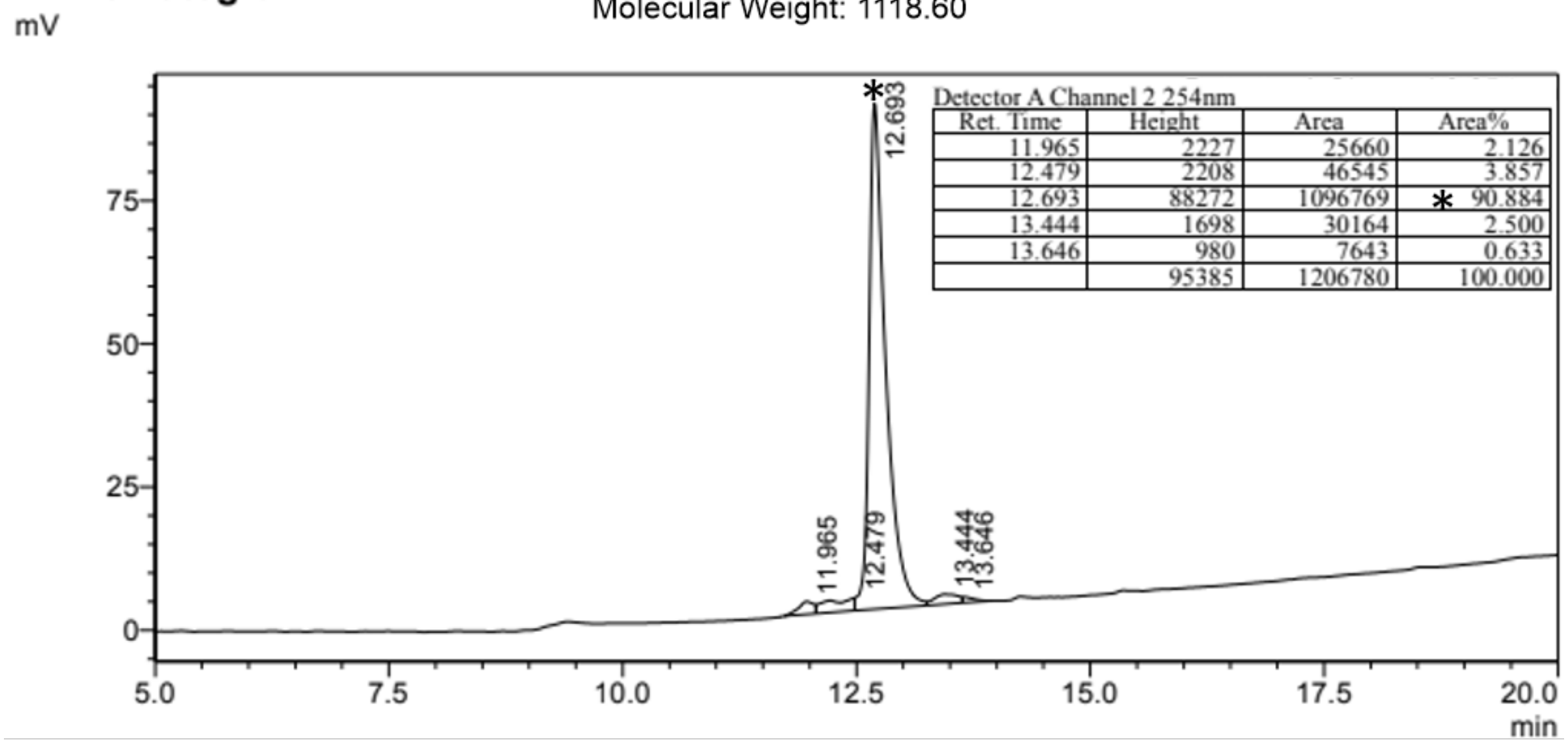

Detector A Channel 2 254nm

Ret. Time	Height	Area	Area%
11.965	2227	25660	2.126
12.479	2208	46545	3.857
12.693	88272	1096769	* 90.884
13.444	1698	30164	2.500
13.646	980	7643	0.633
	95385	1206780	100.000

<Spectrum>

Line#:1 R.Time:12.800(Scan#:769)
MassPeaks:2041
RawMode:Single 12.800(769) BasePeak:560.4(21347281)
BG Mode:None Segment 1 - Event 1

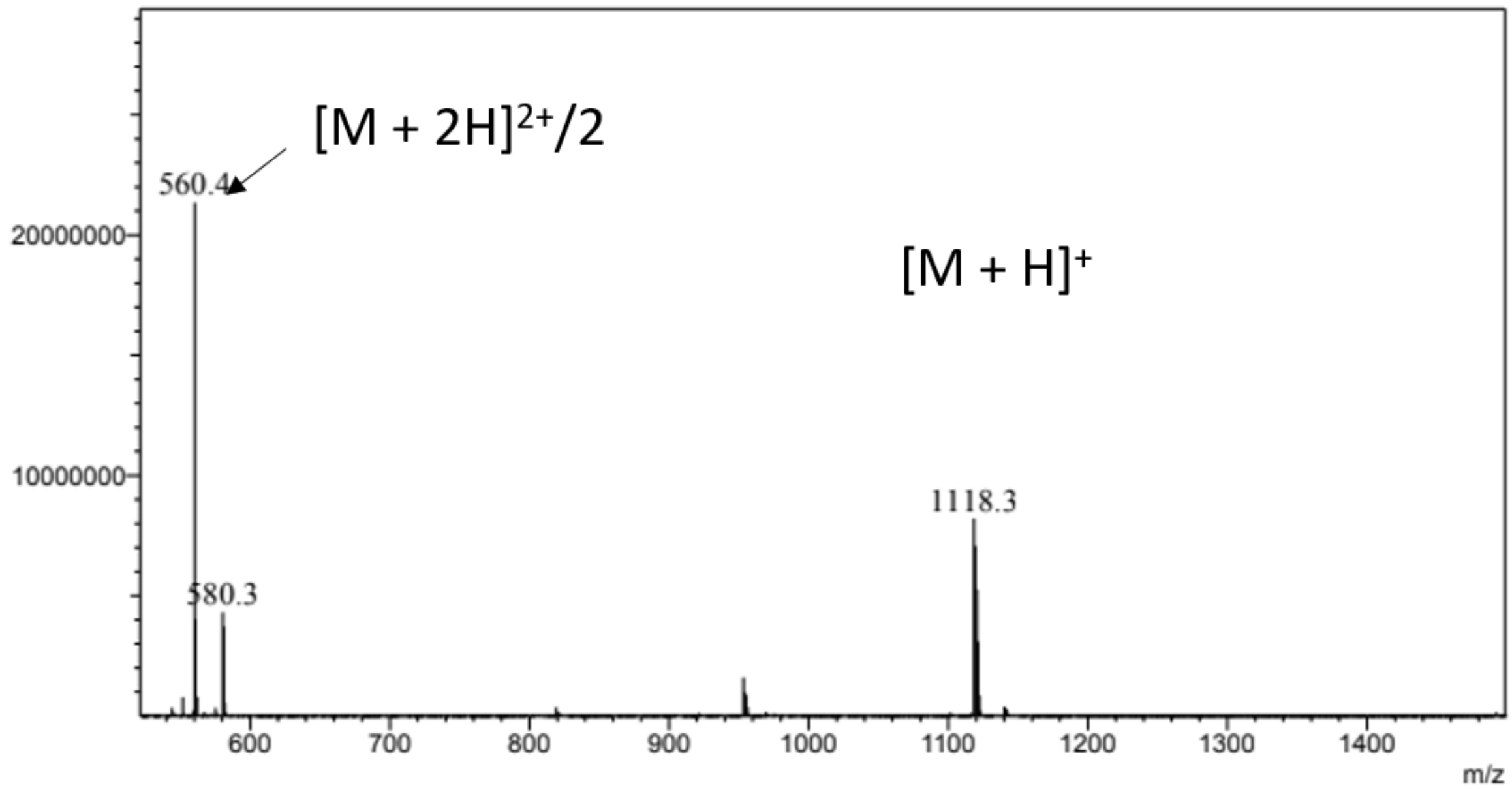

SI Figure 3. LCMS chromatograms of the linear kineomicin peptide hydrazide (**3a**).

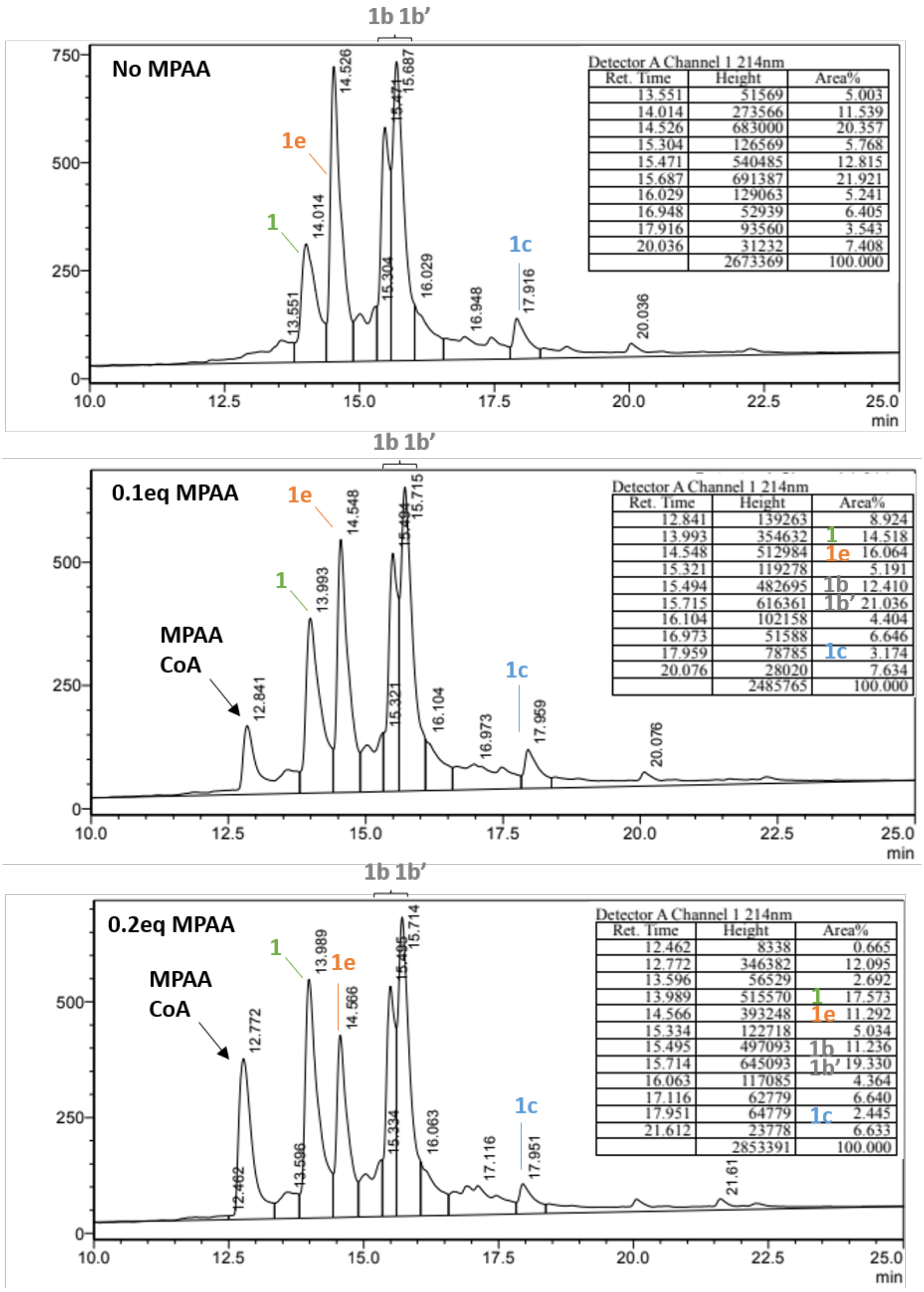

Detector A Channel 1 214nm (No MPAA)

Ret. Time	Height	Area%
13.551	51569	5.003
14.014	273566	11.539
14.526	683000	20.357
15.304	126569	5.768
15.471	540485	12.815
15.687	691387	21.921
16.029	129063	5.241
16.948	52939	6.405
17.916	93560	3.543
20.036	31232	7.408
	2673369	100.000

Detector A Channel 1 214nm (0.1eq MPAA)

Ret. Time	Height		Area%
12.841	139263		8.924
13.993	354632	1	14.518
14.548	512984	1e	16.064
15.321	119278		5.191
15.494	482695	1b	12.410
15.715	616361	1b’	21.036
16.104	102158		4.404
16.973	51588		6.646
17.959	78785	1c	3.174
20.076	28020		7.634
	2485765		100.000

Detector A Channel 1 214nm (0.2eq MPAA)

Ret. Time	Height		Area%
12.462	8338		0.665
12.772	346382		12.095
13.596	56529		2.692
13.989	515570	1	17.573
14.566	393248	1e	11.292
15.334	122718		5.034
15.495	497093	1b	11.236
15.714	645093	1b’	19.330
16.063	117085		4.364
17.116	62779		6.640
17.951	64779	1c	2.445
21.612	23778		6.633
	2853391		100.000

SI Figure 4. LC chromatogram and table data linked to **Figure 2A** (entries 0, 0.1, and 0.2 eq of MPAA).

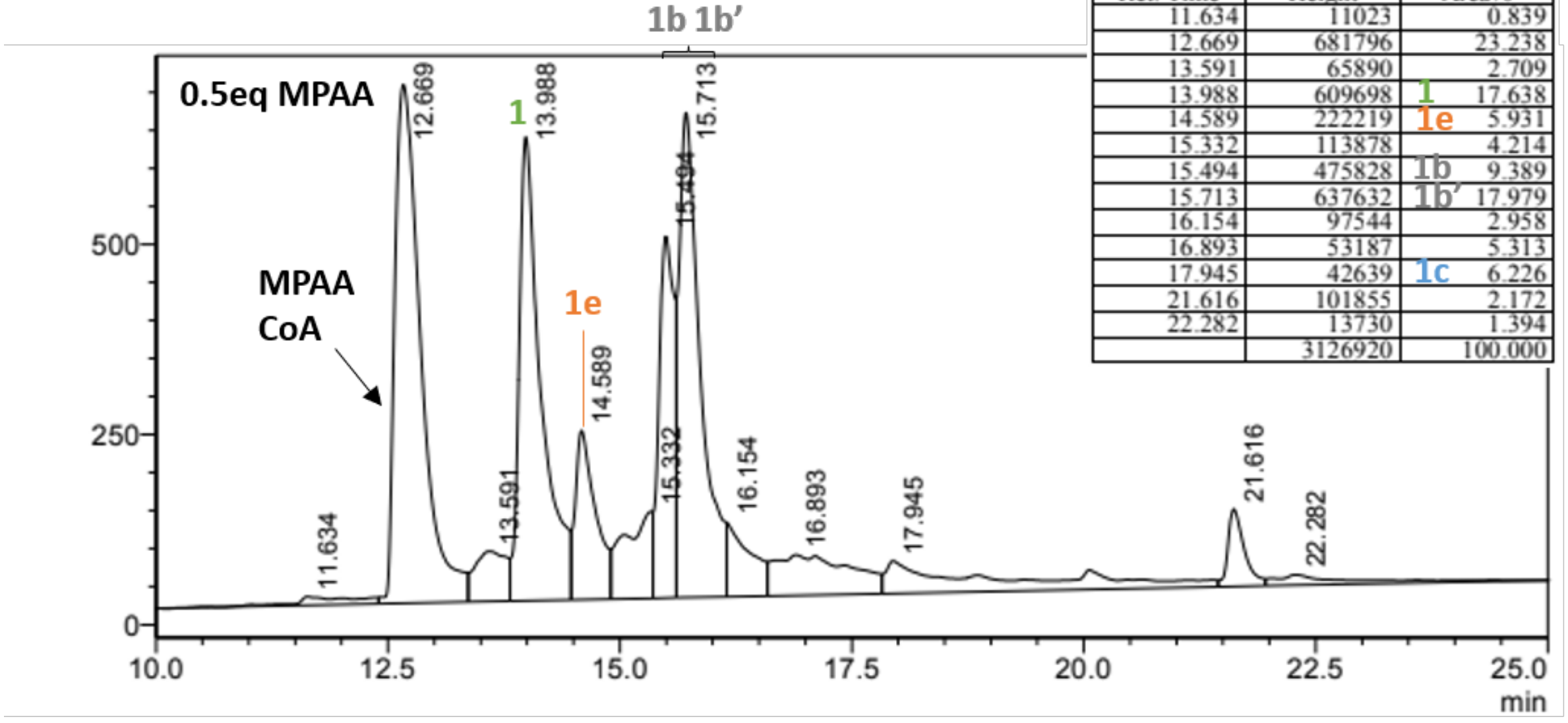

Detector A Channel 1 214nm

Ret. Time	Height		Area%
11.634	11023		0.839
12.669	681796		23.238
13.591	65890		2.709
13.988	609698	1	17.638
14.589	222219	1e	5.931
15.332	113878		4.214
15.494	475828	1b	9.389
15.713	637632	1b'	17.979
16.154	97544		2.958
16.893	53187		5.313
17.945	42639	1c	6.226
21.616	101855		2.172
22.282	13730		1.394
	3126920		100.000

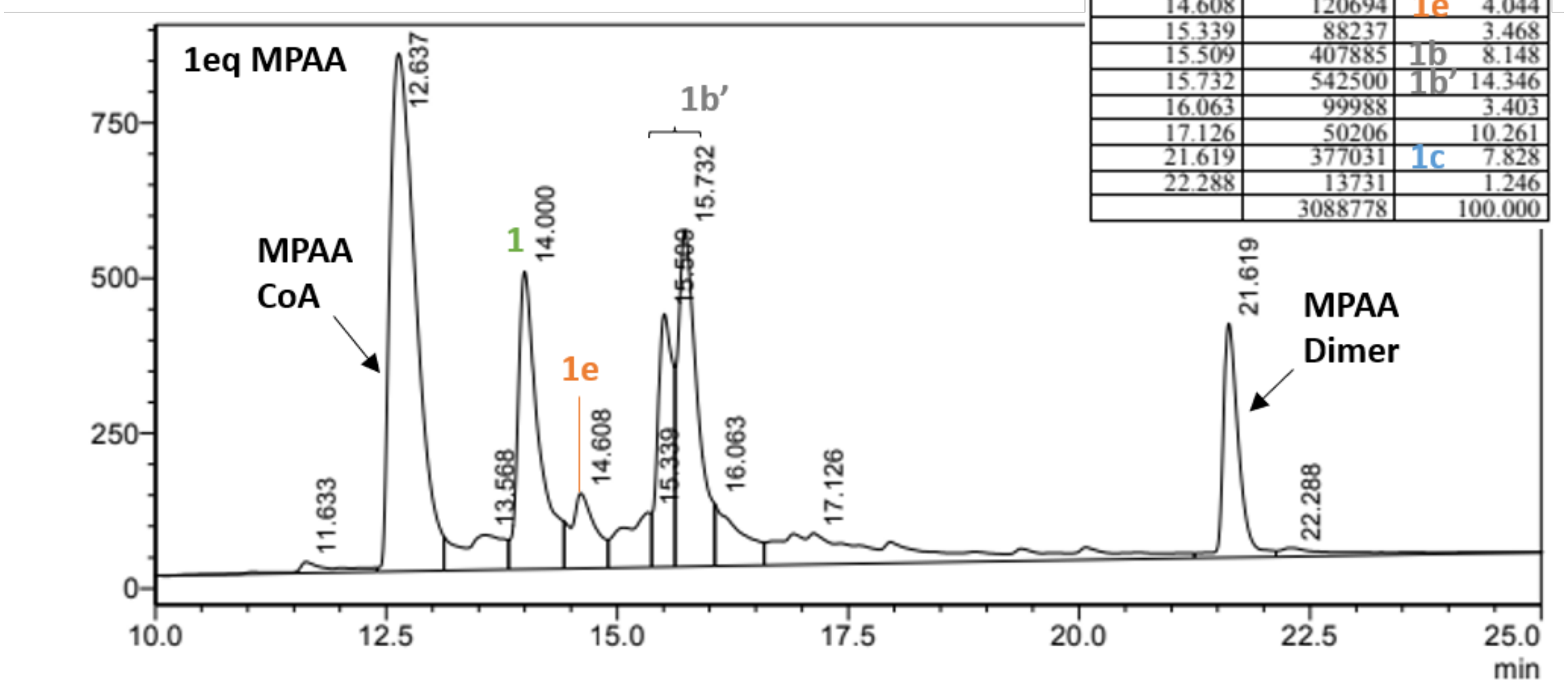

Detector A Channel 1 214nm

Ret. Time	Height		Area%
11.633	17754		0.931
12.637	834029		29.342
13.568	56627		3.558
14.000	480096	1	13.425
14.608	120694	1e	4.044
15.339	88237		3.468
15.509	407885	1b	8.148
15.732	542500	1b'	14.346
16.063	99988		3.403
17.126	50206		10.261
21.619	377031	1c	7.828
22.288	13731		1.246
	3088778		100.000

SI Figure 5. LC chromatogram and table data linked to **Figure 2A** (entries 0.5 eq and 1 eq of MPAA).

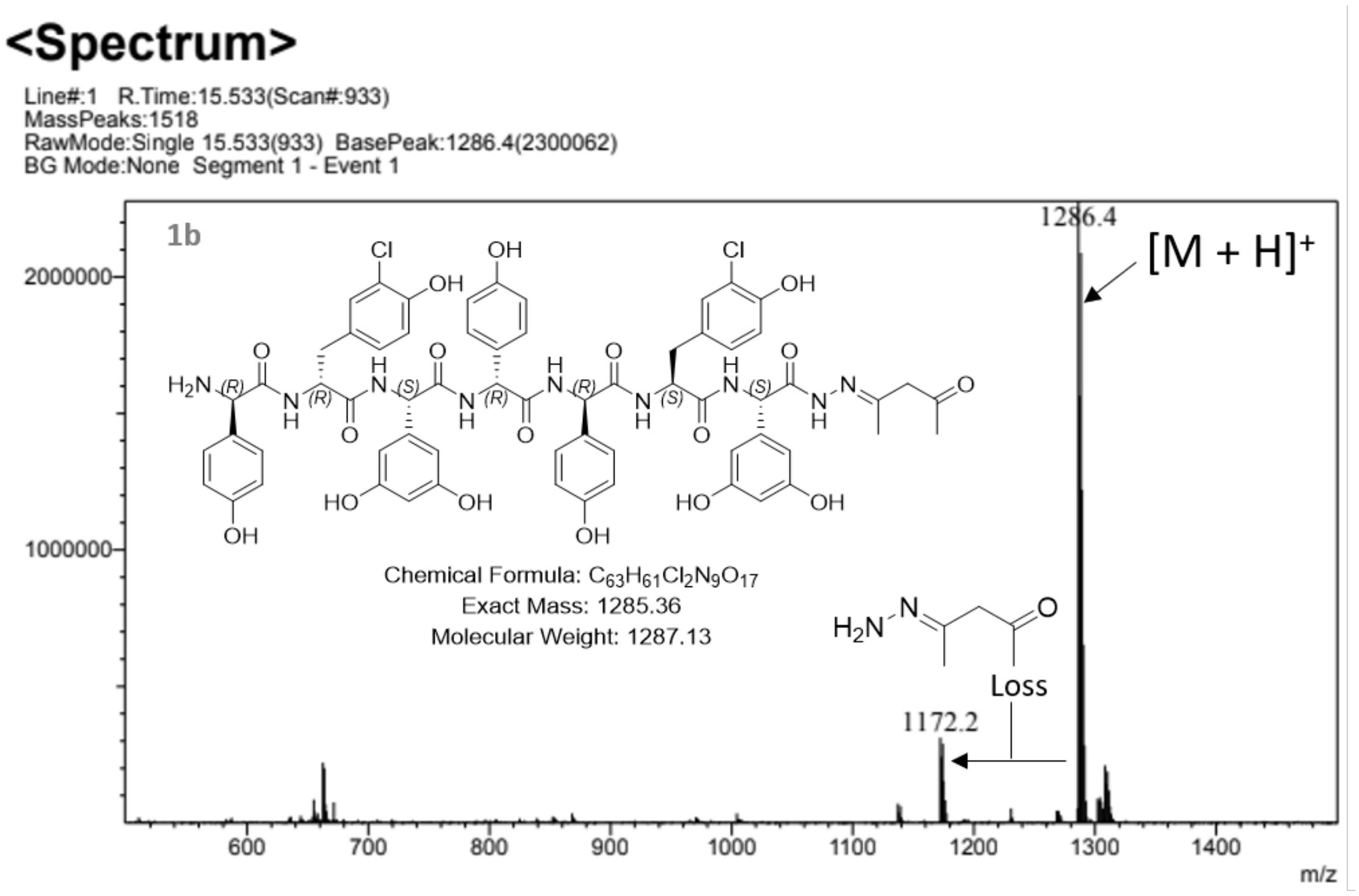

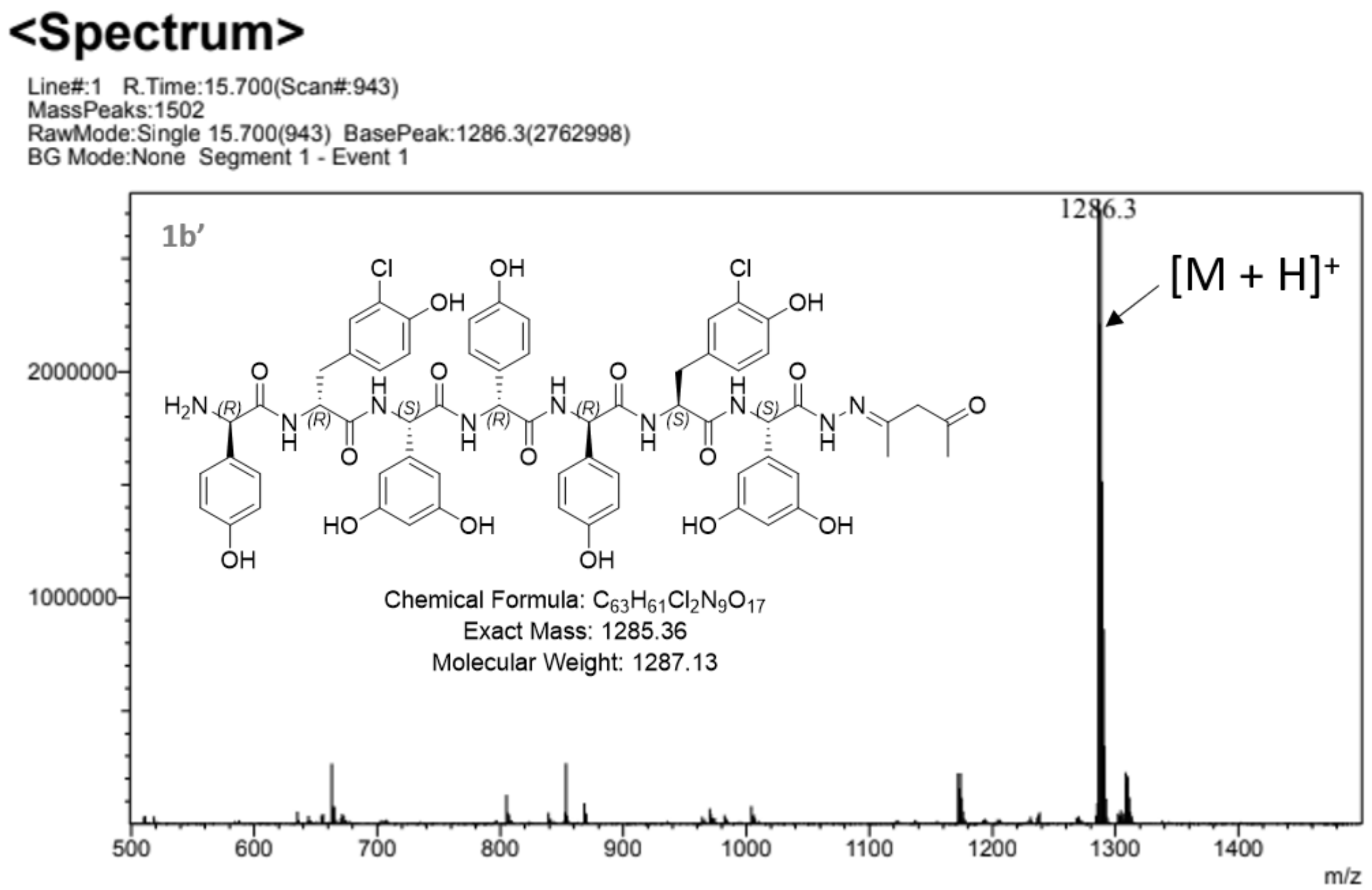

SI Figure 6. Mass spectrometry chromatograms of the peptidyl-hydrazones **1b** and **1b’.**

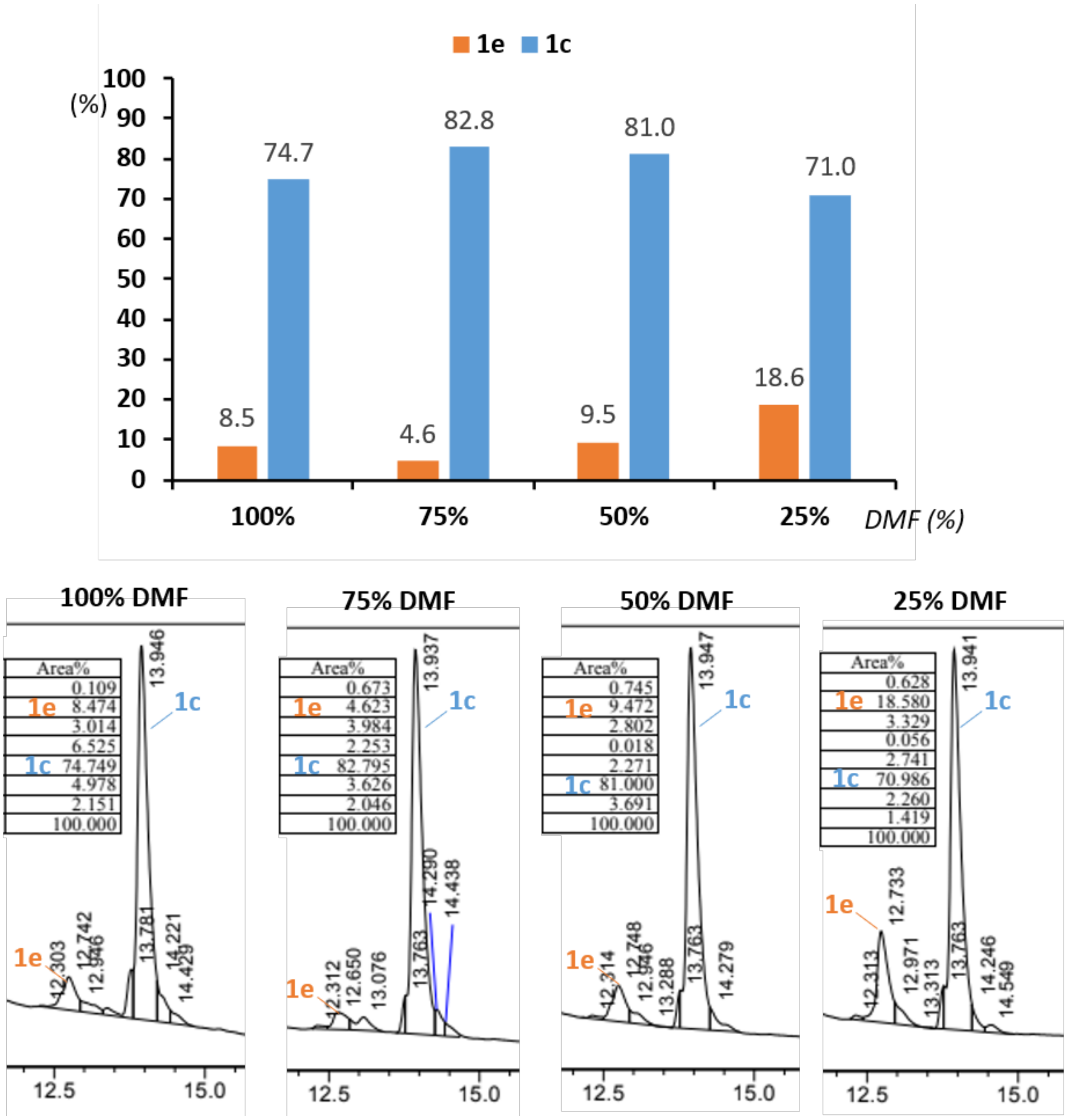

SI Figure 7. Formation of the peptidyl-pyrazole (**1c**) in aqueous-DMF mixtures and the peptide hydrolysis (**1e**). Top. A plot showing the percentage of **1c** and **1e**. Bottom. A snapshot of the LC chromatogram and table data.

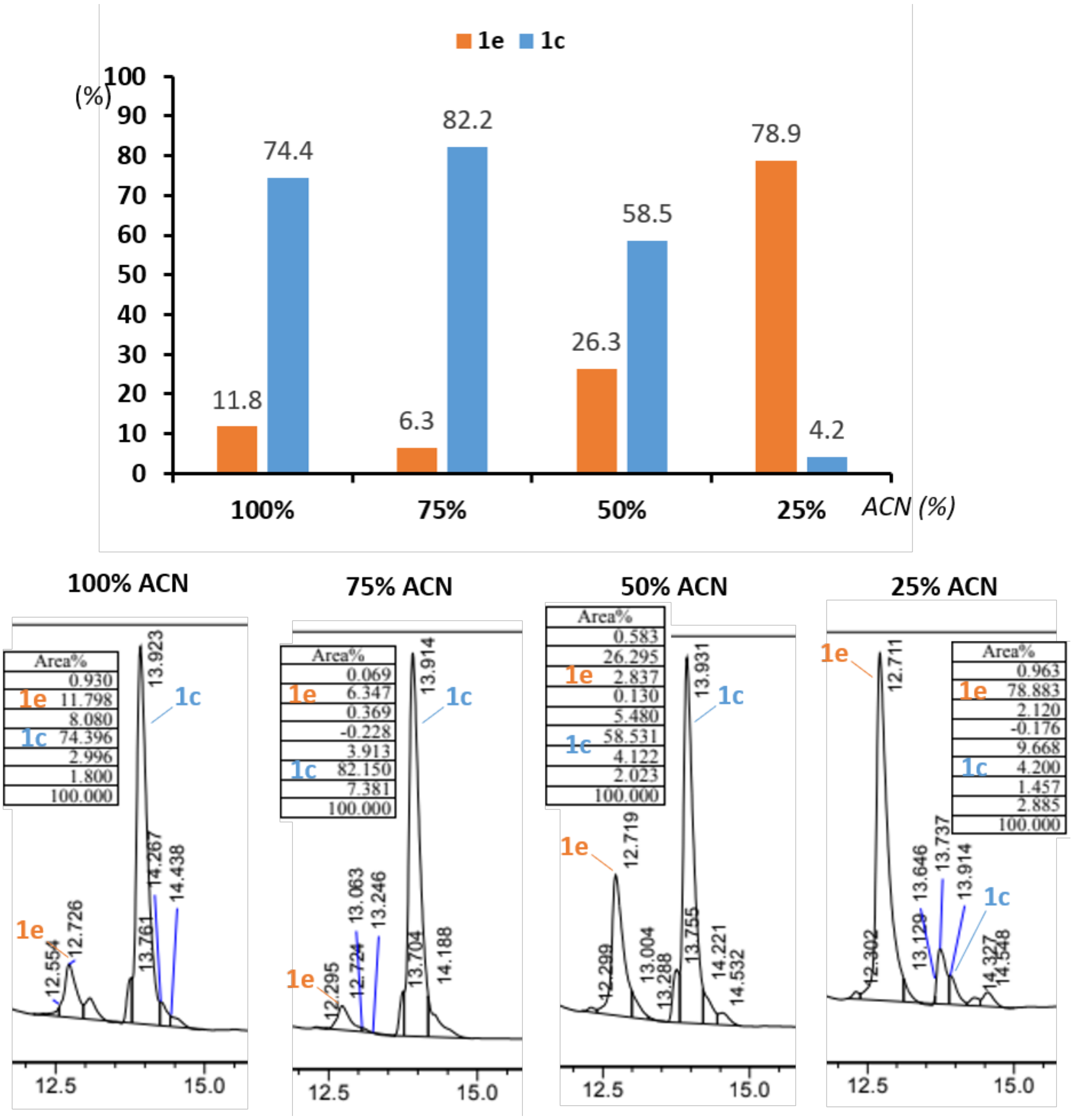

SI Figure 8. Formation of the peptidyl-pyrazole (**1c**) in aqueous-ACN mixtures and the peptide hydrolysis (**1e**). Top. A plot showing the percentage of **1c** and **1e**. Bottom. A snapshot of the LC chromatogram and table data.

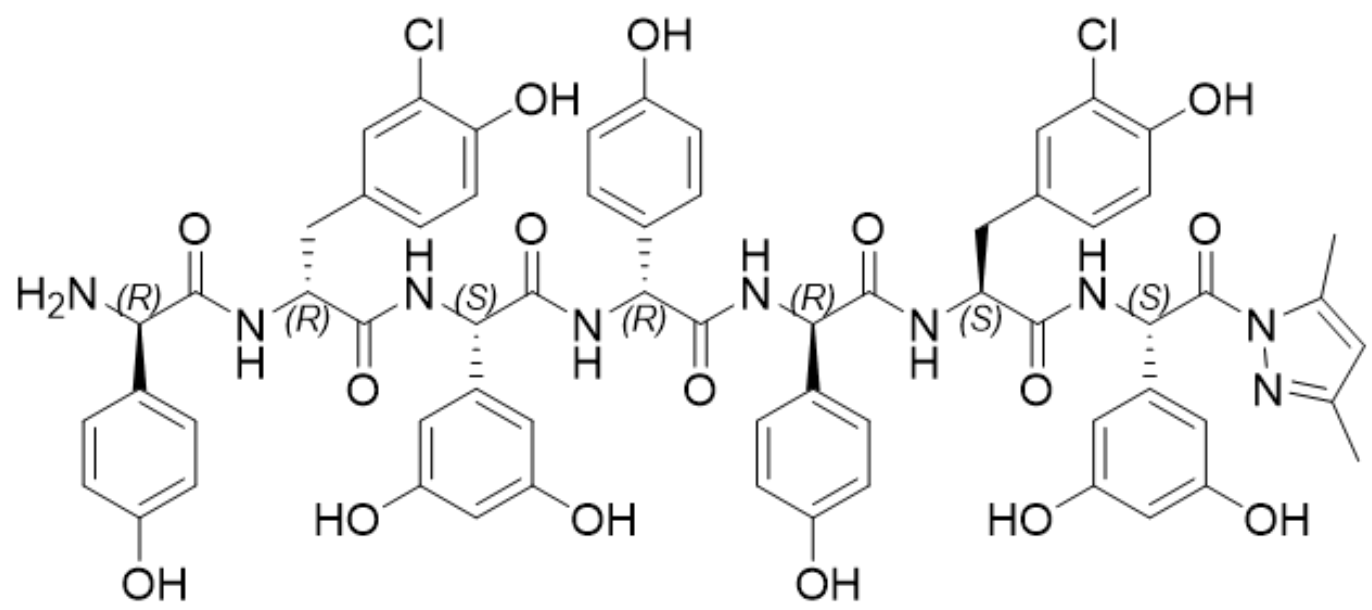

1c

Chemical Formula: $C_{63}H_{59}Cl_2N_9O_{16}$
Exact Mass: 1267.35
Molecular Weight: 1269.11

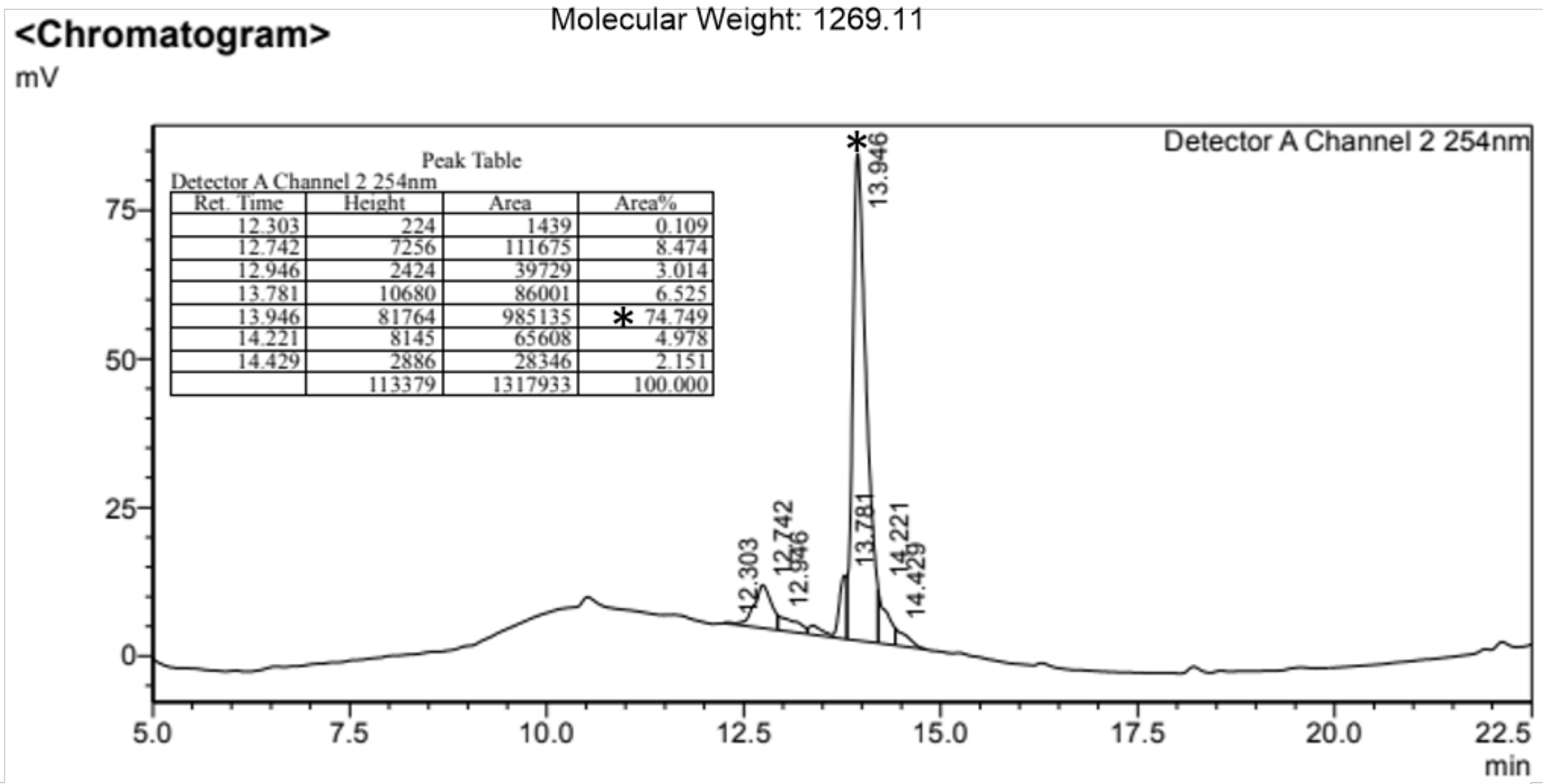

Ret. Time	Height	Area	Area%
12.303	224	1439	0.109
12.742	7256	111675	8.474
12.946	2424	39729	3.014
13.781	10680	86001	6.525
13.946	81764	985135	* 74.749
14.221	8145	65608	4.978
14.429	2886	28346	2.151
	113379	1317933	100.000

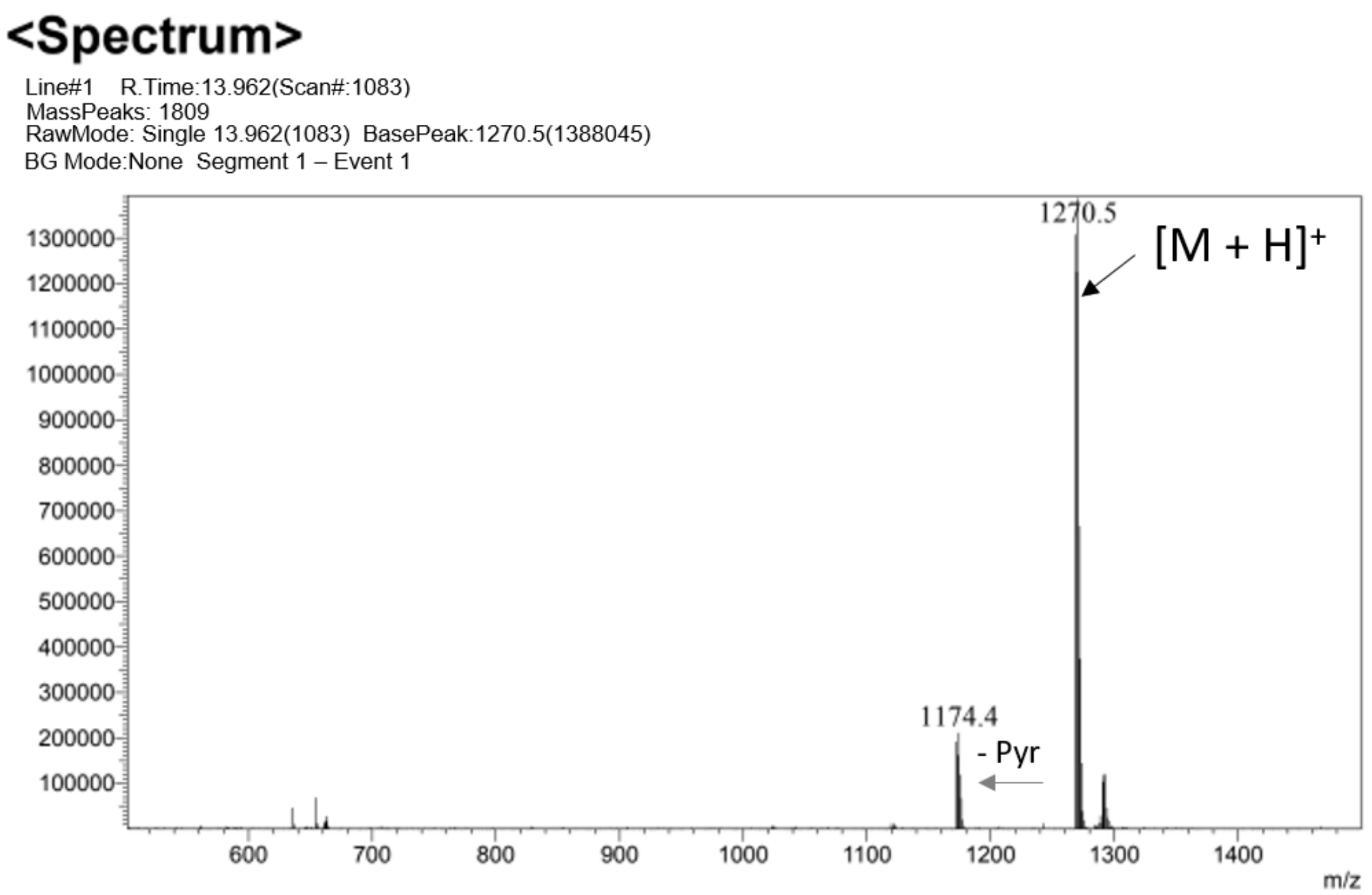

SI Figure 9. LCMS chromatograms of the formation of **1c** in DMF at pH 3. The loss of the pyrazole is indicated as (-Pyr).

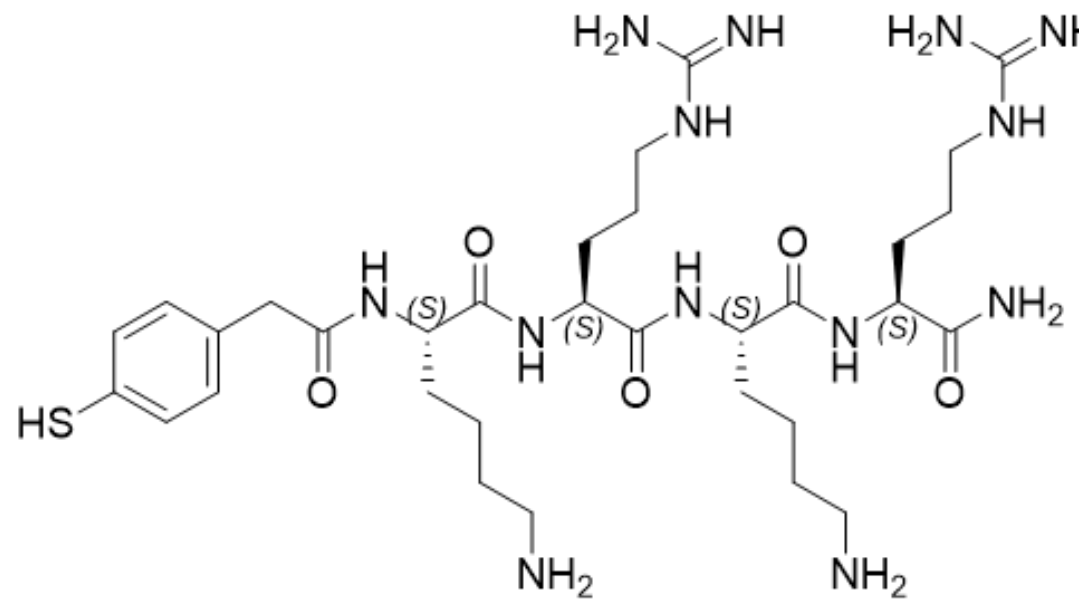

4

Chemical Formula: $C_{32}H_{57}N_{13}O_5S$
Exact Mass: 735.43
Molecular Weight: 735.95

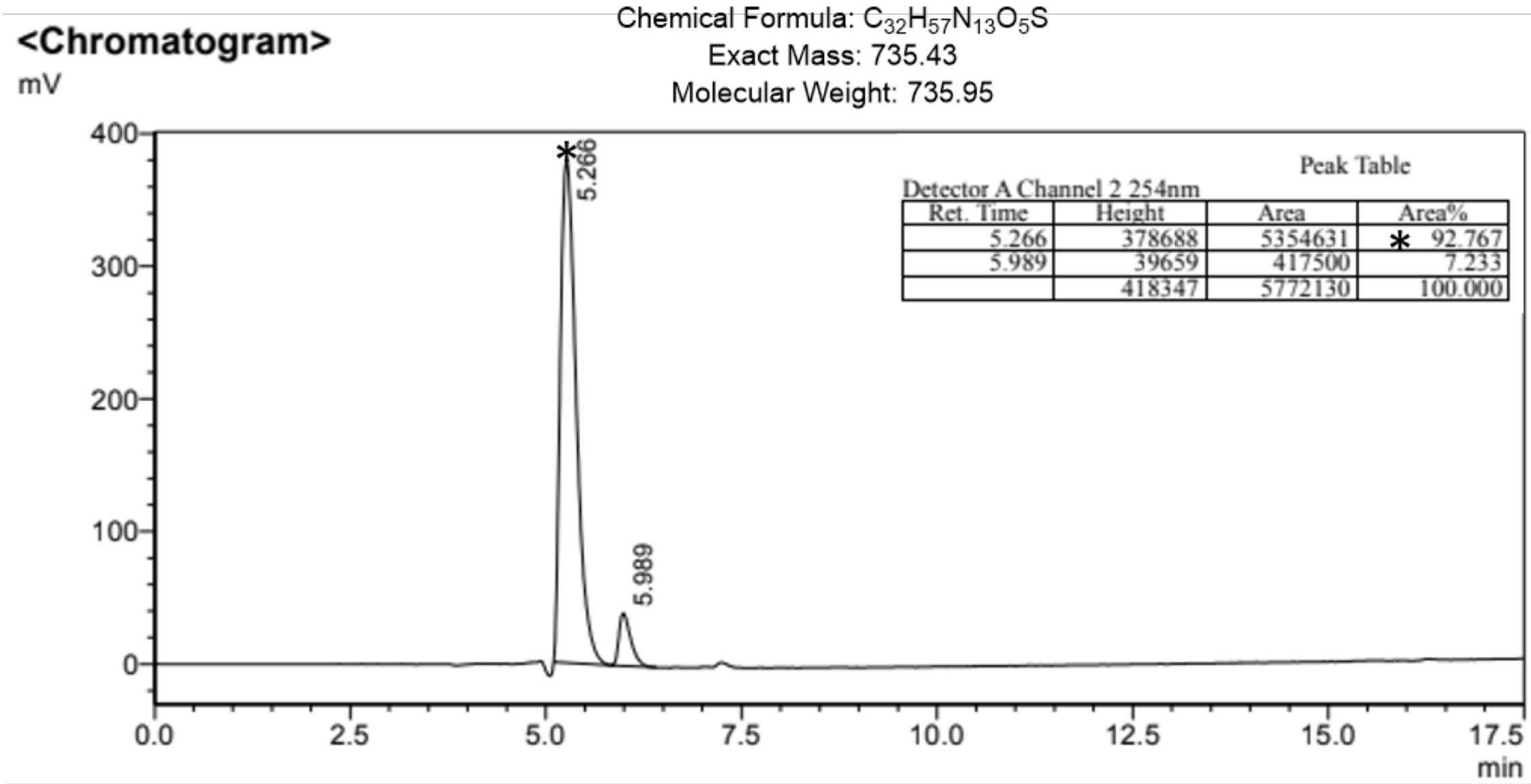

Ret. Time	Height	Area	Area%
5.266	378688	5354631	* 92.767
5.989	39659	417500	7.233
	418347	5772130	100.000

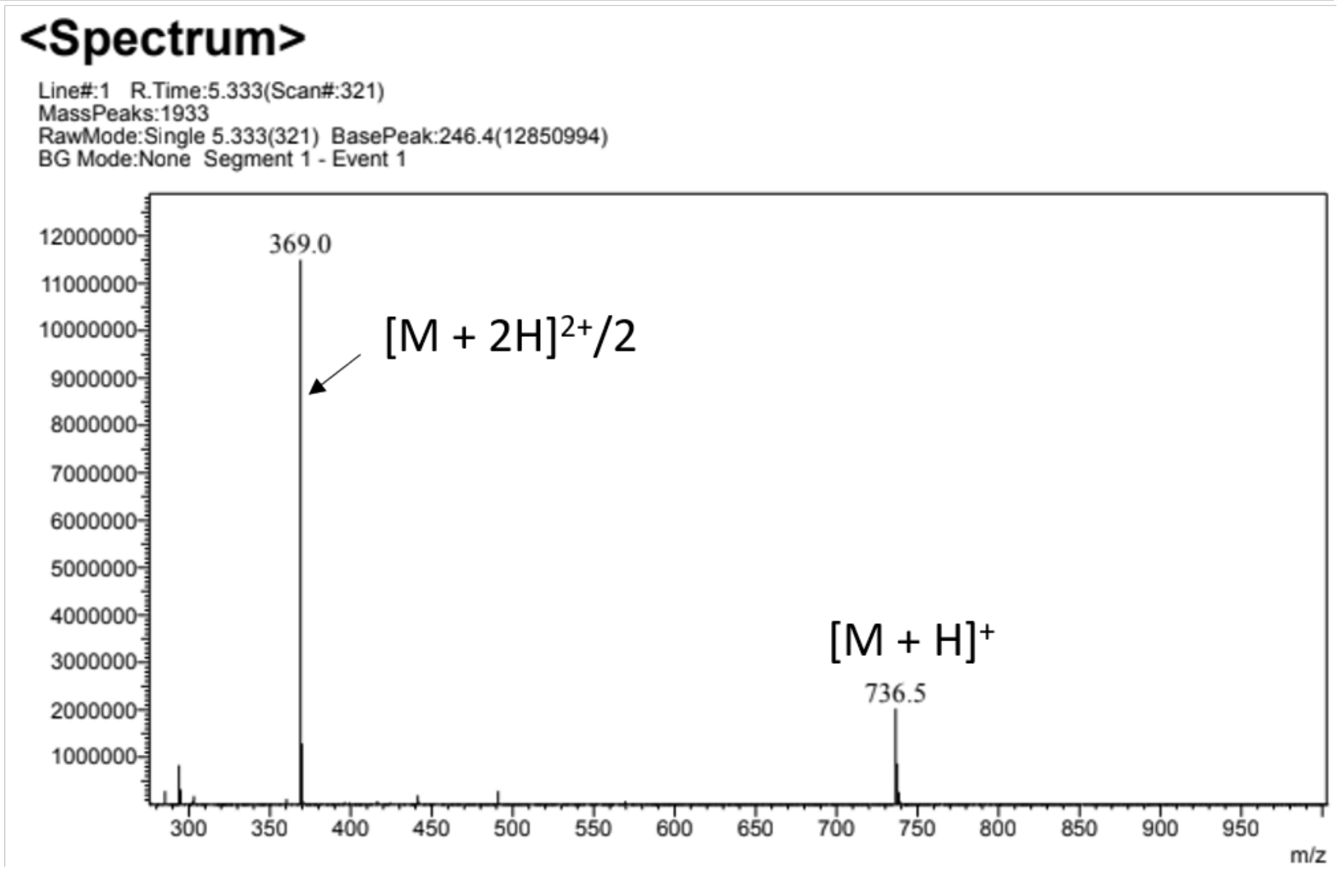

SI Figure 10. LCMS chromatograms of **4**.

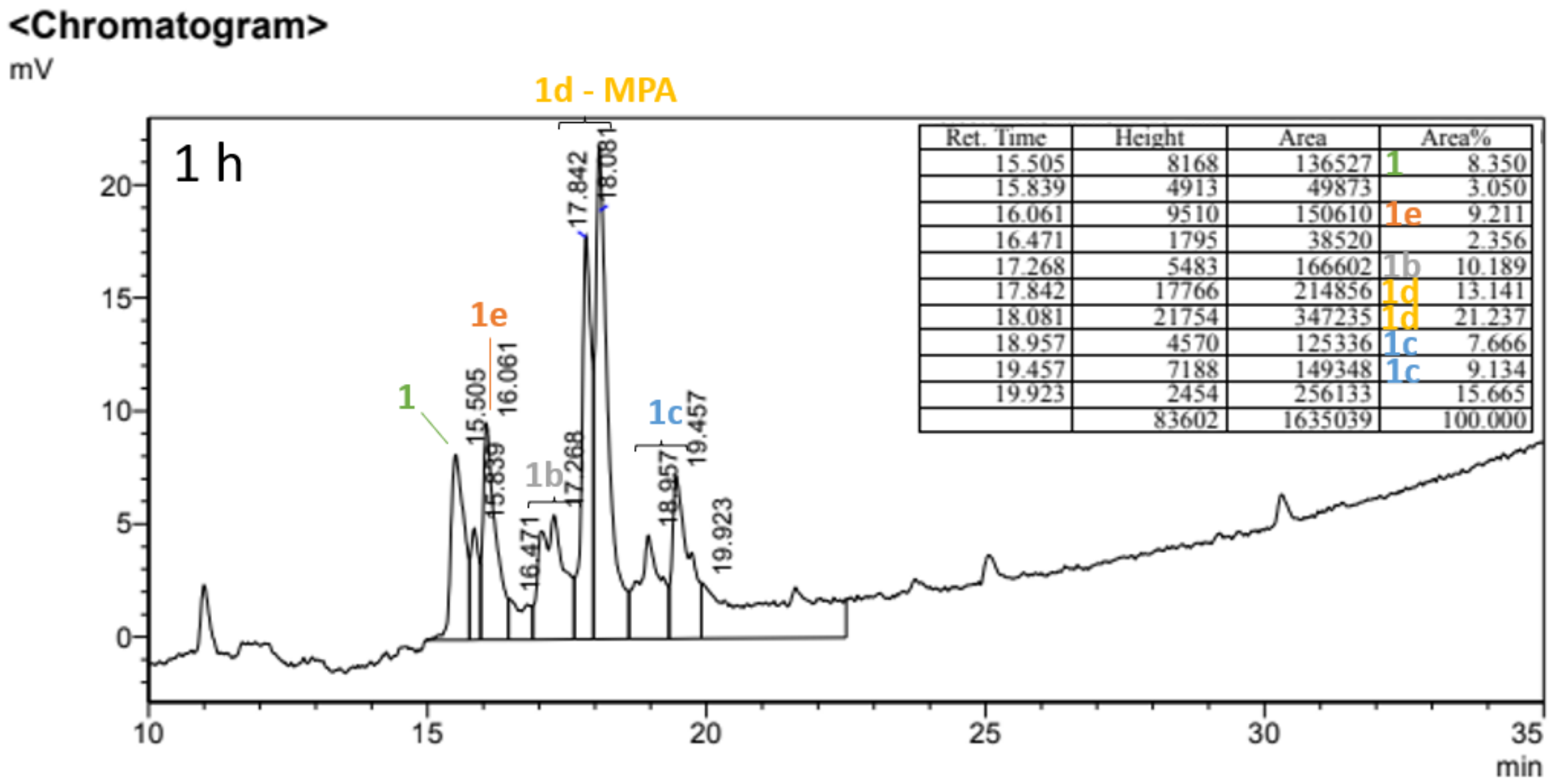

Ret. Time	Height	Area		Area%
15.505	8168	136527	1	8.350
15.839	4913	49873		3.050
16.061	9510	150610	1e	9.211
16.471	1795	38520		2.356
17.268	5483	166602	1b	10.189
17.842	17766	214856	1d	13.141
18.081	21754	347235	1d	21.237
18.957	4570	125336	1c	7.666
19.457	7188	149348	1c	9.134
19.923	2454	256133		15.665
	83602	1635039		100.000

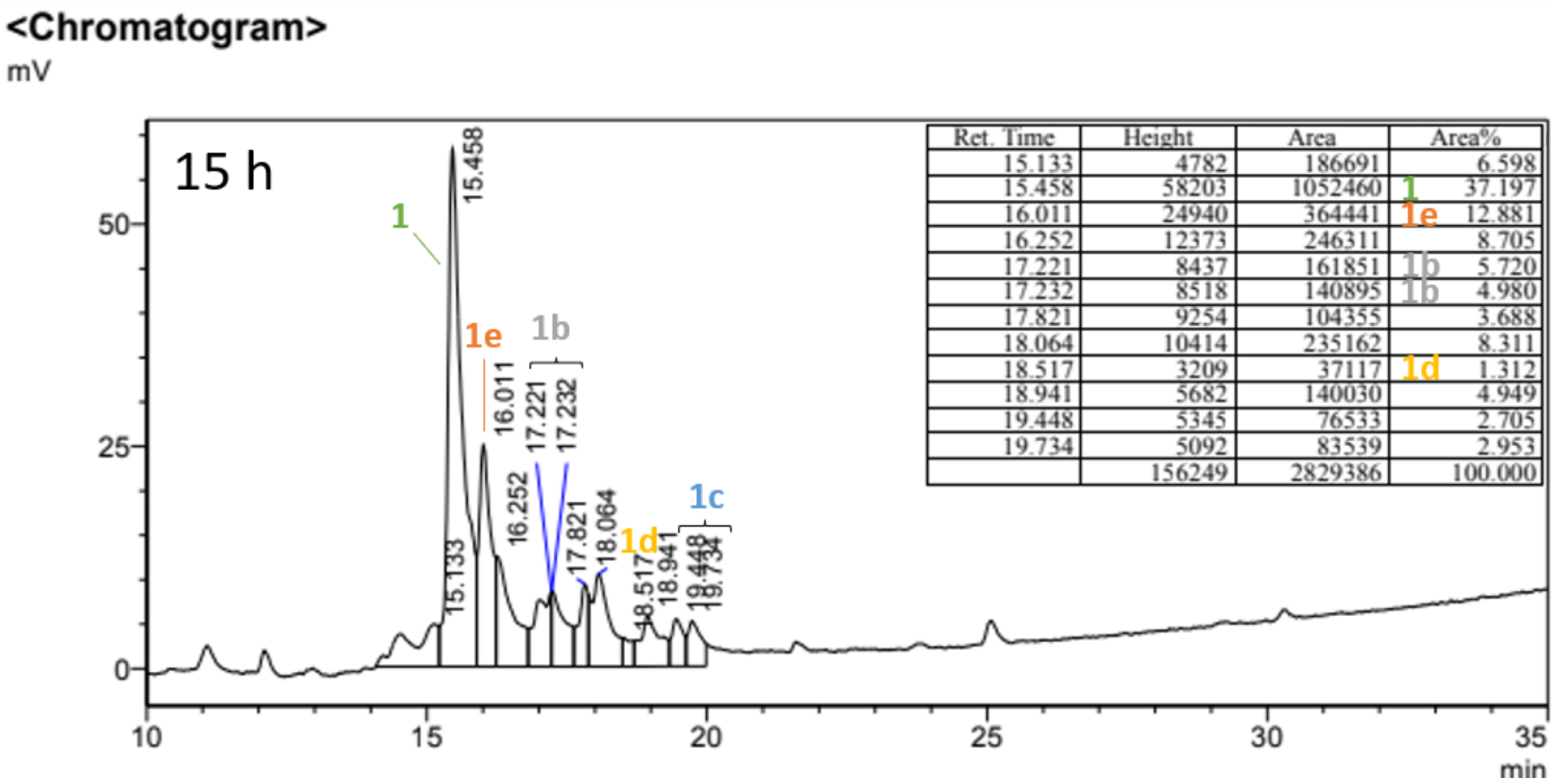

Ret. Time	Height	Area		Area%
15.133	4782	186691		6.598
15.458	58203	1052460	1	37.197
16.011	24940	364441	1e	12.881
16.252	12373	246311		8.705
17.221	8437	161851	1b	5.720
17.232	8518	140895	1b	4.980
17.821	9254	104355		3.688
18.064	10414	235162		8.311
18.517	3209	37117	1d	1.312
18.941	5682	140030		4.949
19.448	5345	76533		2.705
19.734	5092	83539		2.953
	156249	2829386		100.000

SI Figure 11. LC chromatogram monitoring the reaction at 1 h (Top) and 15 h (Bottom) and table data before normalisation in the link to **Figure 3** (entry MPA – 4-aminomercaptophenol).

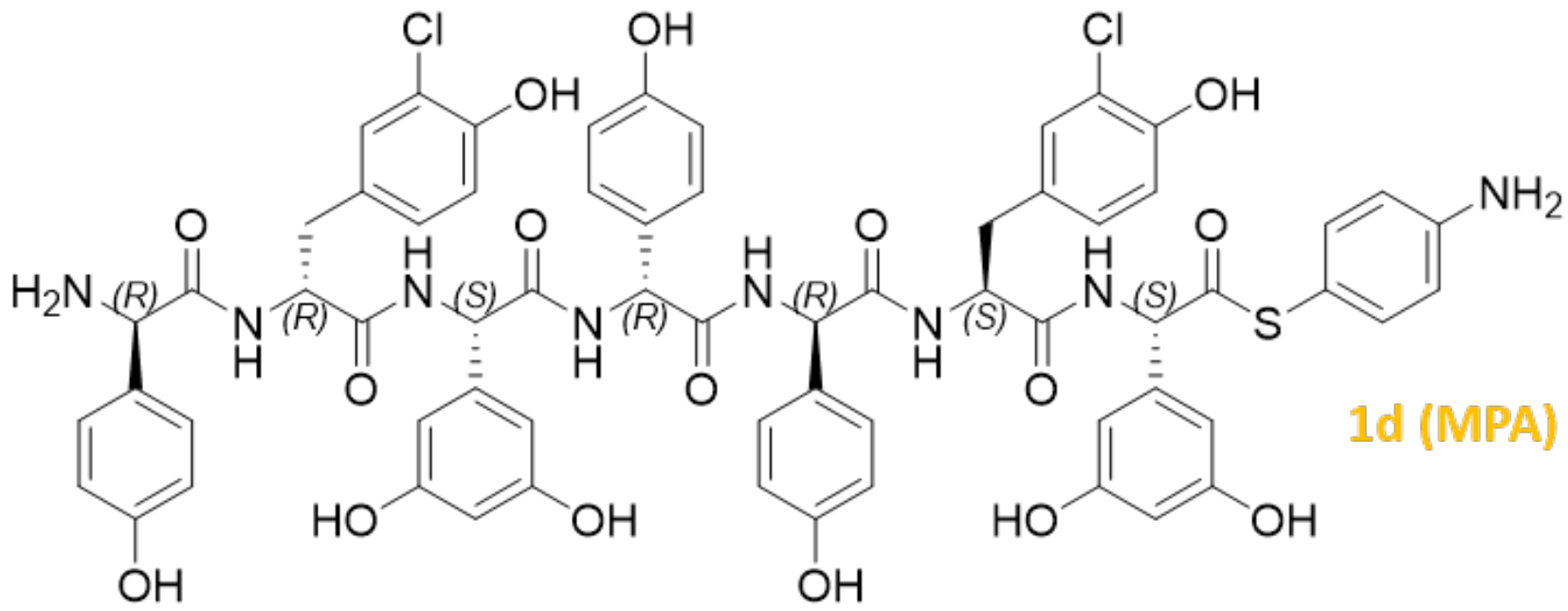

1d (MPA)

Chemical Formula: $C_{64}H_{58}Cl_2N_8O_{16}S$
Exact Mass: 1296.31
Molecular Weight: 1298.17

<Spectrum>

Line#:1 R.Time:18.133(Scan#:1089)
MassPeaks:1953
RawMode:Single 18.133(1089) BasePeak:650.1(1132064)
BG Mode:None Segment 1 - Event 1

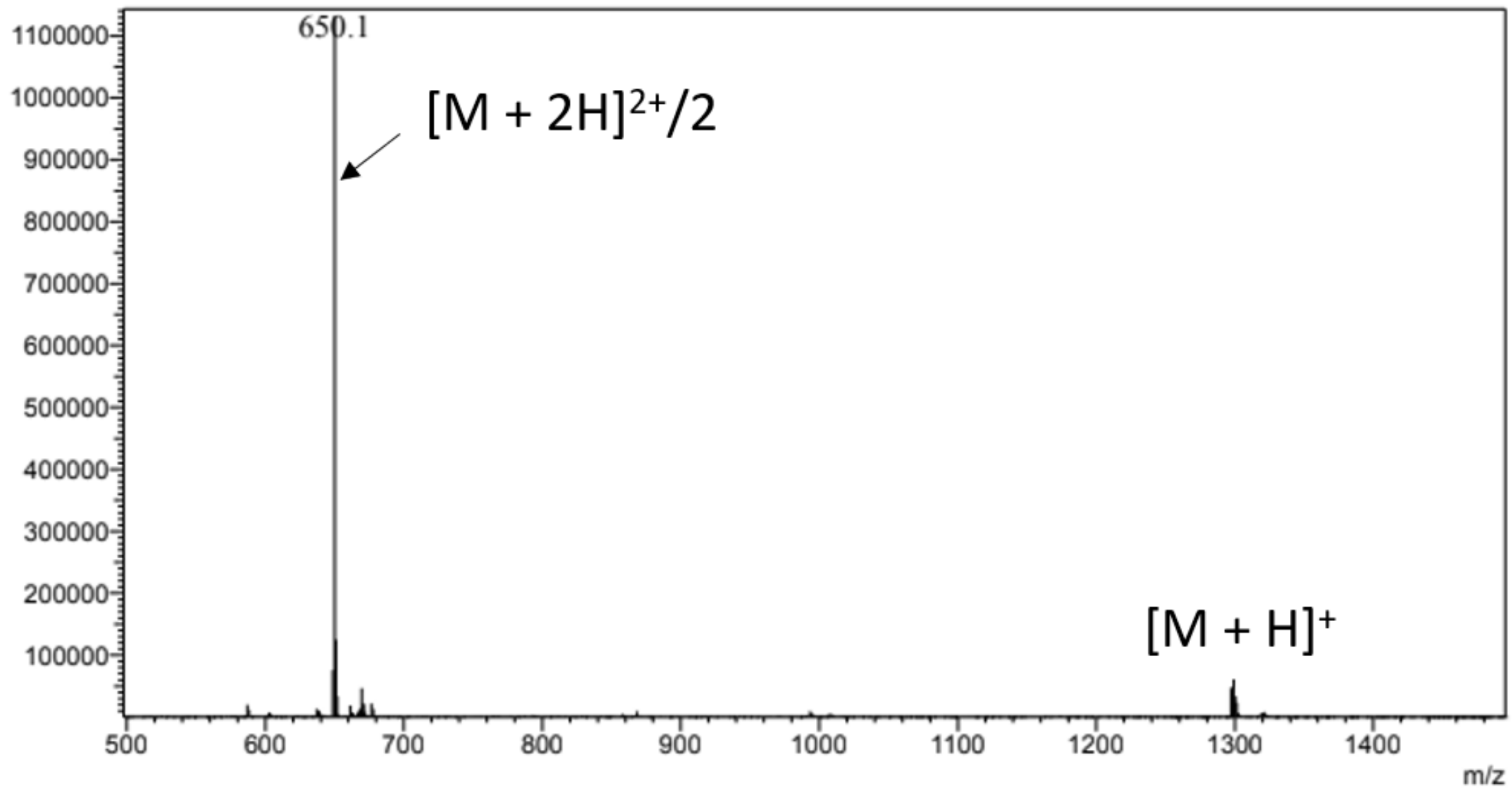

SI Figure 12. Mass spectrum of the peptidyl-thioester **1d - MPA.**

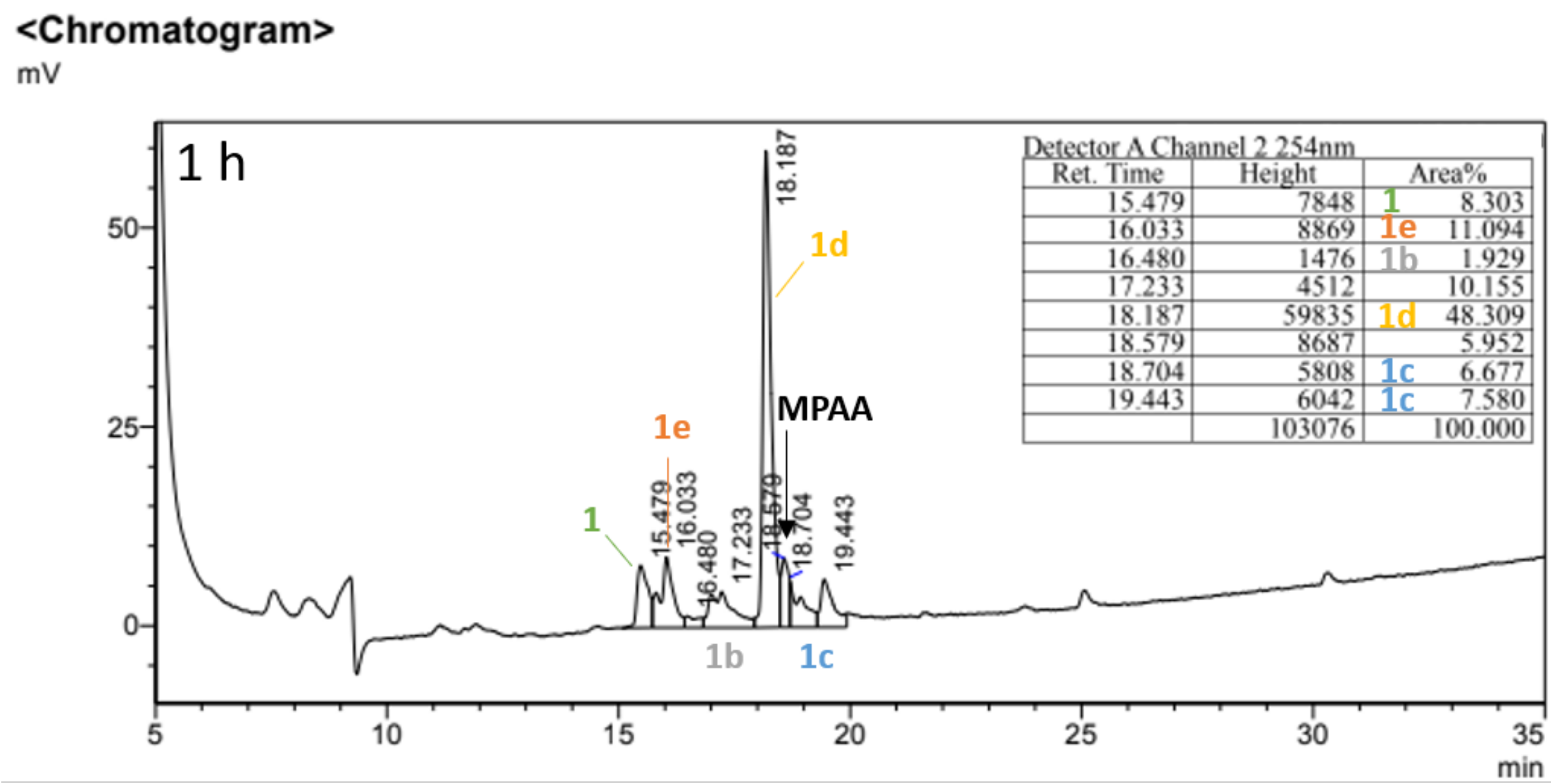

Detector A Channel 2 254nm

Ret. Time	Height	Area%
15.479	7848	1 8.303
16.033	8869	1e 11.094
16.480	1476	1b 1.929
17.233	4512	10.155
18.187	59835	1d 48.309
18.579	8687	5.952
18.704	5808	1c 6.677
19.443	6042	1c 7.580
	103076	100.000

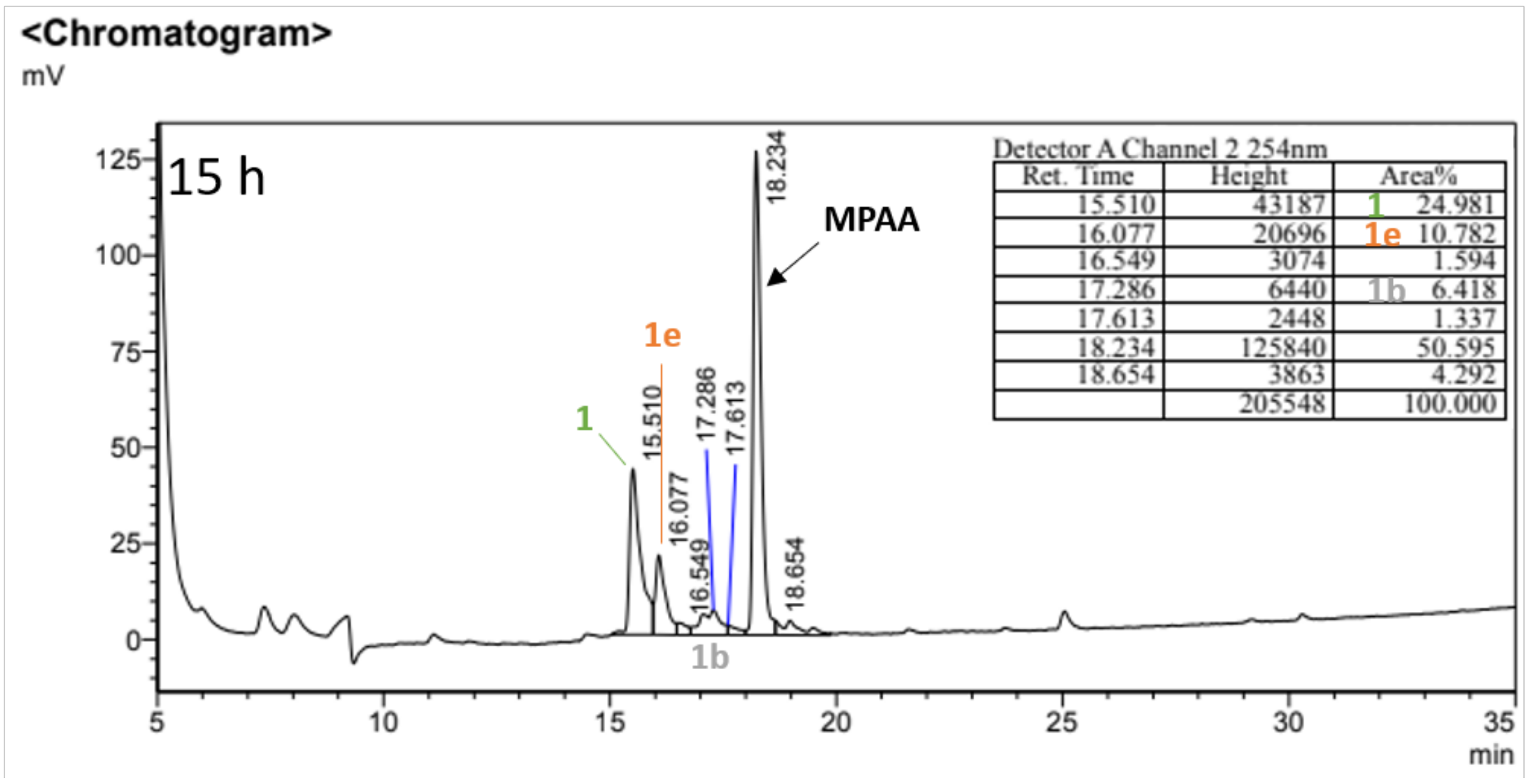

Detector A Channel 2 254nm

Ret. Time	Height	Area%
15.510	43187	1 24.981
16.077	20696	1e 10.782
16.549	3074	1.594
17.286	6440	1b 6.418
17.613	2448	1.337
18.234	125840	50.595
18.654	3863	4.292
	205548	100.000

SI Figure 13. LC chromatogram monitoring the reaction at 1 h (Top) and 15 h (Bottom) and table data before normalisation in the link to **Figure 3** (entry MPAA – 4-mercaptophenylacetic acid).

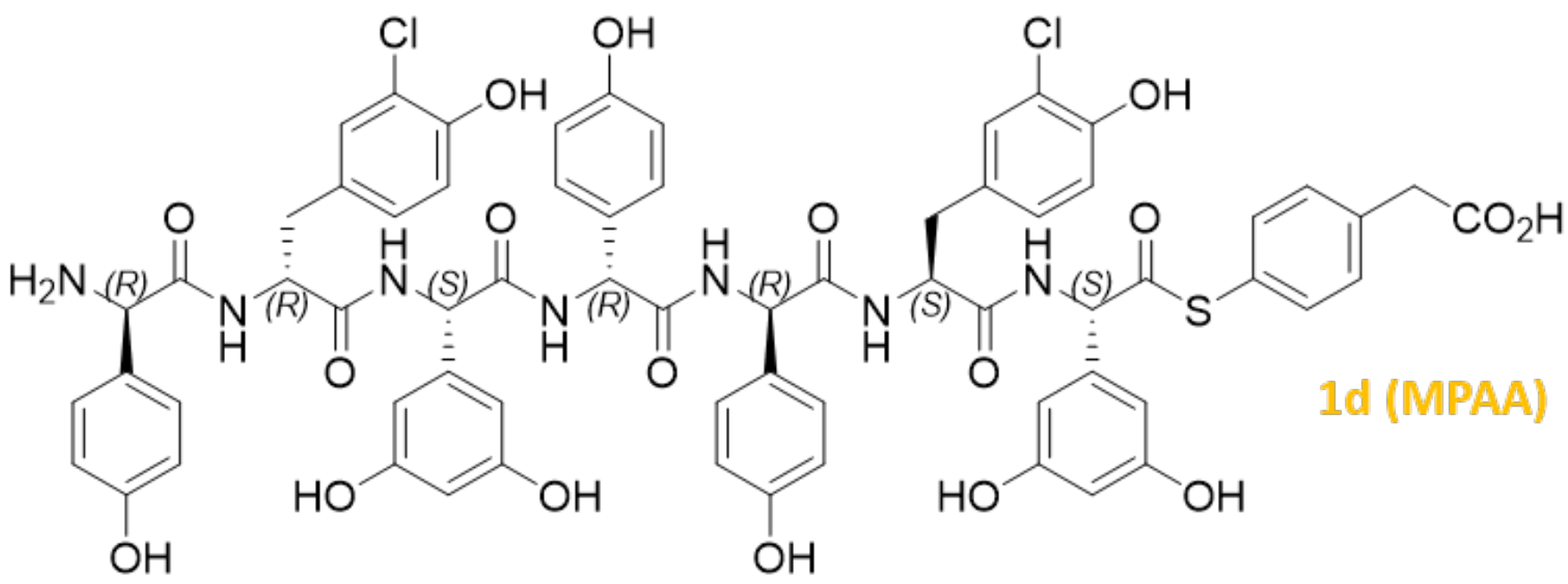

Chemical Formula: $C_{66}H_{59}Cl_2N_7O_{18}S$
Exact Mass: 1339.30
Molecular Weight: 1341.19

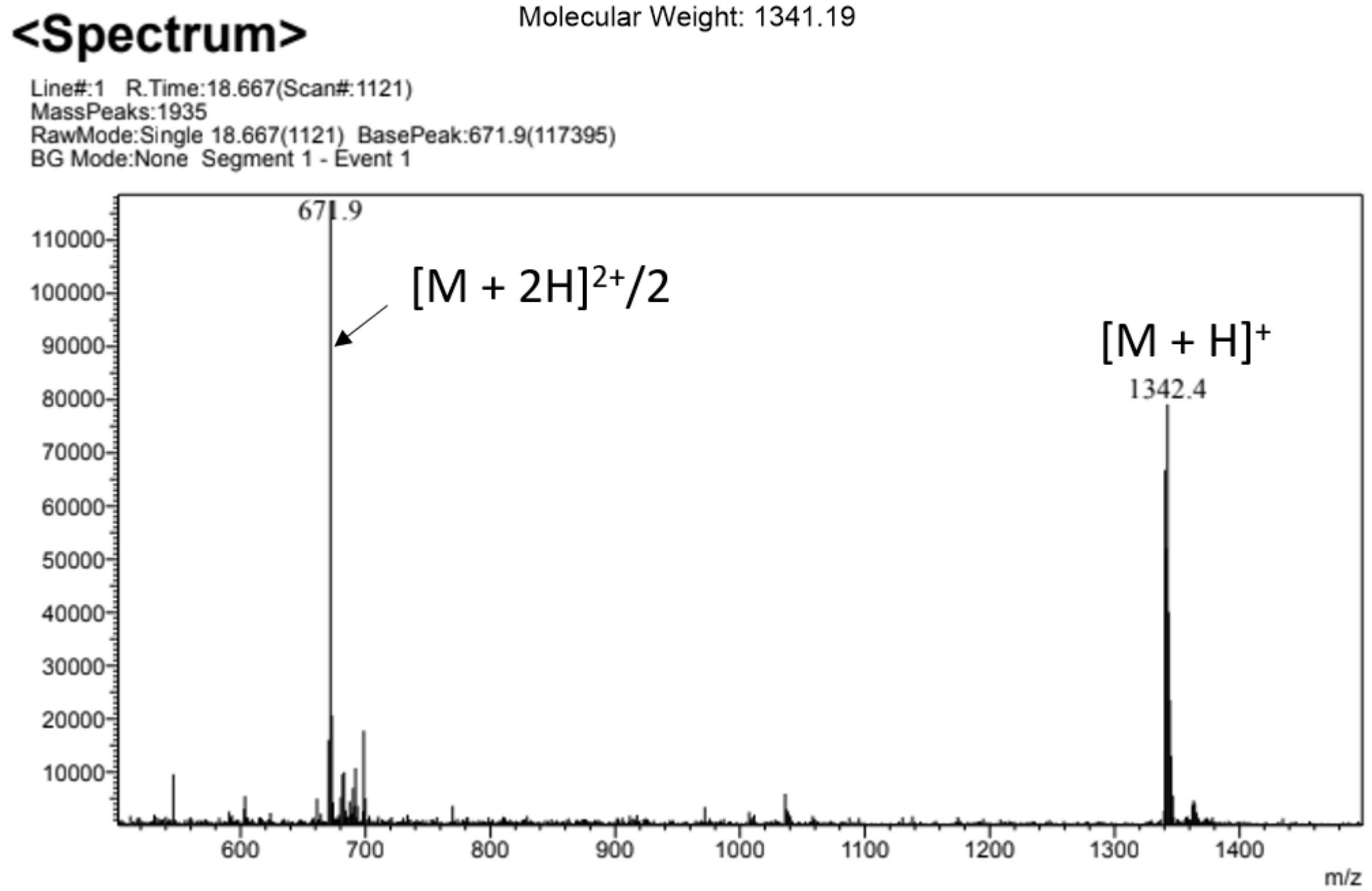

SI Figure 14. Mass spectrum of the peptidyl-thioester **1d.**

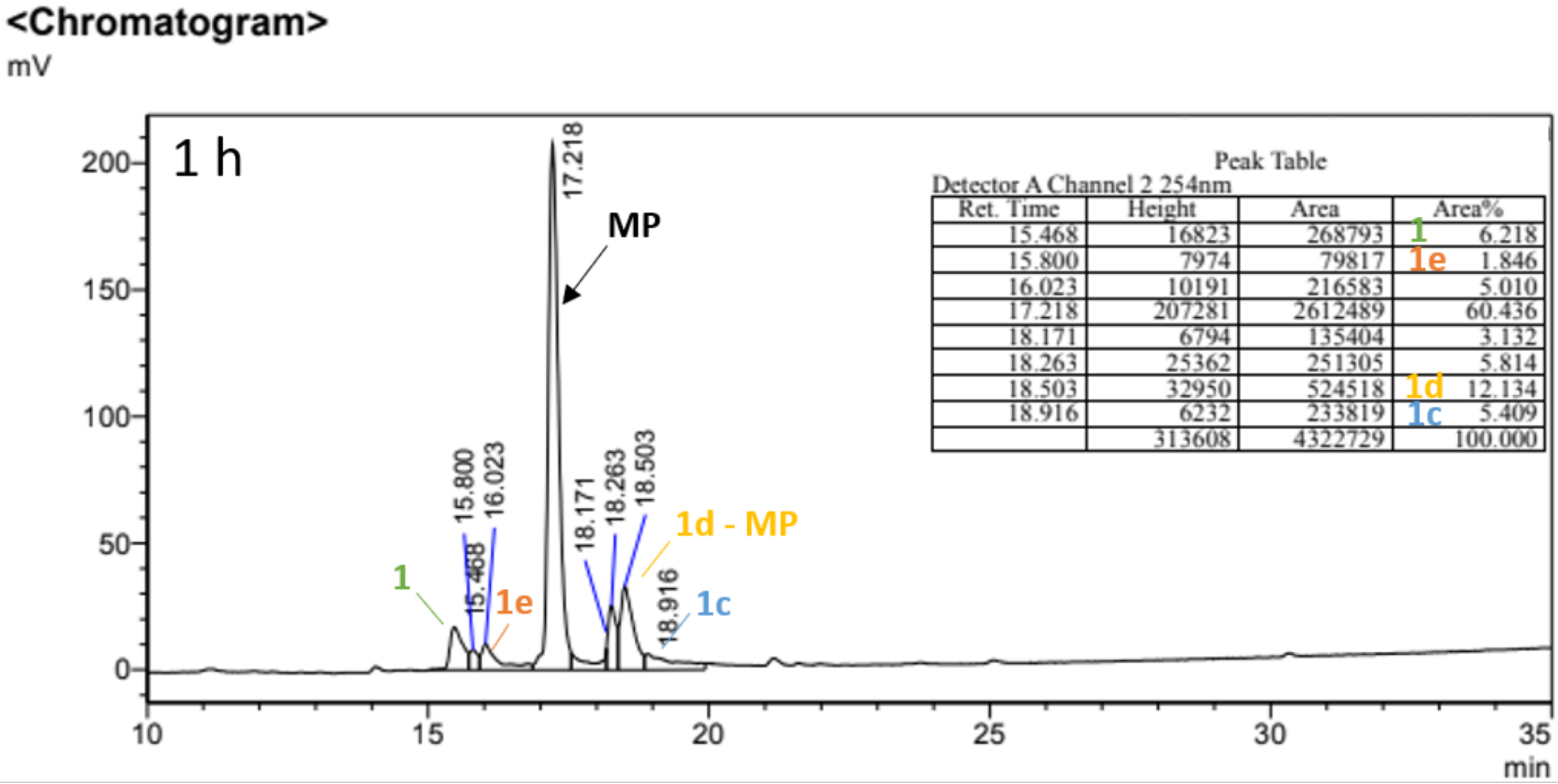

Peak Table
Detector A Channel 2 254nm

Ret. Time	Height	Area	Area%
15.468	16823	268793	1 6.218
15.800	7974	79817	1e 1.846
16.023	10191	216583	5.010
17.218	207281	2612489	60.436
18.171	6794	135404	3.132
18.263	25362	251305	5.814
18.503	32950	524518	1d 12.134
18.916	6232	233819	1c 5.409
	313608	4322729	100.000

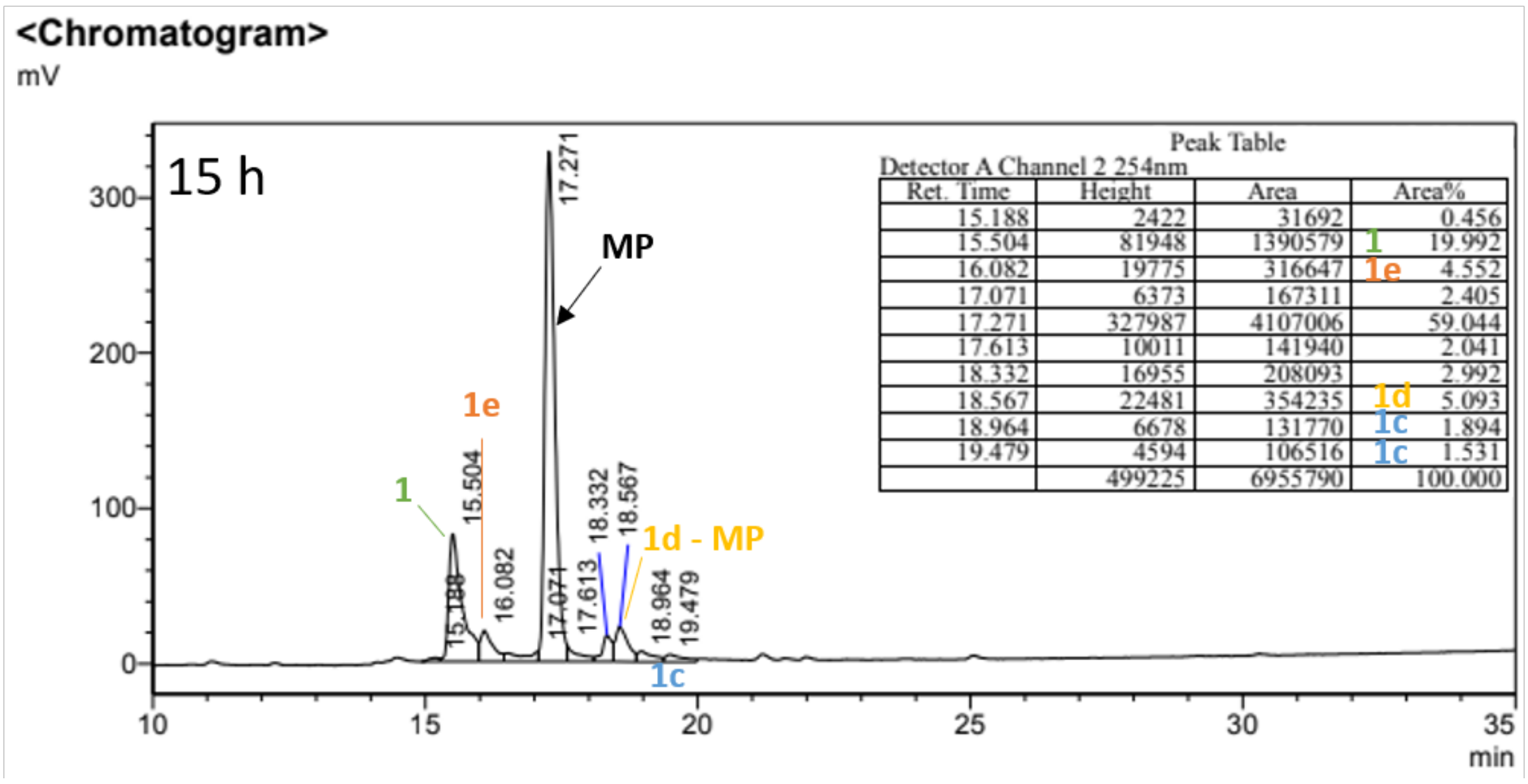

Peak Table
Detector A Channel 2 254nm

Ret. Time	Height	Area	Area%
15.188	2422	31692	0.456
15.504	81948	1390579	1 19.992
16.082	19775	316647	1e 4.552
17.071	6373	167311	2.405
17.271	327987	4107006	59.044
17.613	10011	141940	2.041
18.332	16955	208093	2.992
18.567	22481	354235	1d 5.093
18.964	6678	131770	1c 1.894
19.479	4594	106516	1c 1.531
	499225	6955790	100.000

SI Figure 15. LC chromatogram monitoring the reaction at 1 h (Top) and 15 h (Bottom) and table data before normalisation in the link to **Figure 3** (entry MP – 4-mercaptophenol).

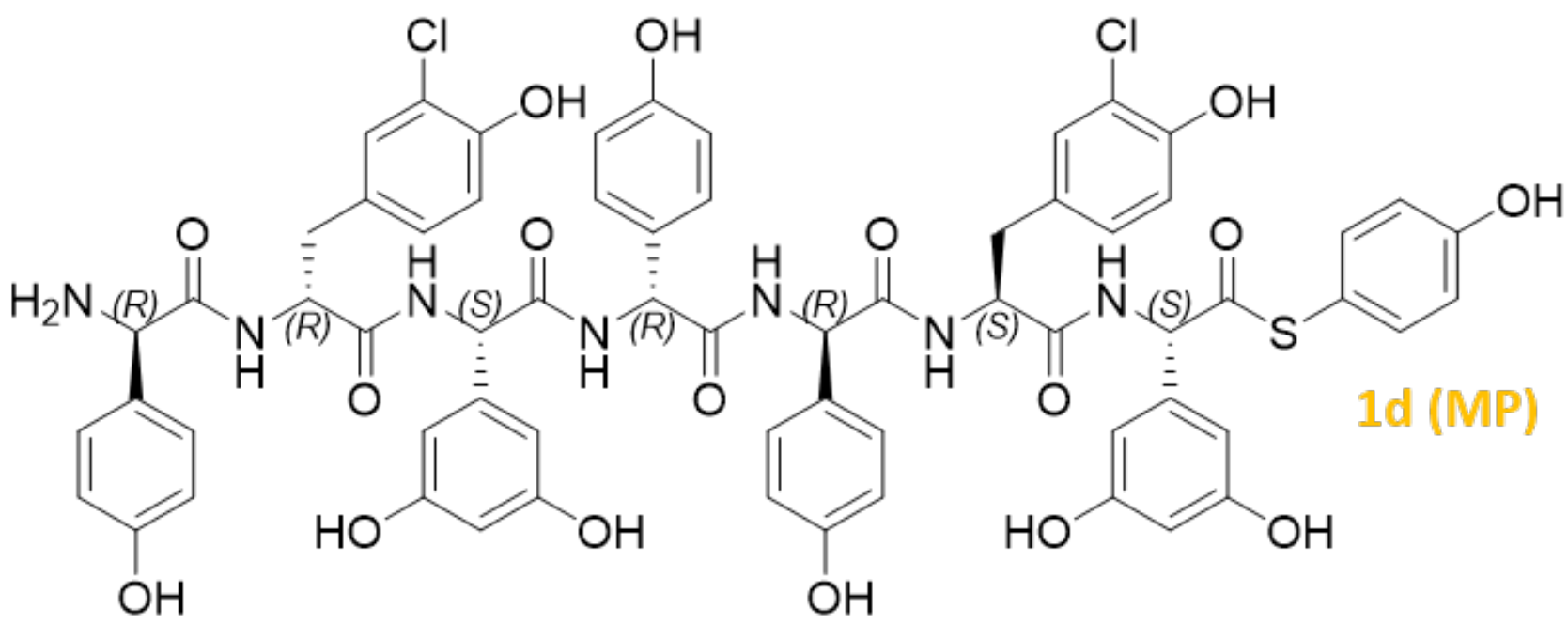

1d (MP)

Chemical Formula: $C_{64}H_{57}Cl_2N_7O_{17}S$
Exact Mass: 1297.29
Molecular Weight: 1299.15

<Spectrum>

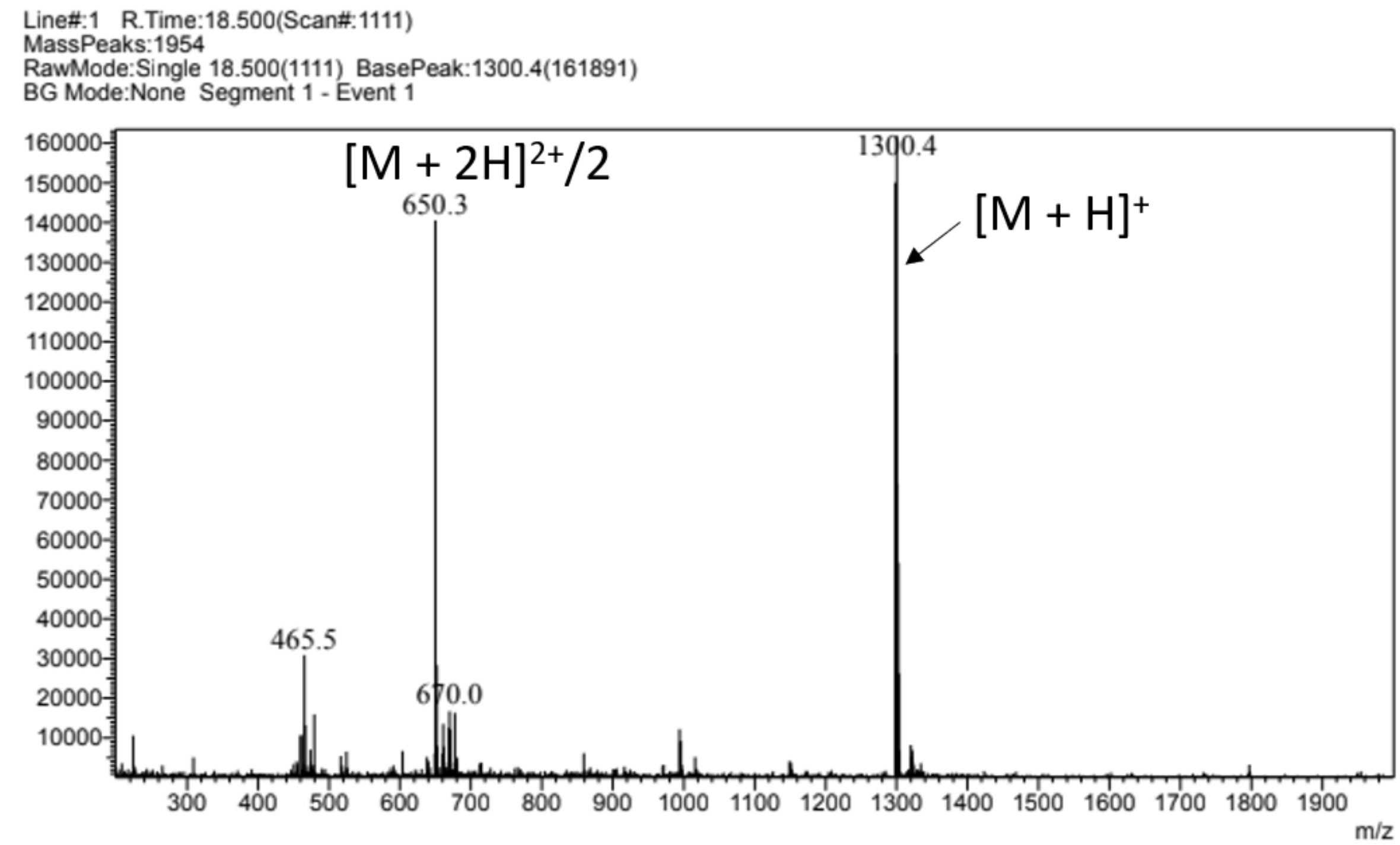

SI Figure 16. Mass spectrum of the peptidyl-thioester **1d - MP.**

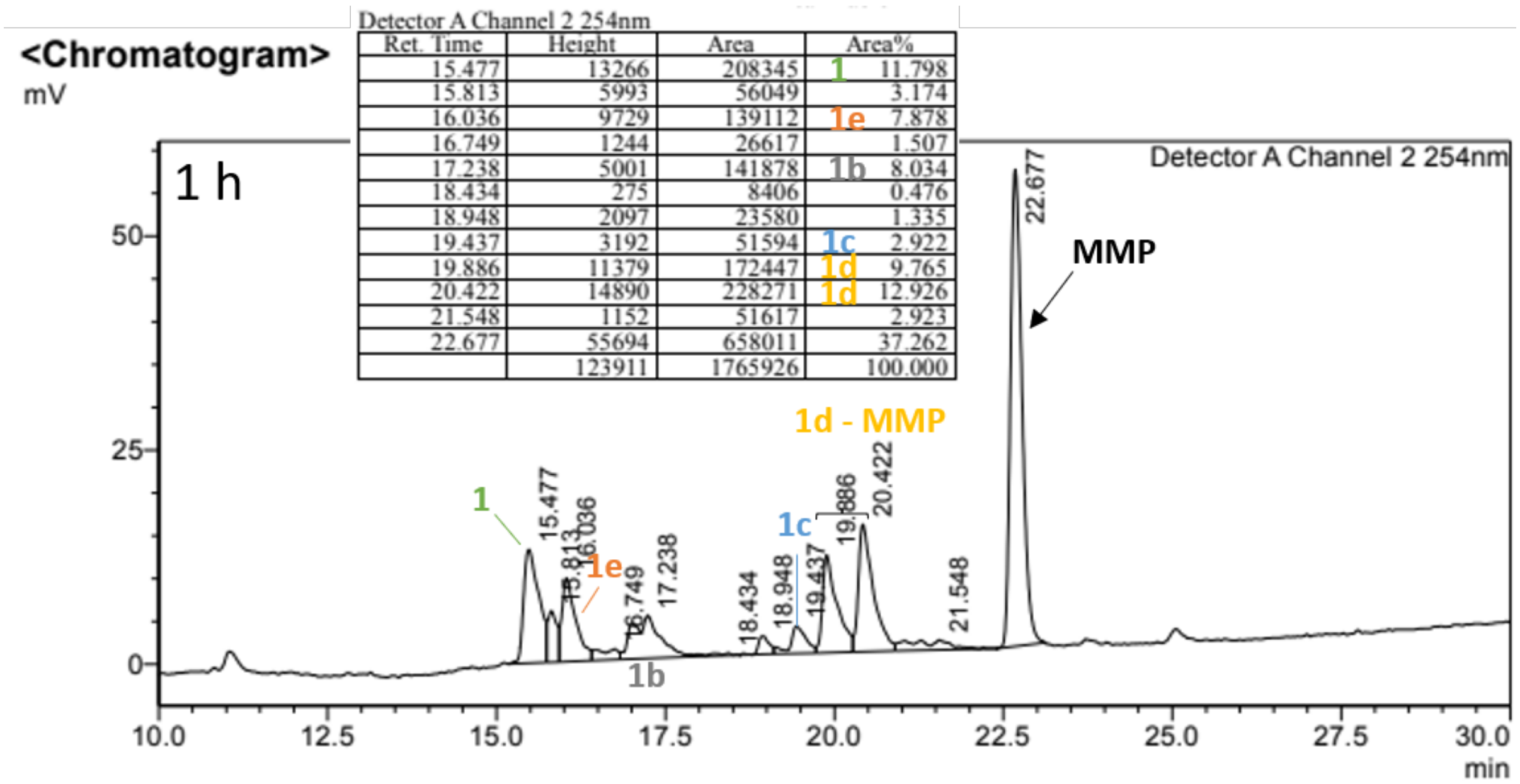

Detector A Channel 2 254nm

Ret. Time	Height	Area	Area%
15.477	13266	208345	1 11.798
15.813	5993	56049	3.174
16.036	9729	139112	1e 7.878
16.749	1244	26617	1.507
17.238	5001	141878	1b 8.034
18.434	275	8406	0.476
18.948	2097	23580	1.335
19.437	3192	51594	1c 2.922
19.886	11379	172447	1d 9.765
20.422	14890	228271	1d 12.926
21.548	1152	51617	2.923
22.677	55694	658011	37.262
	123911	1765926	100.000

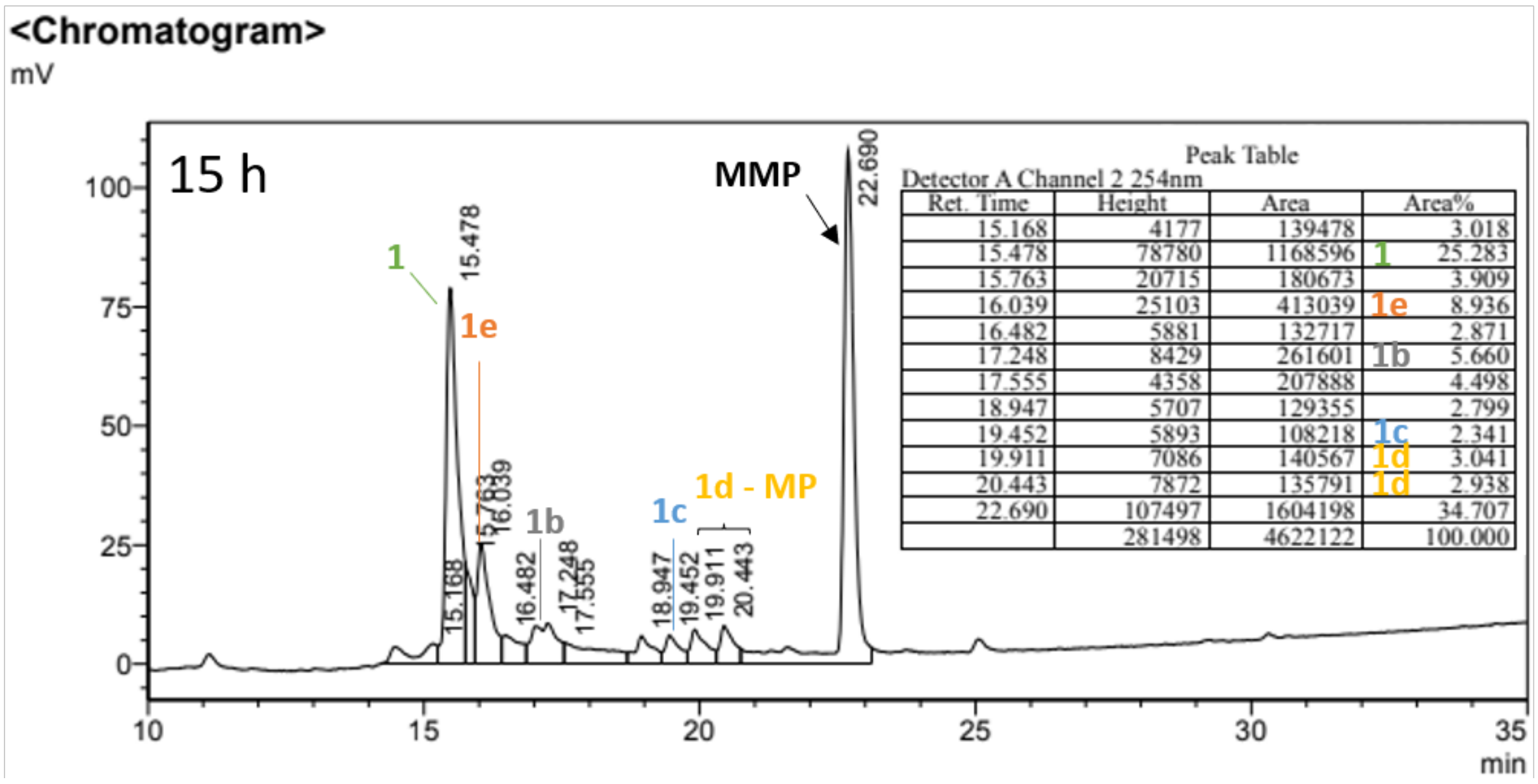

Peak Table

Detector A Channel 2 254nm

Ret. Time	Height	Area	Area%
15.168	4177	139478	3.018
15.478	78780	1168596	1 25.283
15.763	20715	180673	3.909
16.039	25103	413039	1e 8.936
16.482	5881	132717	2.871
17.248	8429	261601	1b 5.660
17.555	4358	207888	4.498
18.947	5707	129355	2.799
19.452	5893	108218	1c 2.341
19.911	7086	140567	1d 3.041
20.443	7872	135791	1d 2.938
22.690	107497	1604198	34.707
	281498	4622122	100.000

SI Figure 17. LC chromatogram monitoring the reaction at 1 h (Top) and 15 h (Bottom) and table data before normalisation in the link to **Figure 3** (entry MMP – 4-methoxymercaptophenol).

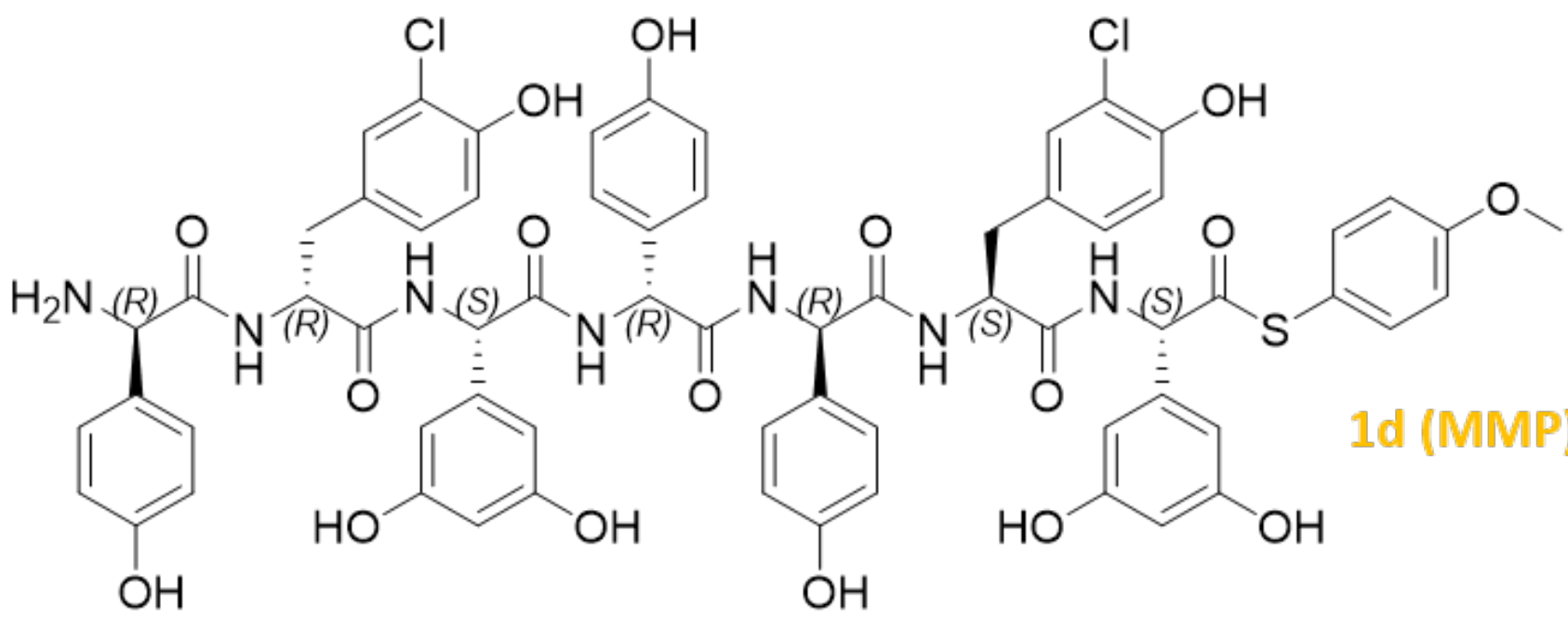

1d (MMP)

Chemical Formula: $C_{65}H_{59}Cl_2N_7O_{17}S$
Exact Mass: 1311.31
Molecular Weight: 1313.18

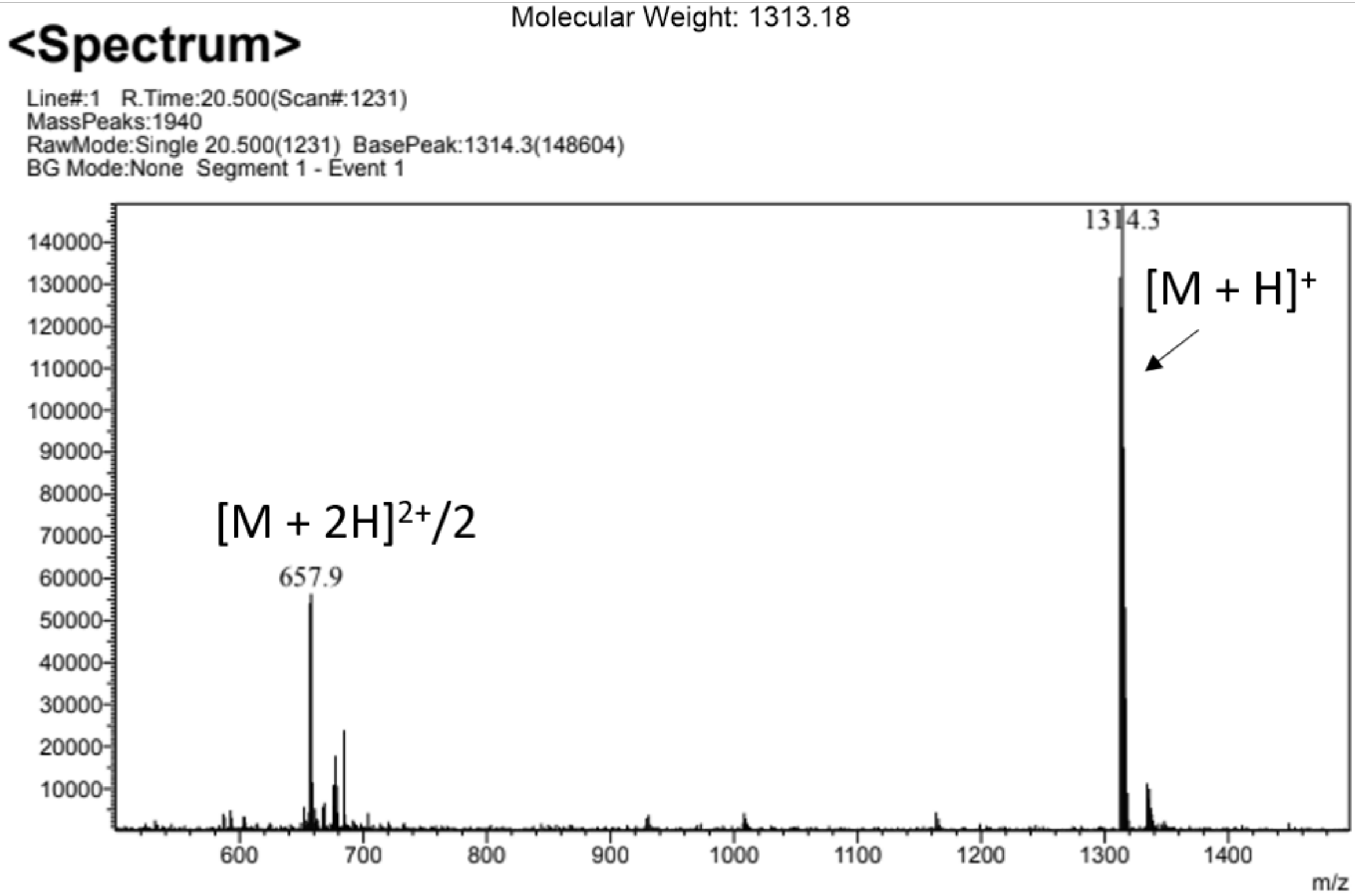

SI Figure 18. Mass spectrum of the peptidyl-thioester **1d - MMP.**

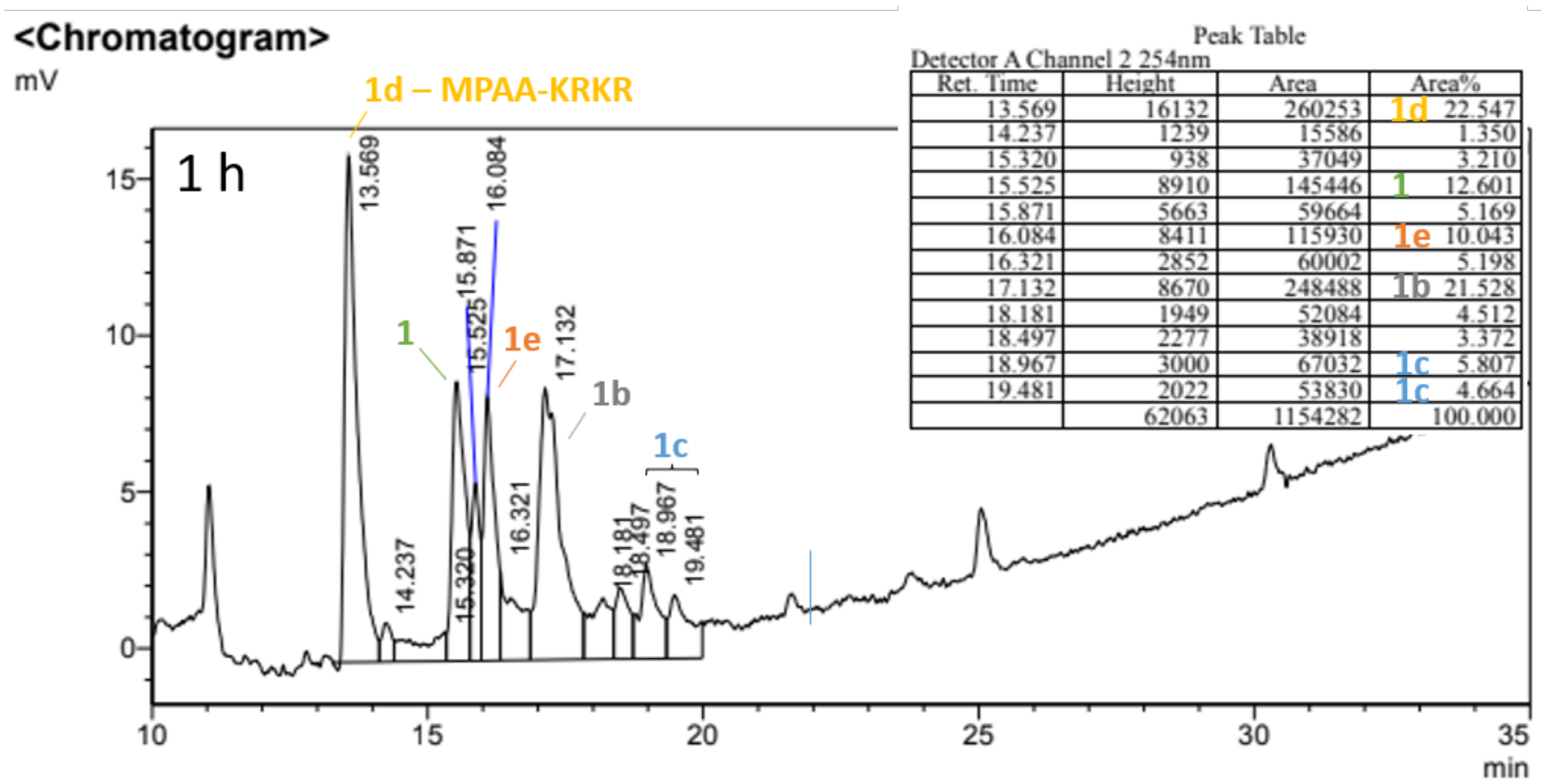

Peak Table
Detector A Channel 2 254nm

Ret. Time	Height	Area		Area%
13.569	16132	260253	1d	22.547
14.237	1239	15586		1.350
15.320	938	37049		3.210
15.525	8910	145446	1	12.601
15.871	5663	59664		5.169
16.084	8411	115930	1e	10.043
16.321	2852	60002		5.198
17.132	8670	248488	1b	21.528
18.181	1949	52084		4.512
18.497	2277	38918		3.372
18.967	3000	67032	1c	5.807
19.481	2022	53830	1c	4.664
	62063	1154282		100.000

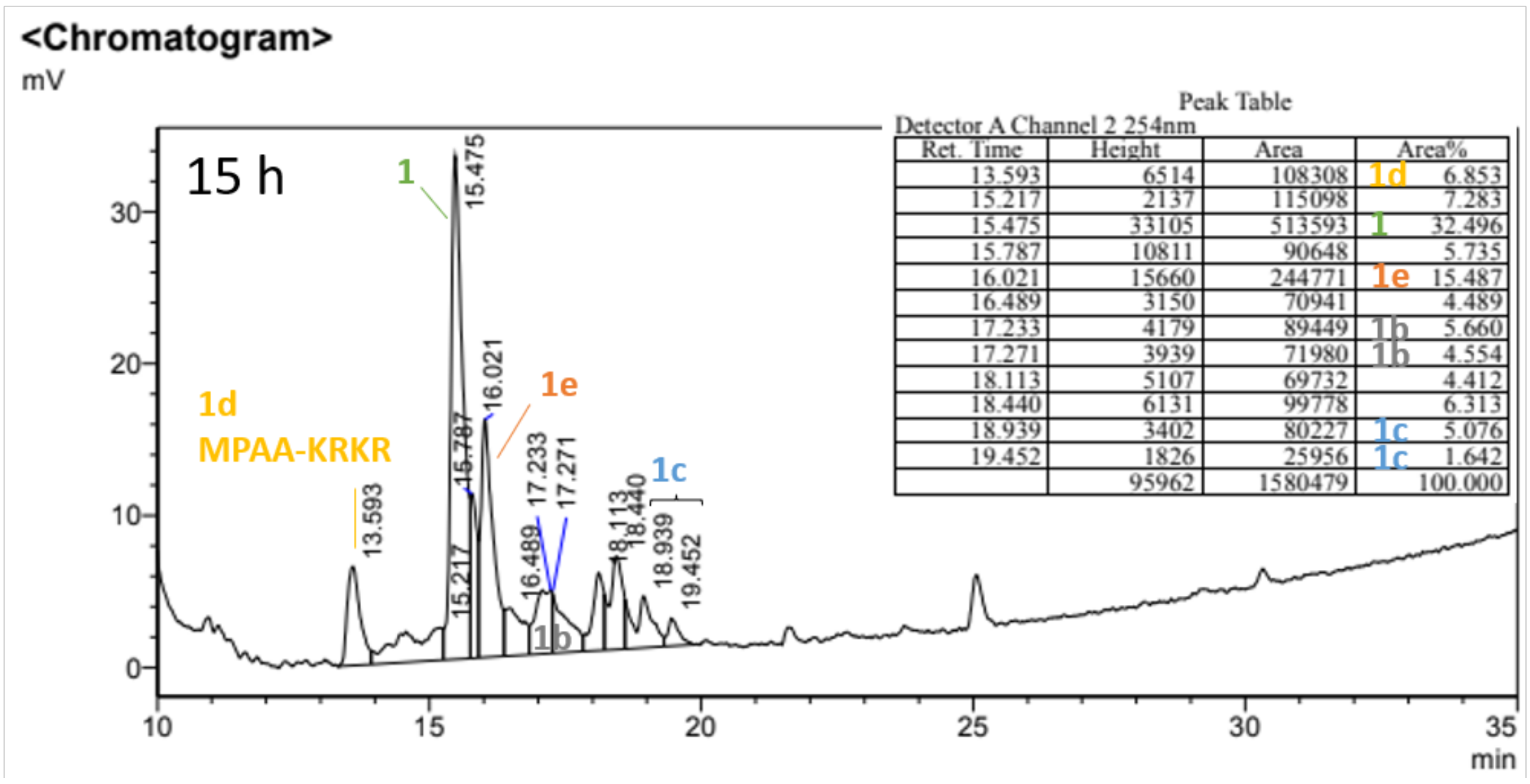

Peak Table
Detector A Channel 2 254nm

Ret. Time	Height	Area		Area%
13.593	6514	108308	1d	6.853
15.217	2137	115098		7.283
15.475	33105	513593	1	32.496
15.787	10811	90648		5.735
16.021	15660	244771	1e	15.487
16.489	3150	70941		4.489
17.233	4179	89449	1b	5.660
17.271	3939	71980	1b	4.554
18.113	5107	69732		4.412
18.440	6131	99778		6.313
18.939	3402	80227	1c	5.076
19.452	1826	25956	1c	1.642
	95962	1580479		100.000

SI Figure 19. LC chromatogram monitoring the reaction at 1 h (Top) and 15 h (Bottom) and table data before normalisation in the link to **Figure 3** (entry **4** – MPAA-KRKR).

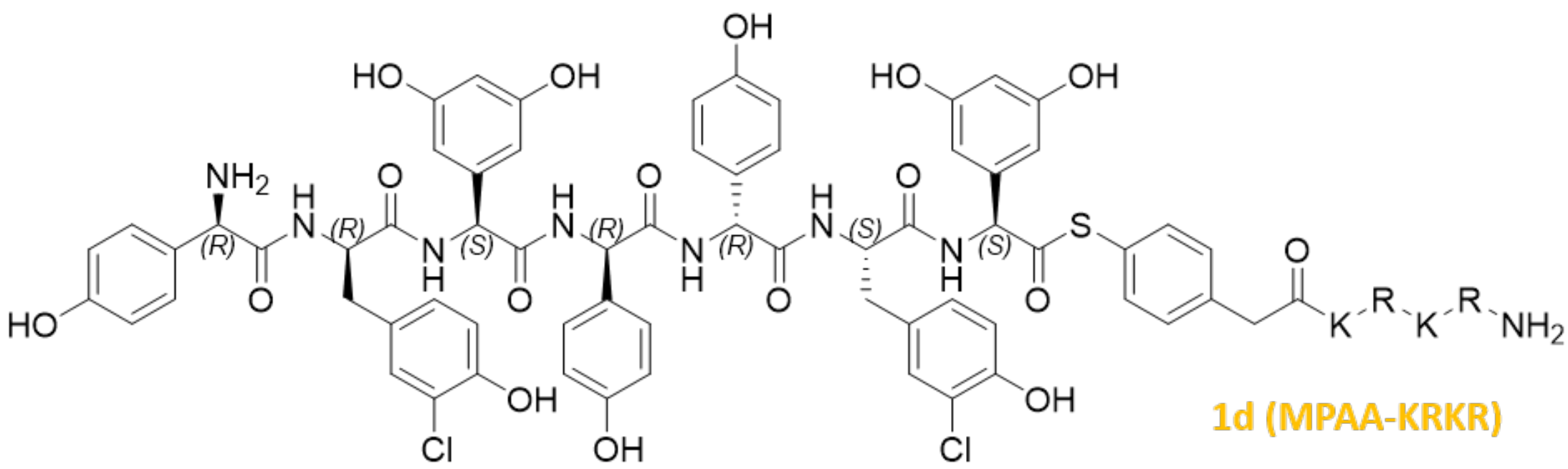

1d (MPAA-KRKR)

Chemical Formula: $C_{90}H_{108}Cl_2N_{20}O_{21}S$
Exact Mass: 1906.71
Molecular Weight: 1908.93

<Spectrum>

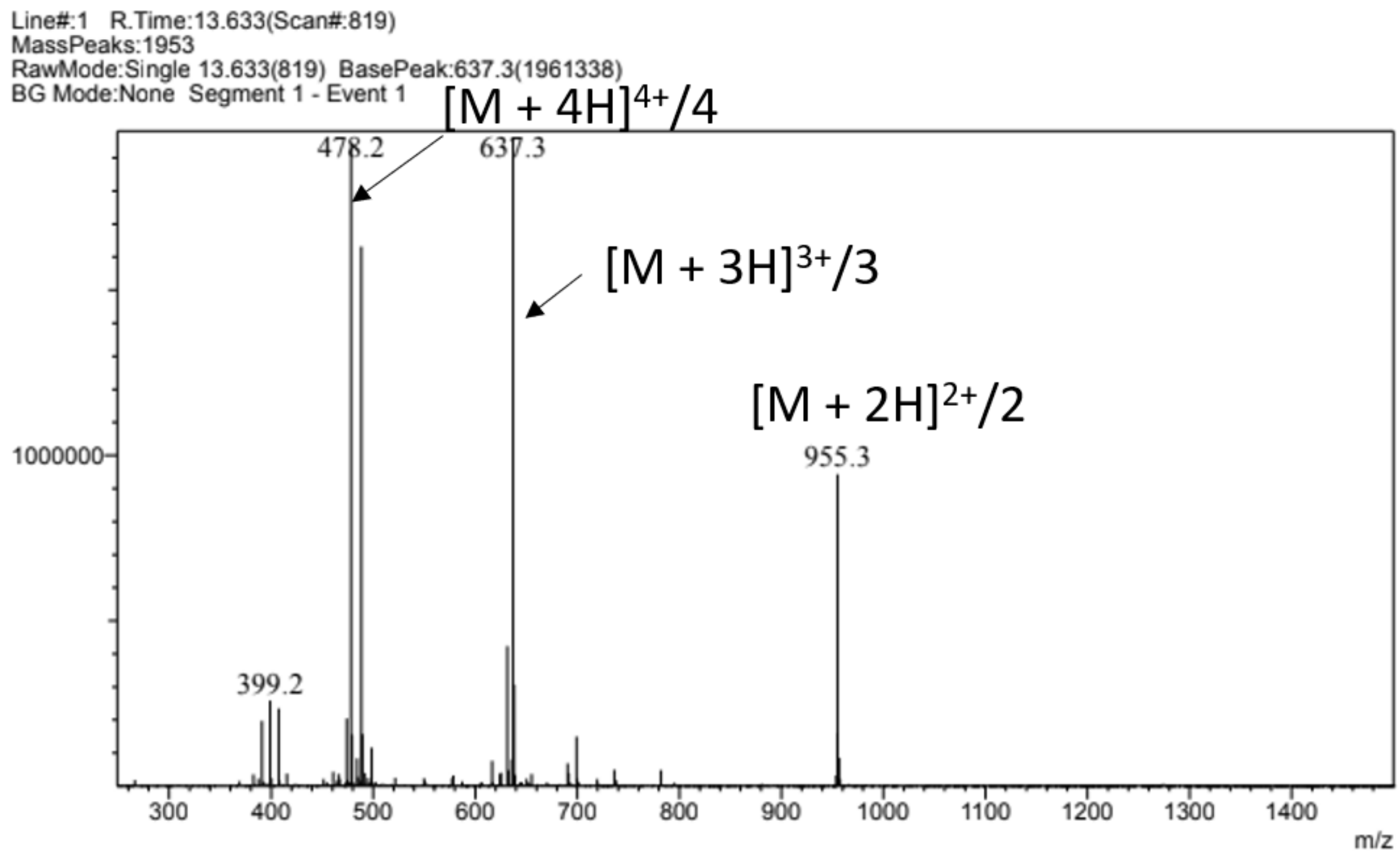

SI Figure 20. Mass spectrum of the peptidyl-thioester **1d – MPAA-KRKR.**

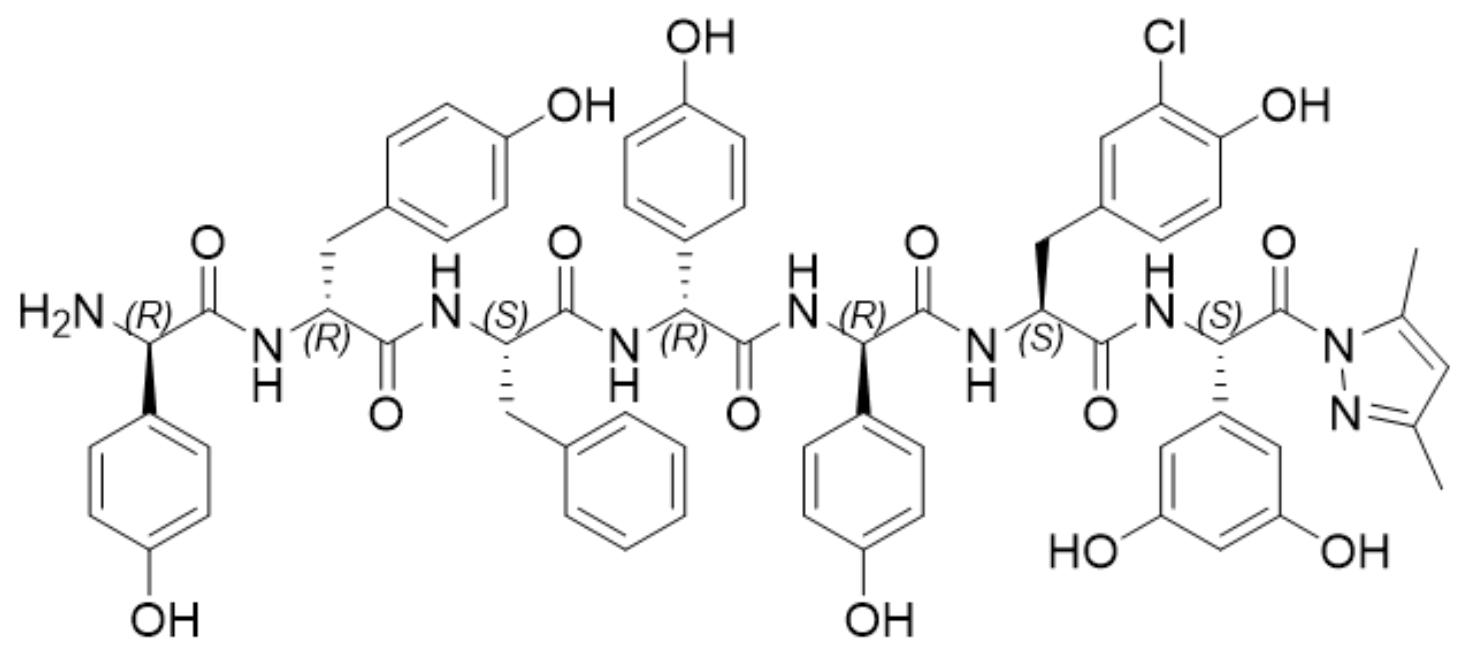

2c

Chemical Formula: $C_{64}H_{62}ClN_9O_{14}$
Exact Mass: 1215.41
Molecular Weight: 1216.70

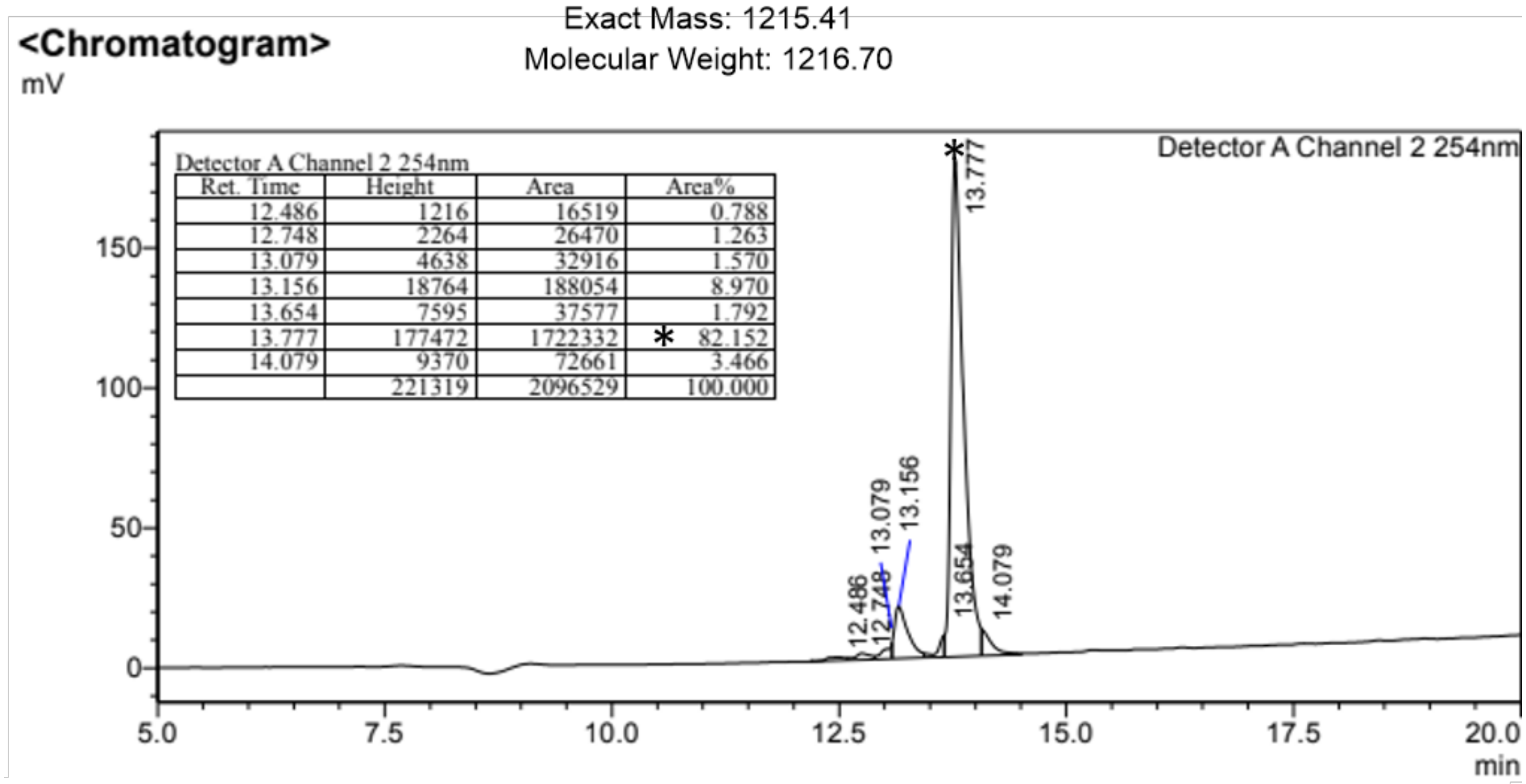

Detector A Channel 2 254nm

Ret. Time	Height	Area	Area%
12.486	1216	16519	0.788
12.748	2264	26470	1.263
13.079	4638	32916	1.570
13.156	18764	188054	8.970
13.654	7595	37577	1.792
13.777	177472	1722332	* 82.152
14.079	9370	72661	3.466
	221319	2096529	100.000

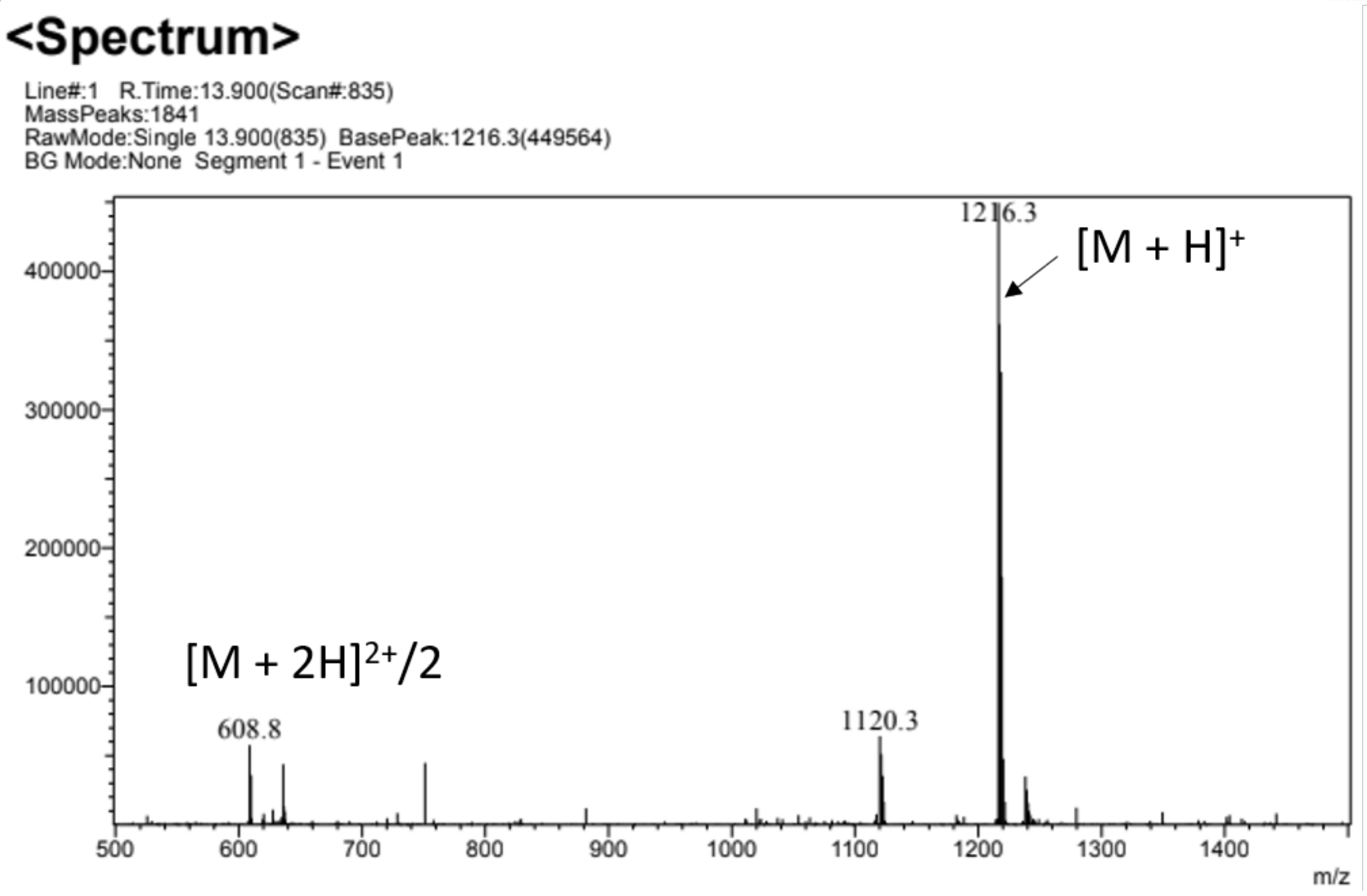

SI Figure 21. LCMS chromatogram and mass spectrum of the formation of **2c** in DMF at pH 3.

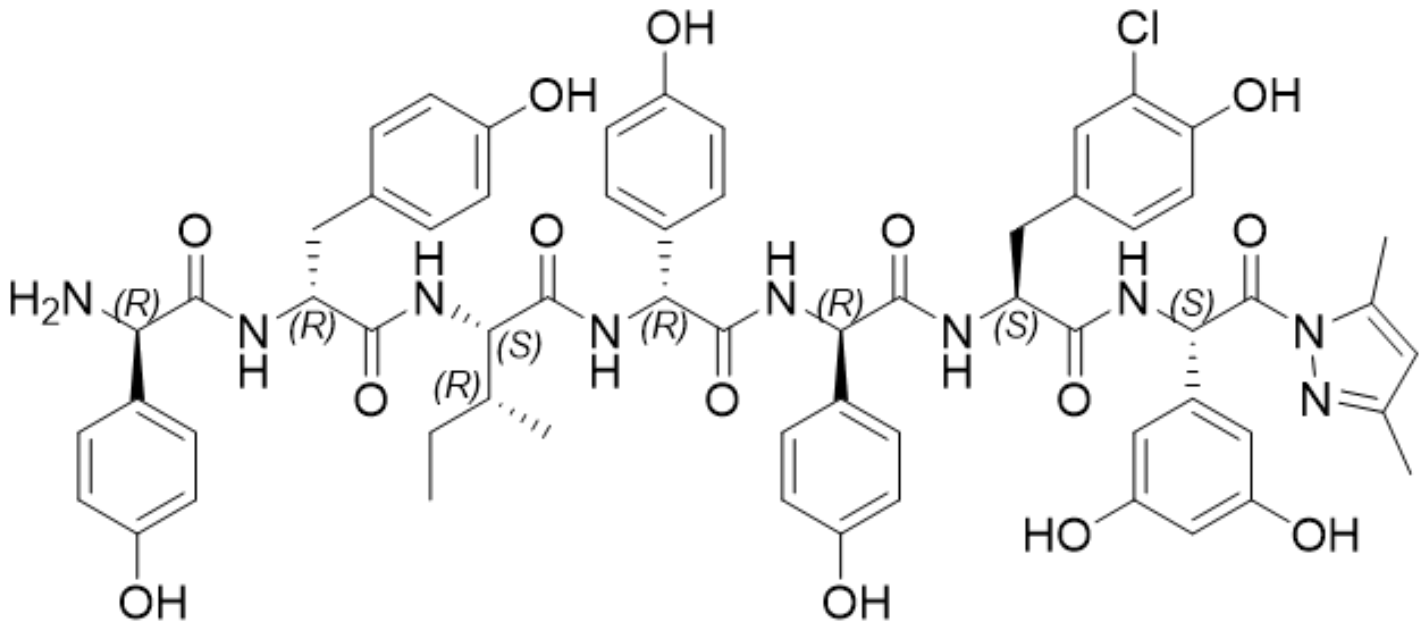

3c

Chemical Formula: $C_{61}H_{64}ClN_9O_{14}$

Exact Mass: 1181.43

Molecular Weight: 1182.68

<Chromatogram>

mV

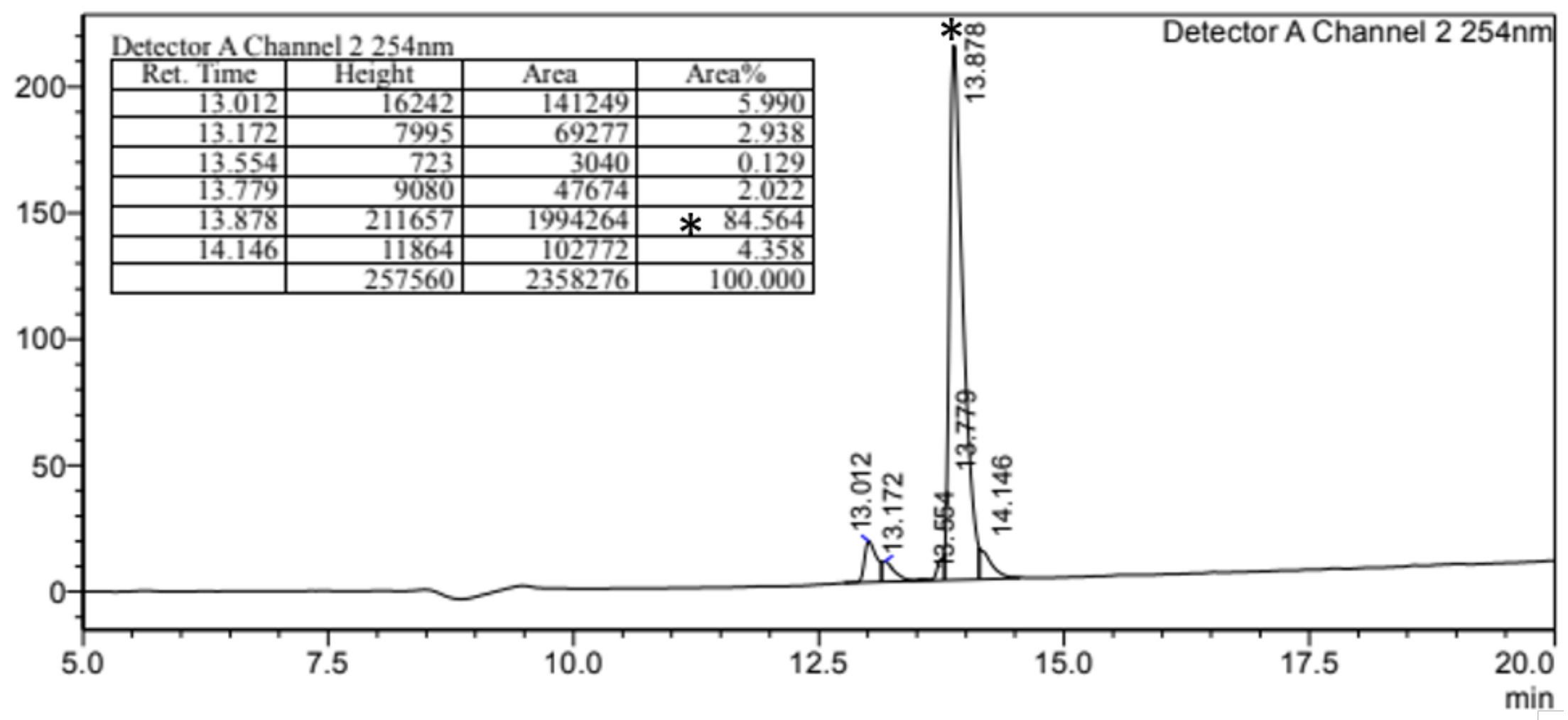

Detector A Channel 2 254nm

Ret. Time	Height	Area	Area%
13.012	16242	141249	5.990
13.172	7995	69277	2.938
13.554	723	3040	0.129
13.779	9080	47674	2.022
13.878	211657	1994264	* 84.564
14.146	11864	102772	4.358
	257560	2358276	100.000

<Spectrum>

Line#:1 R.Time:14.000(Scan#:841)
MassPeaks:1838
RawMode:Single 14.000(841) BasePeak:1182.4(587161)
BG Mode:None Segment 1 - Event 1

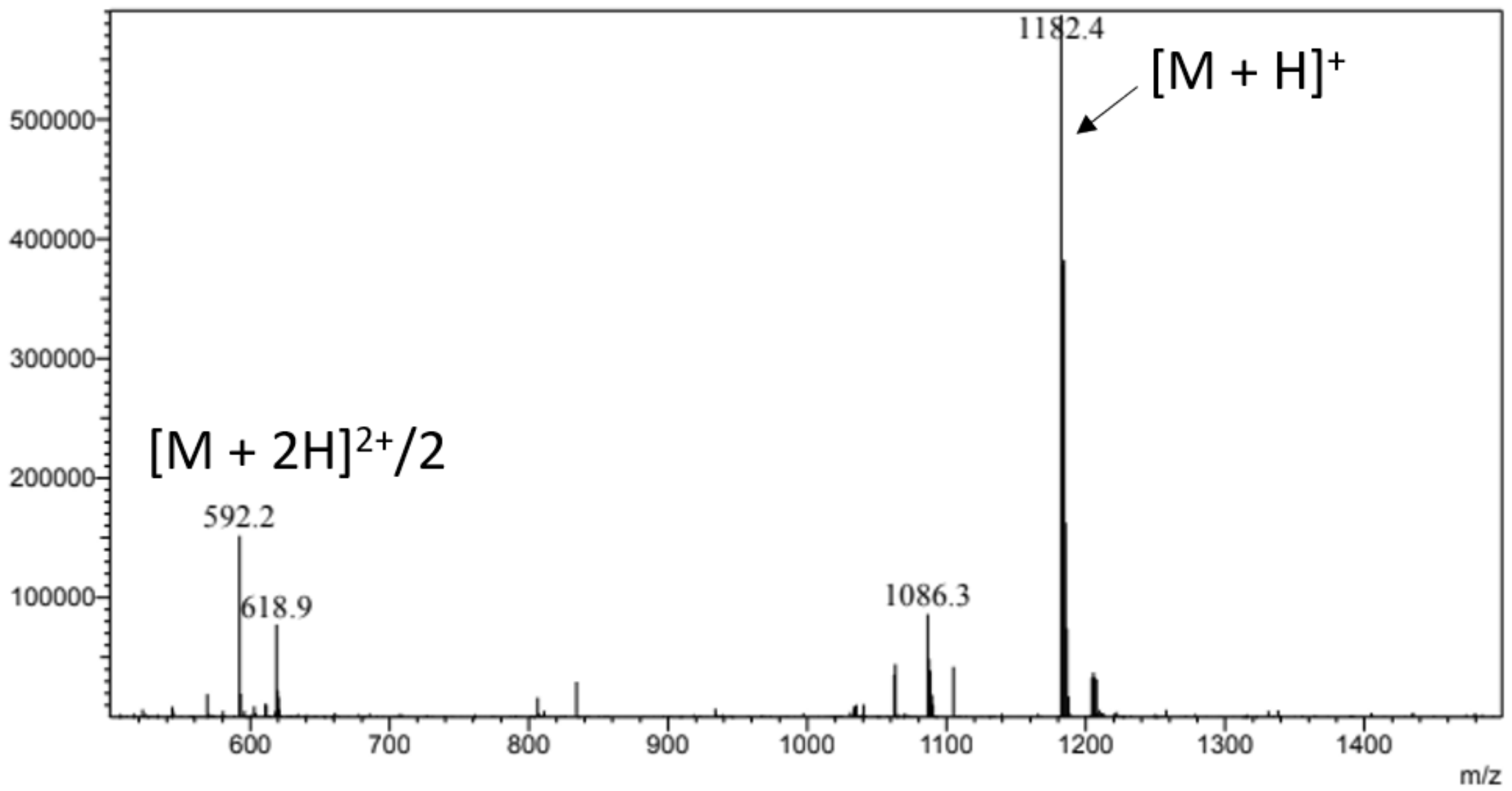

SI Figure 22. LCMS chromatogram and mass spectrum of the formation of **3c** in DMF at pH 3.

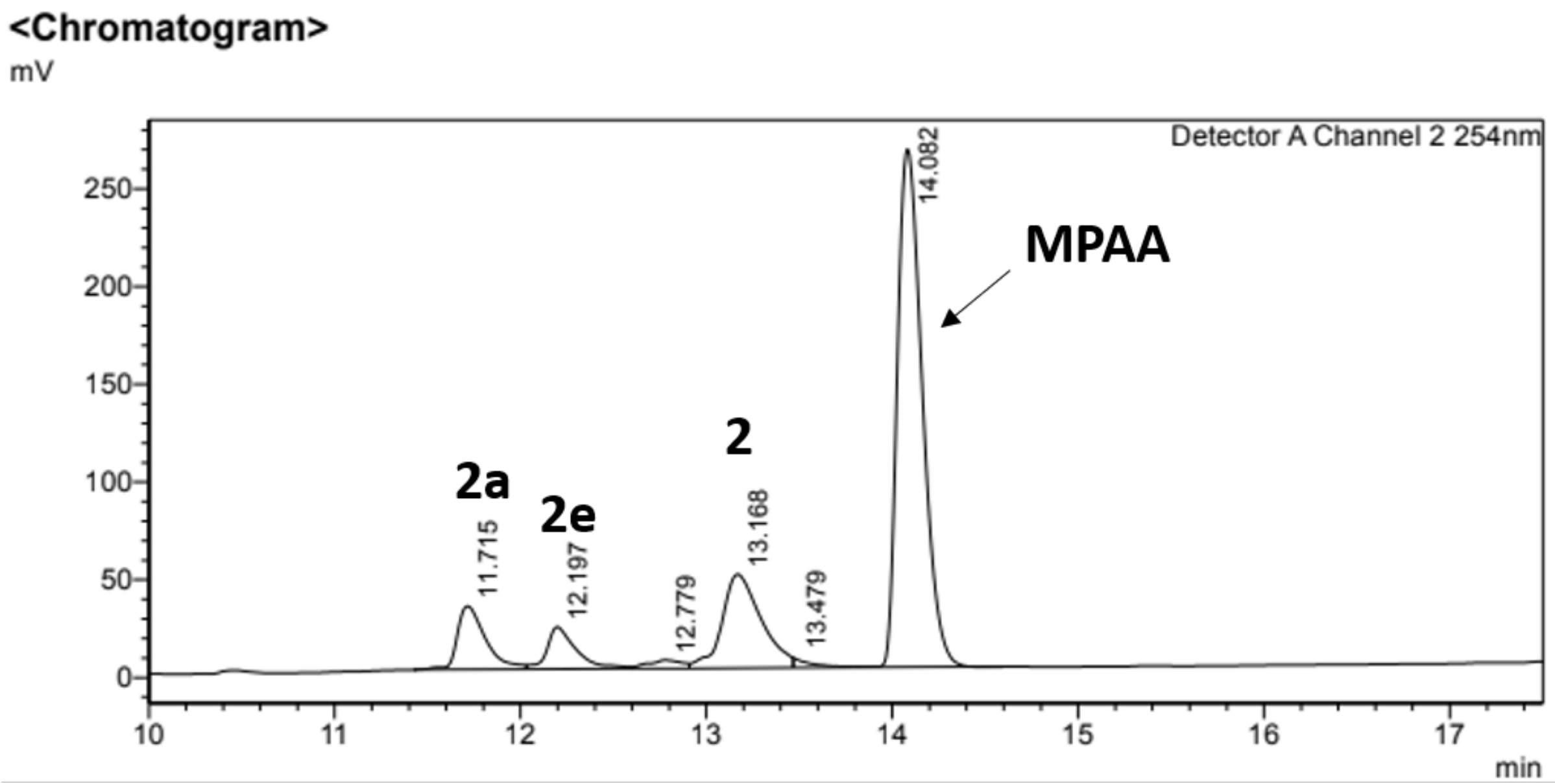

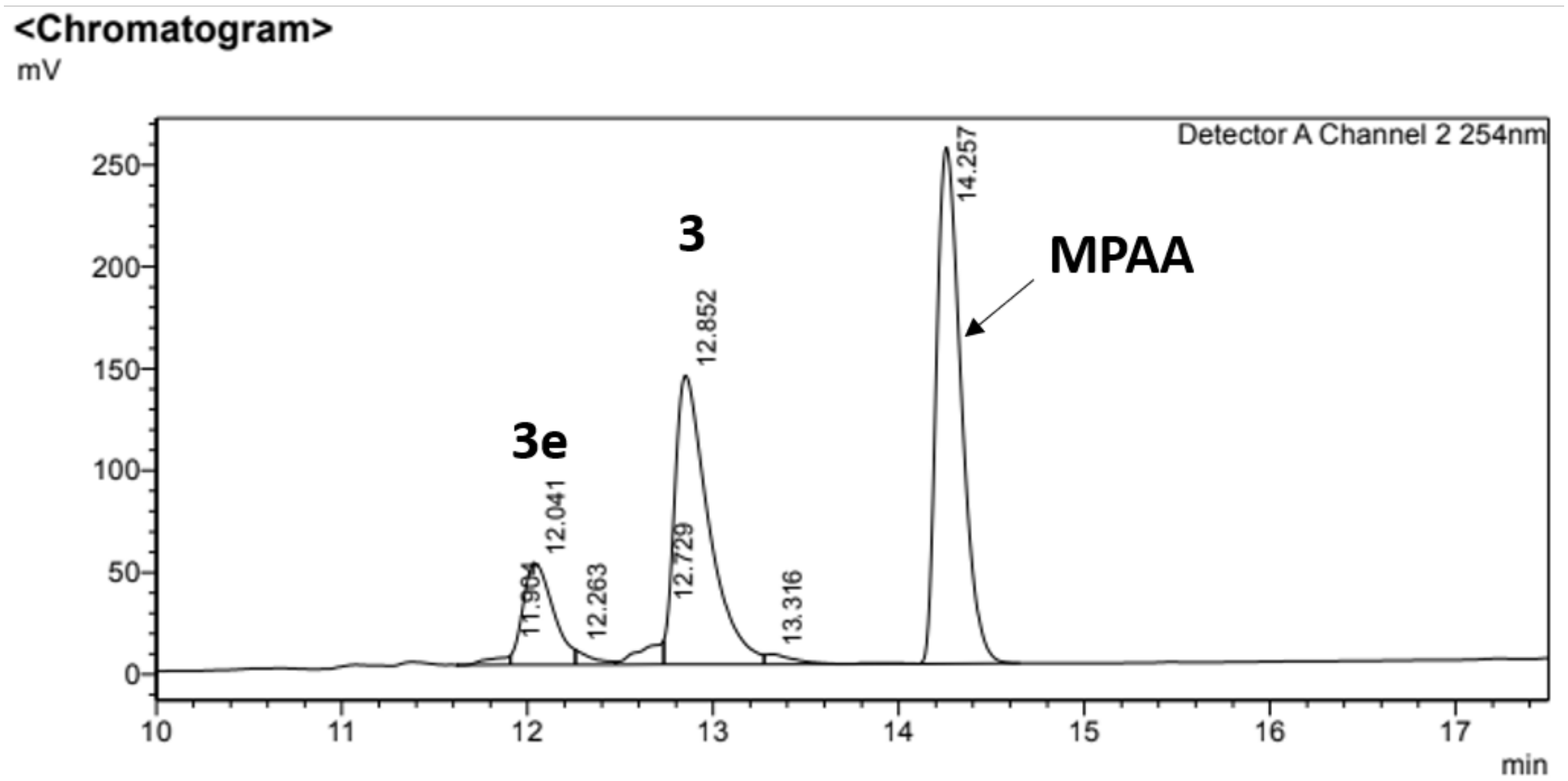

SI Figure 23. LC chromatograms monitoring the peptidyl-coenzyme A formation of **2** (top) and **3** (bottom)

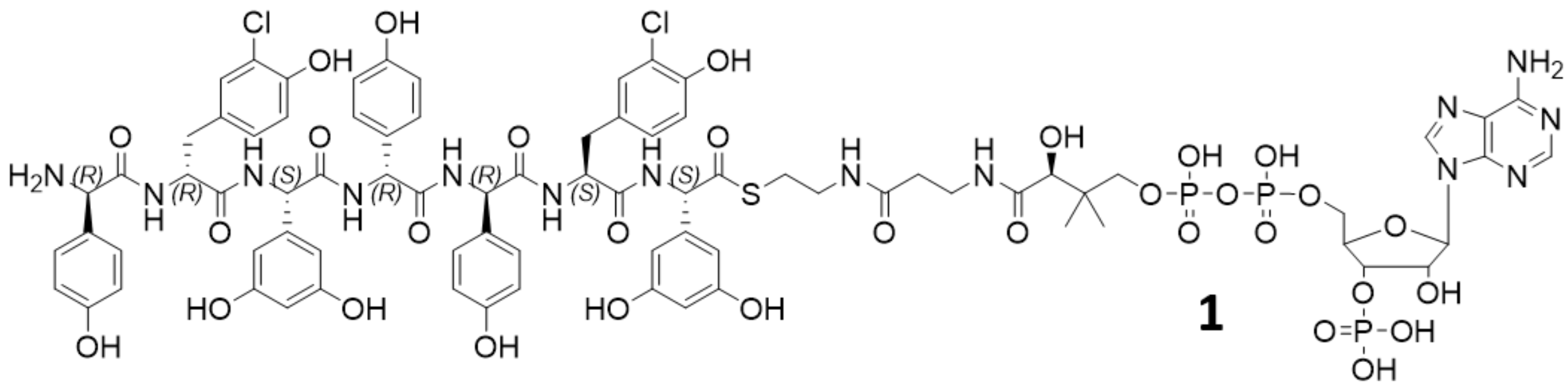

1

Chemical Formula: $C_{79}H_{87}Cl_2N_{14}O_{32}P_3S$
Exact Mass: 1938.39
Molecular Weight: 1940.51

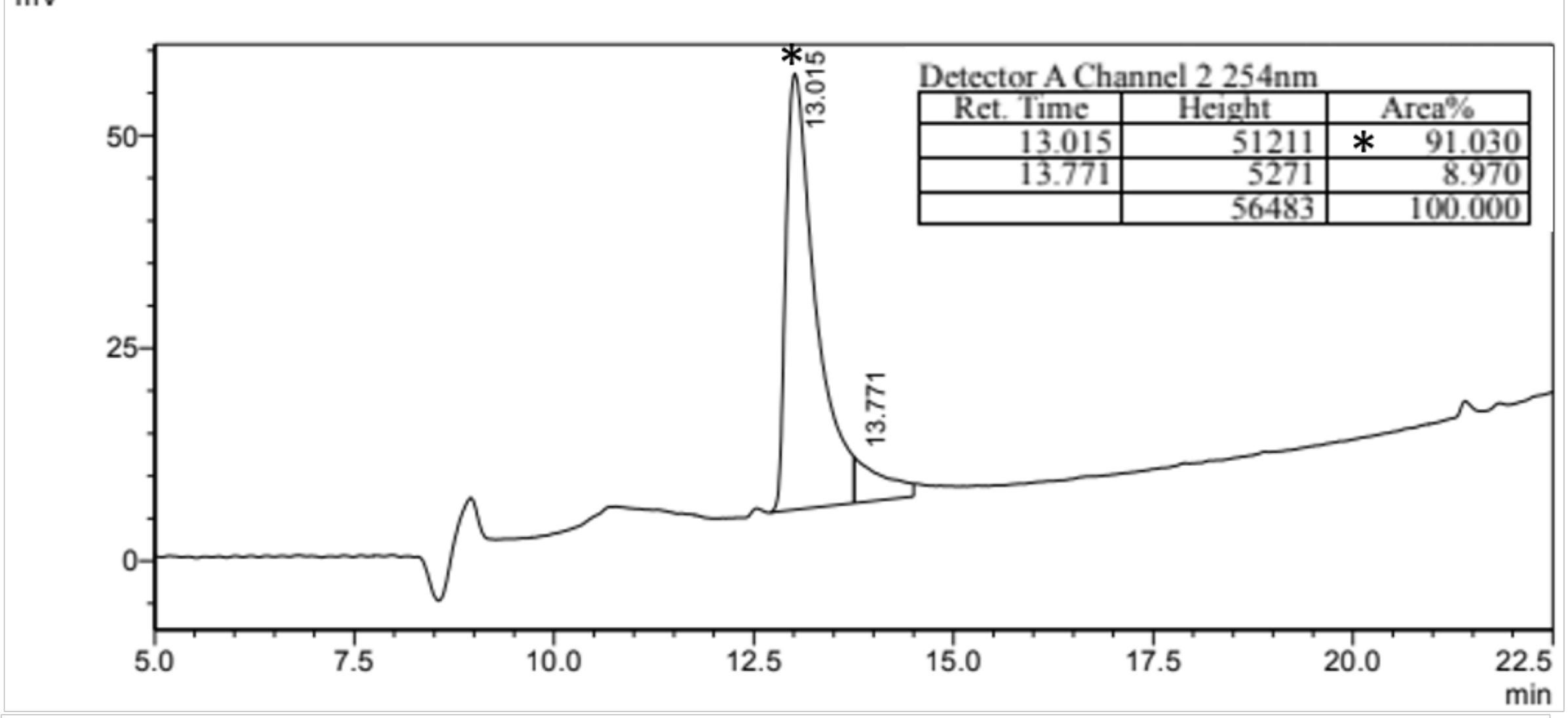

Ret. Time	Height	Area%
13.015	51211	* 91.030
13.771	5271	8.970
	56483	100.000

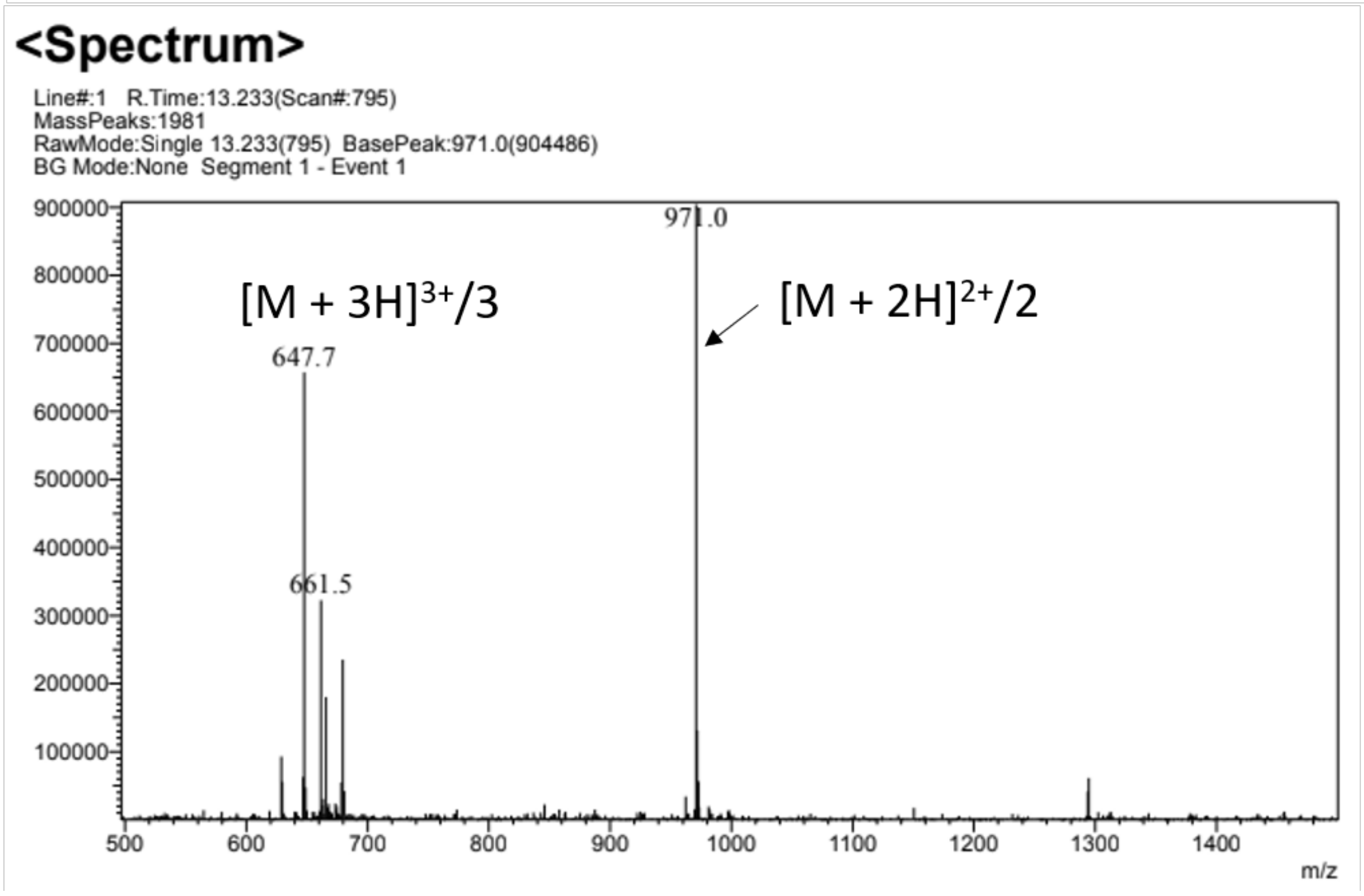

SI Figure 24. LCMS chromatogram and mass spectrum of **1**.

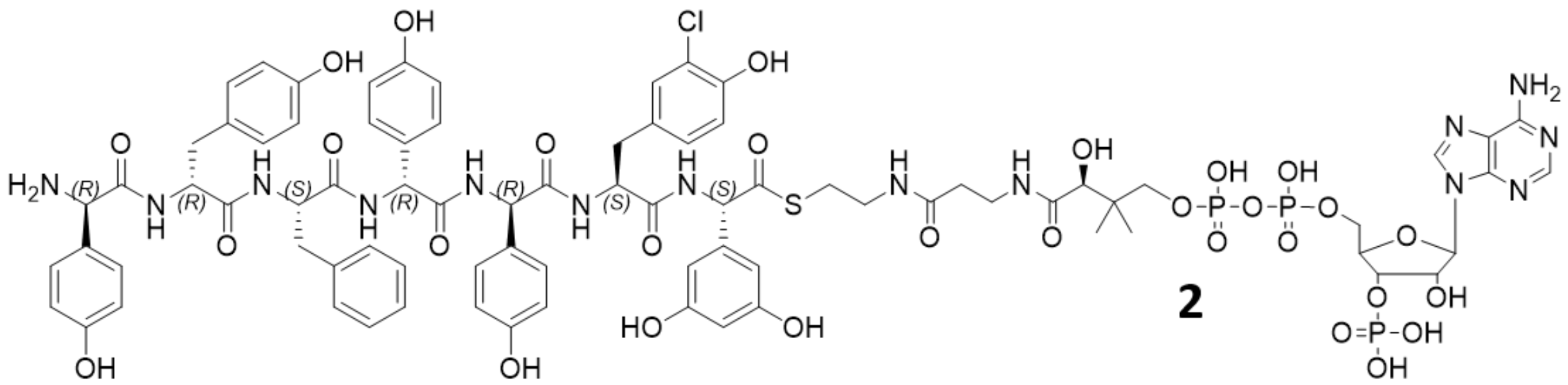

2

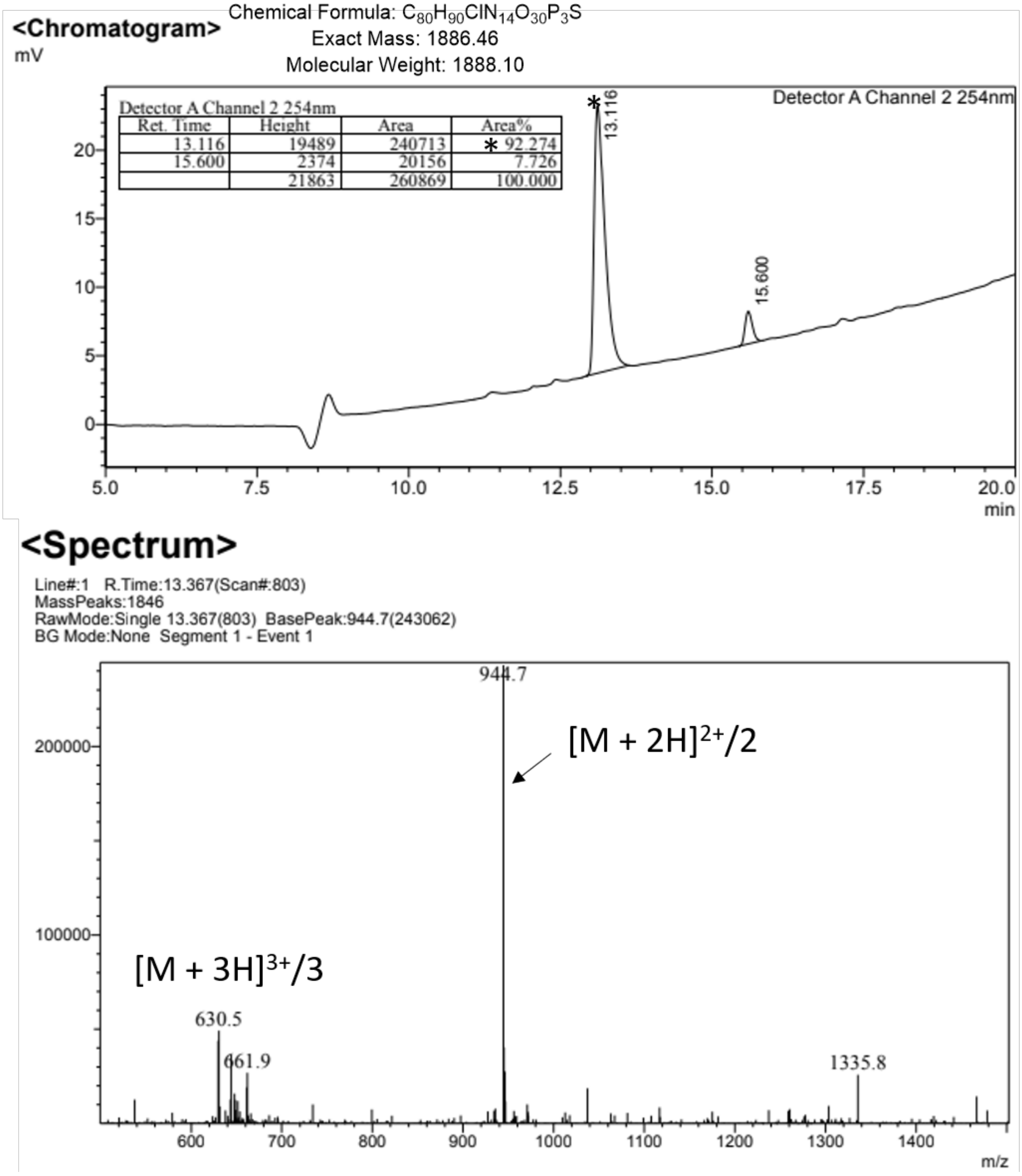

Detector A Channel 2 254nm

Ret. Time	Height	Area	Area%
13.116	19489	240713	* 92.274
15.600	2374	20156	7.726
	21863	260869	100.000

SI Figure 25. LCMS chromatogram and mass spectrum of **2**.

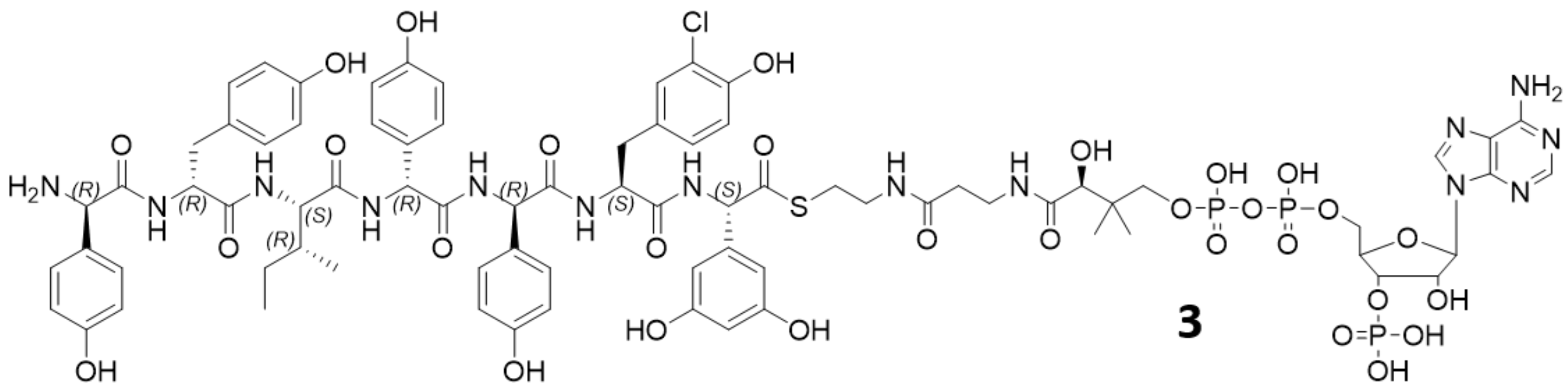

Chemical Formula: $C_{77}H_{92}ClN_{14}O_{30}P_3S$
Exact Mass: 1852.47
Molecular Weight: 1854.08

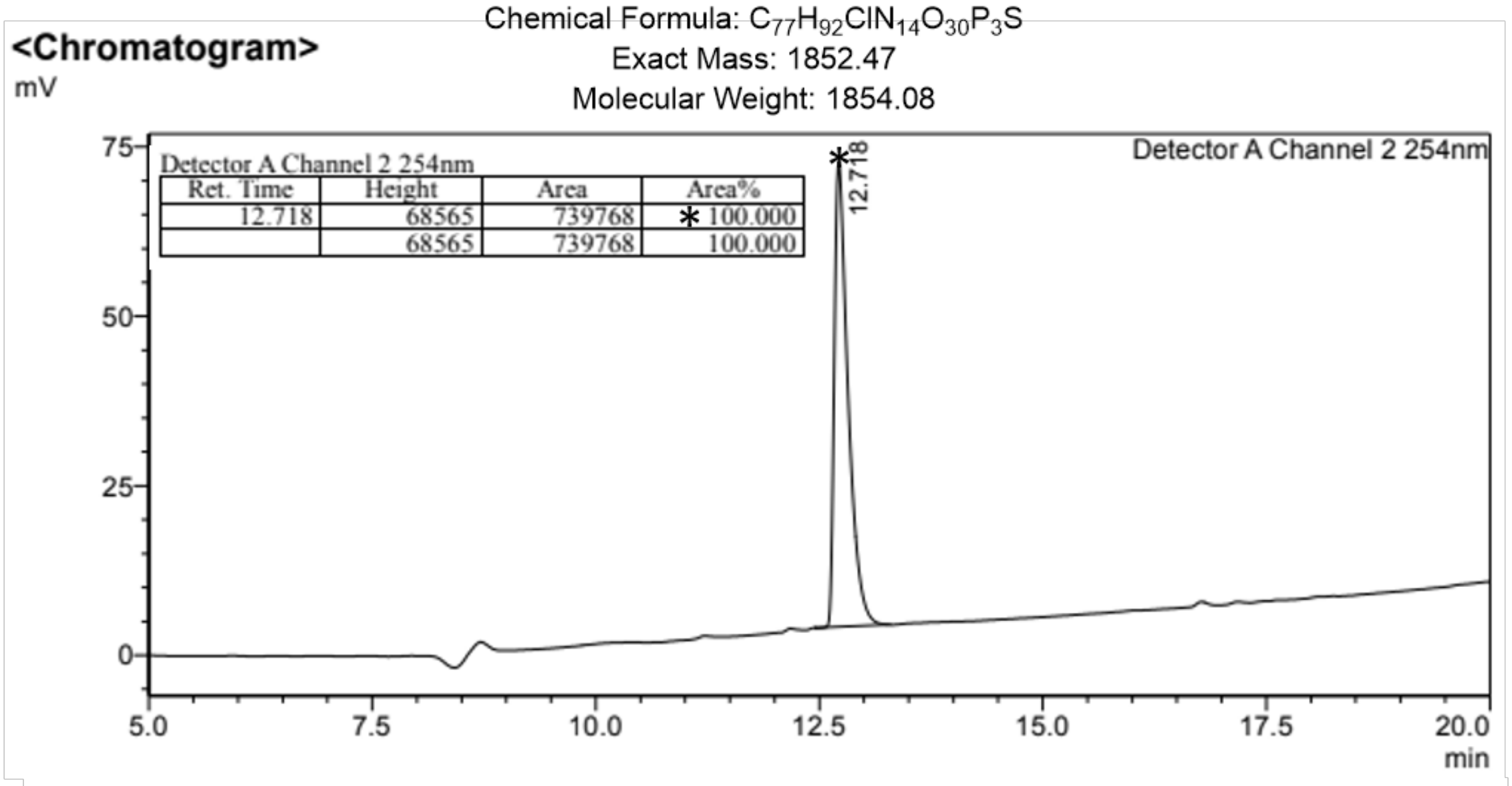

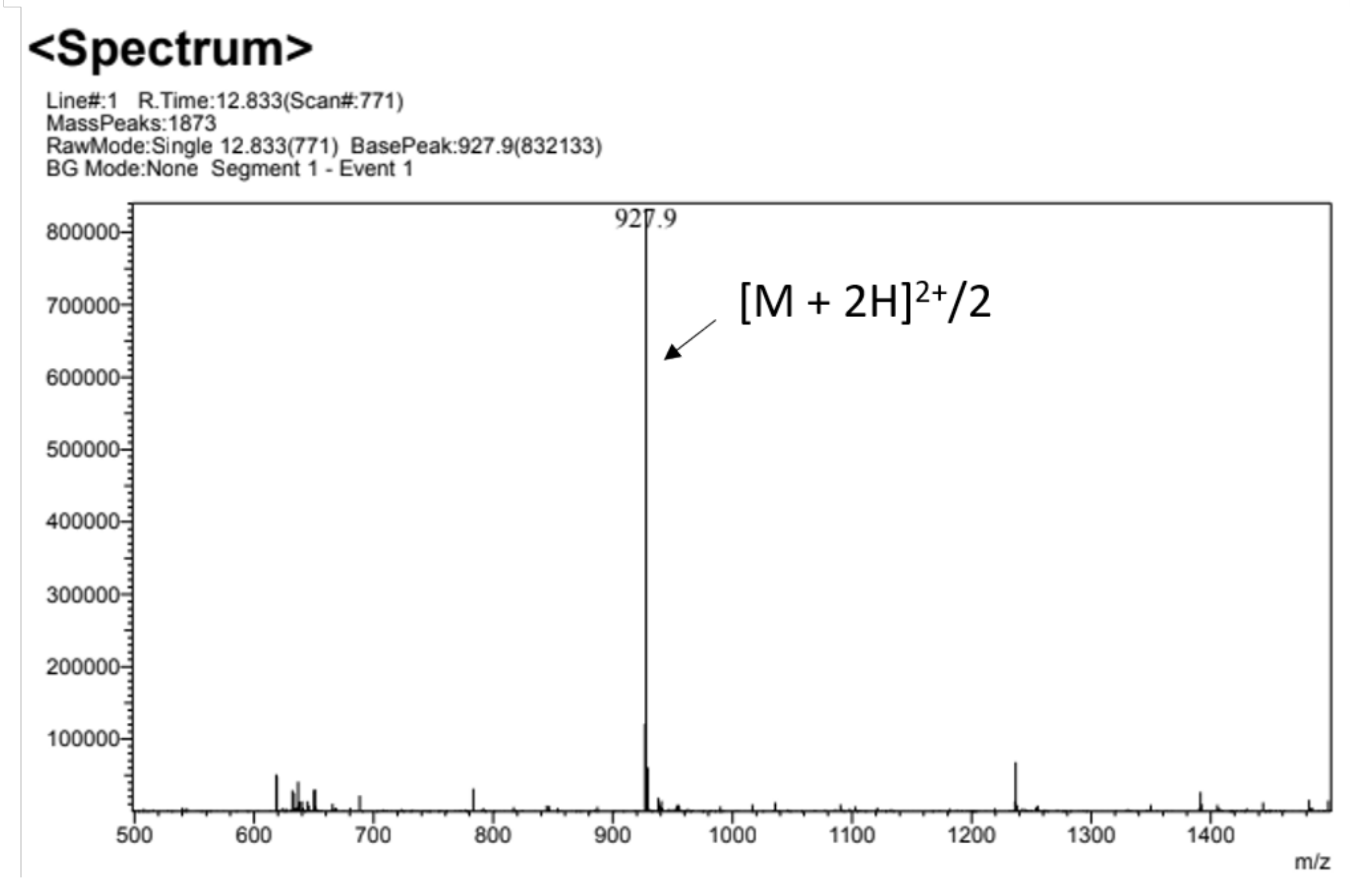

SI Figure 26. LCMS chromatogram and mass spectrum of **3**.

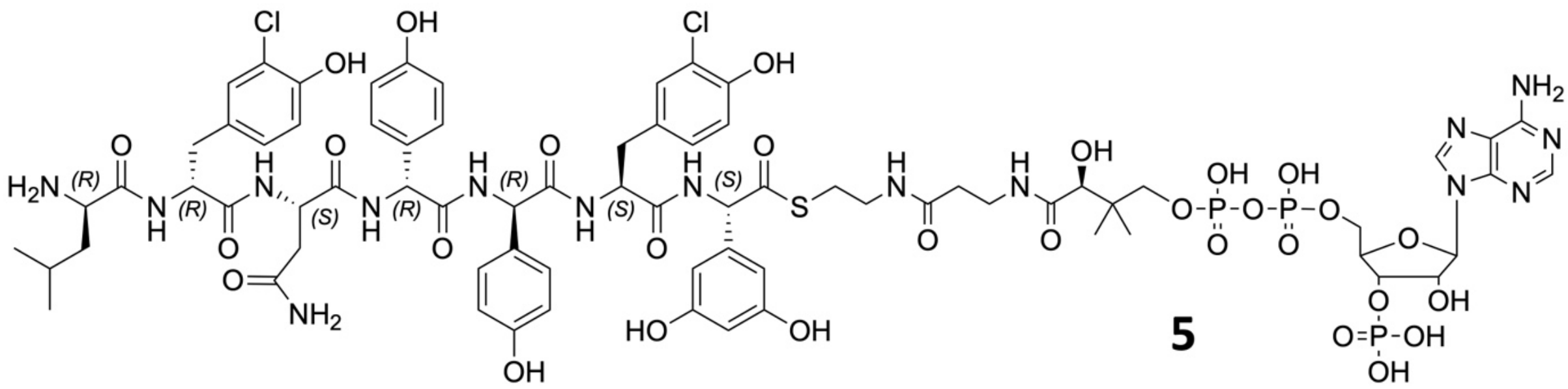

5

Chemical Formula: $C_{73}H_{90}Cl_2N_{15}O_{30}P_3S$
Exact Mass: 1851.42886
Molecular Weight: 1853.47929

<Chromatogram>

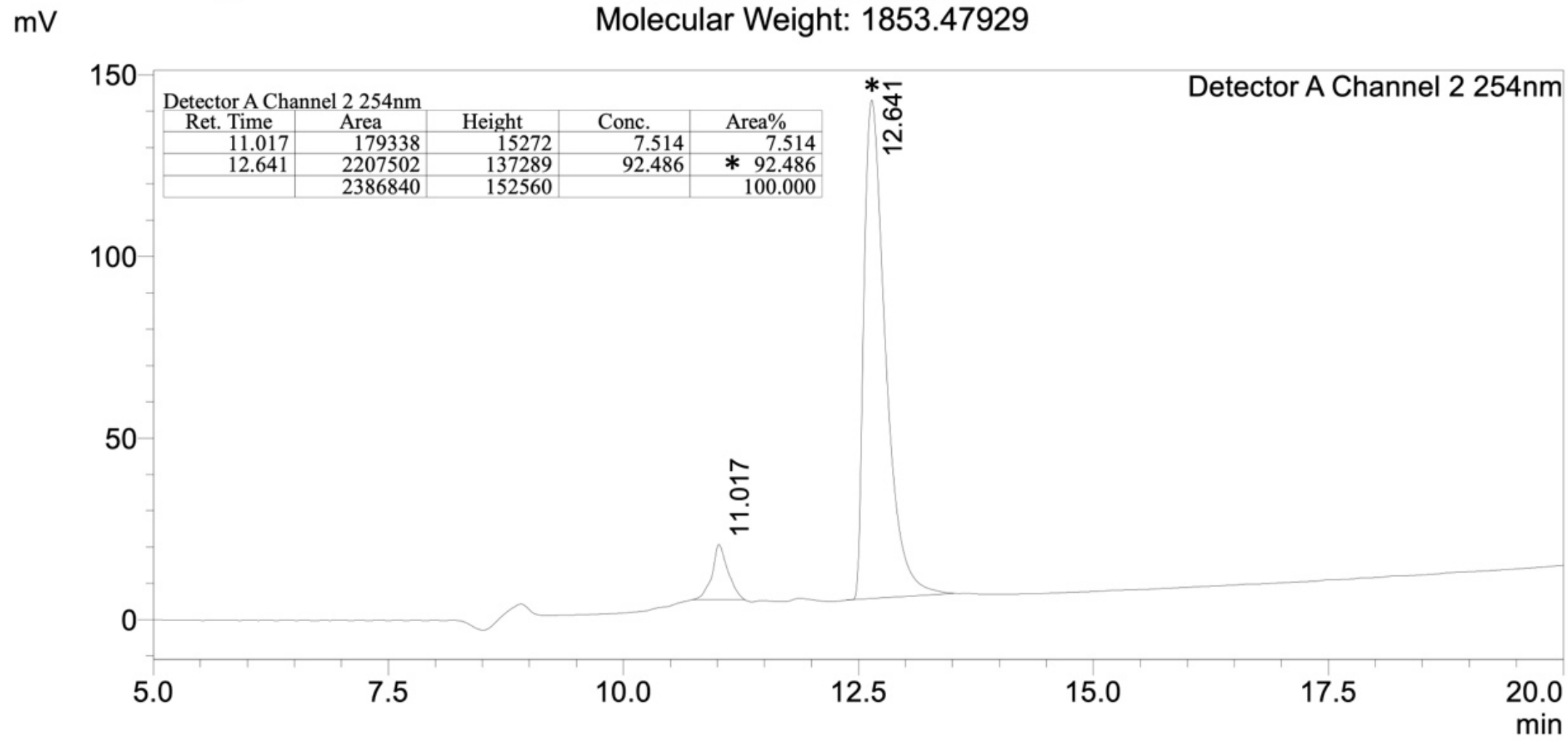

Detector A Channel 2 254nm

Ret. Time	Area	Height	Conc.	Area%
11.017	179338	15272	7.514	7.514
12.641	2207502	137289	92.486	* 92.486
	2386840	152560		100.000

<Spectrum>

Line#:1 R.Time:12.833(Scan#:771)
MassPeaks:2004
RawMode:Single 12.833(771) BasePeak:927.7(4098612)
BG Mode:None Segment 1 - Event 1

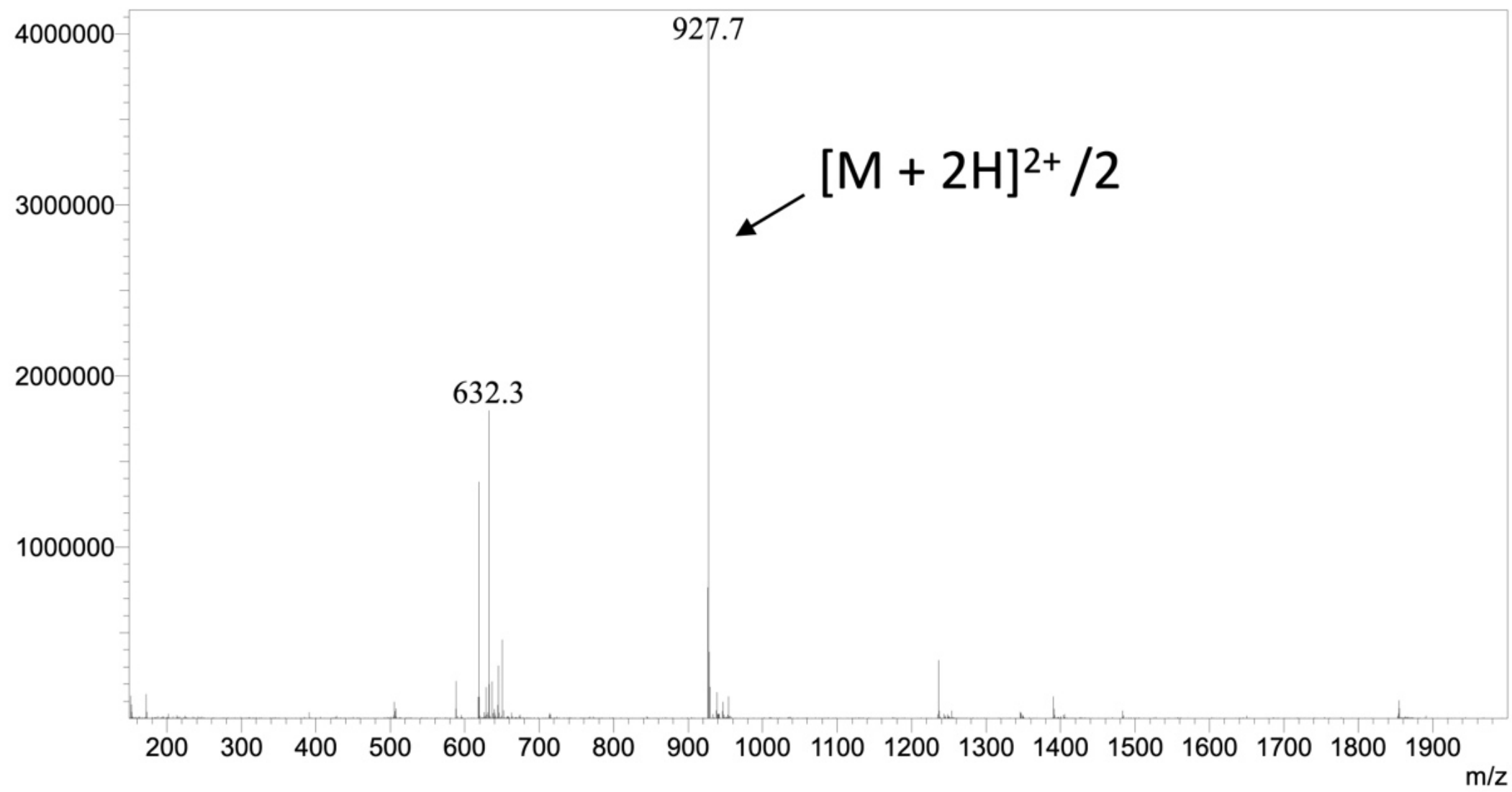

SI Figure 27. LCMS chromatogram and mass spectrum of **5**.

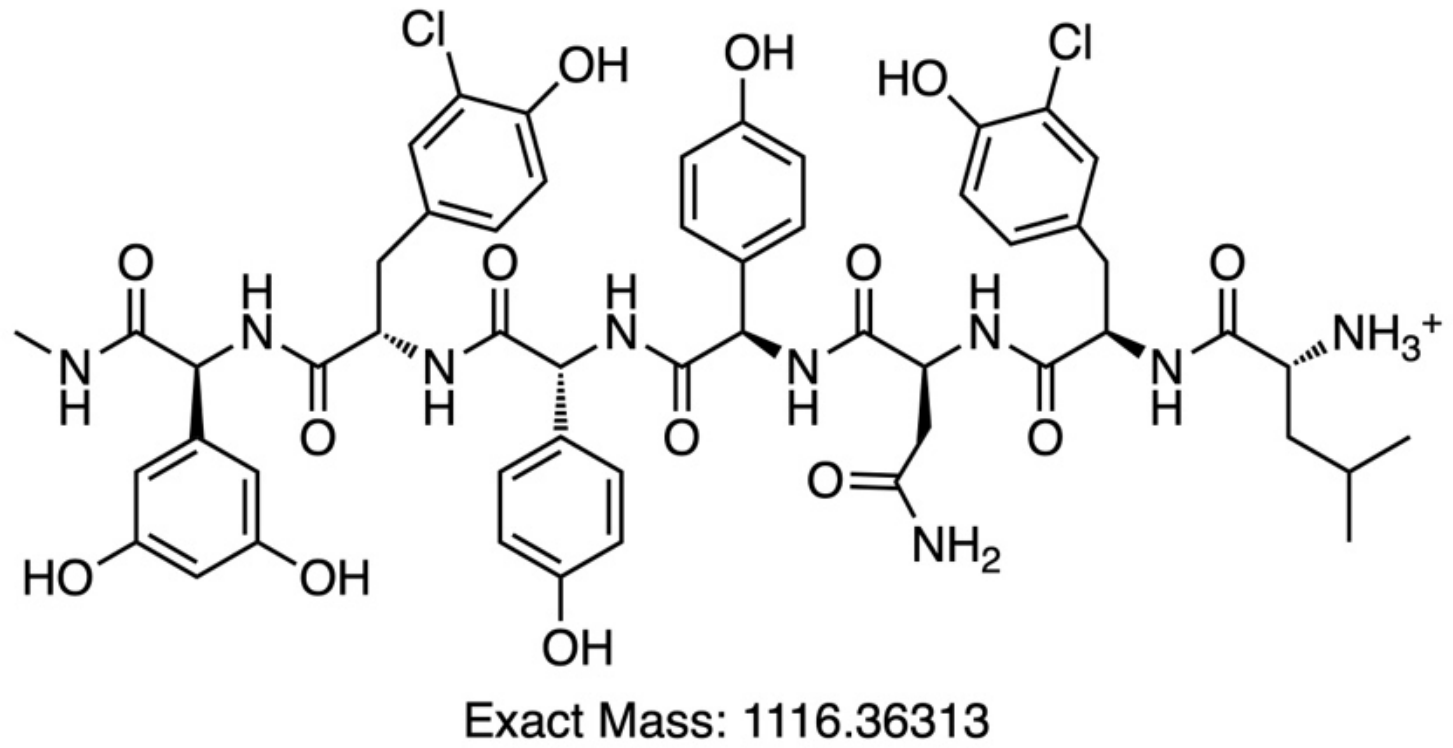

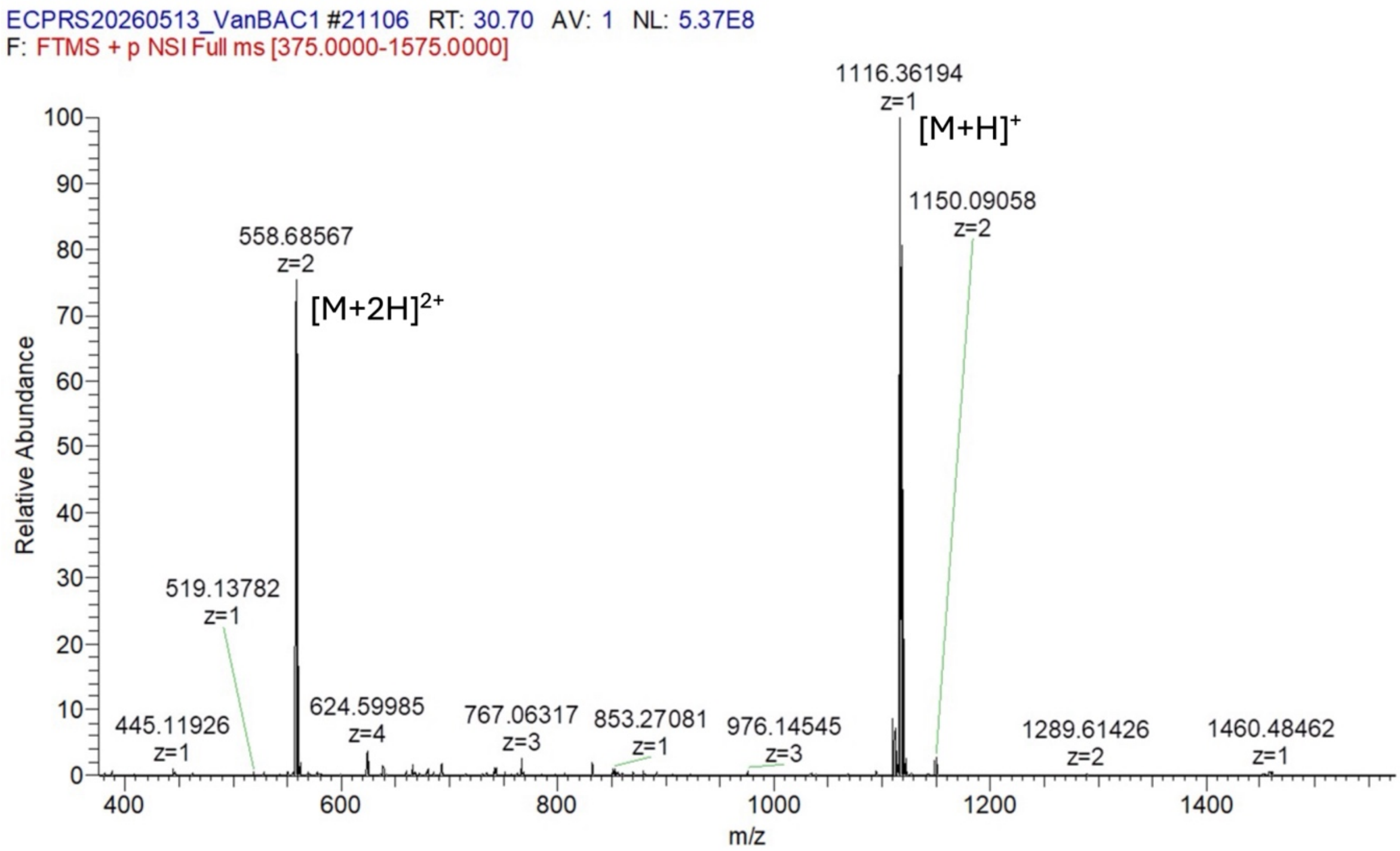

SI Figure 28. HRMS of the linear methylamide product of **5** from crosslinking reaction with $OxyB_{bal}$, $OxyA_{ris}$ and $OxyC_{cep.}$

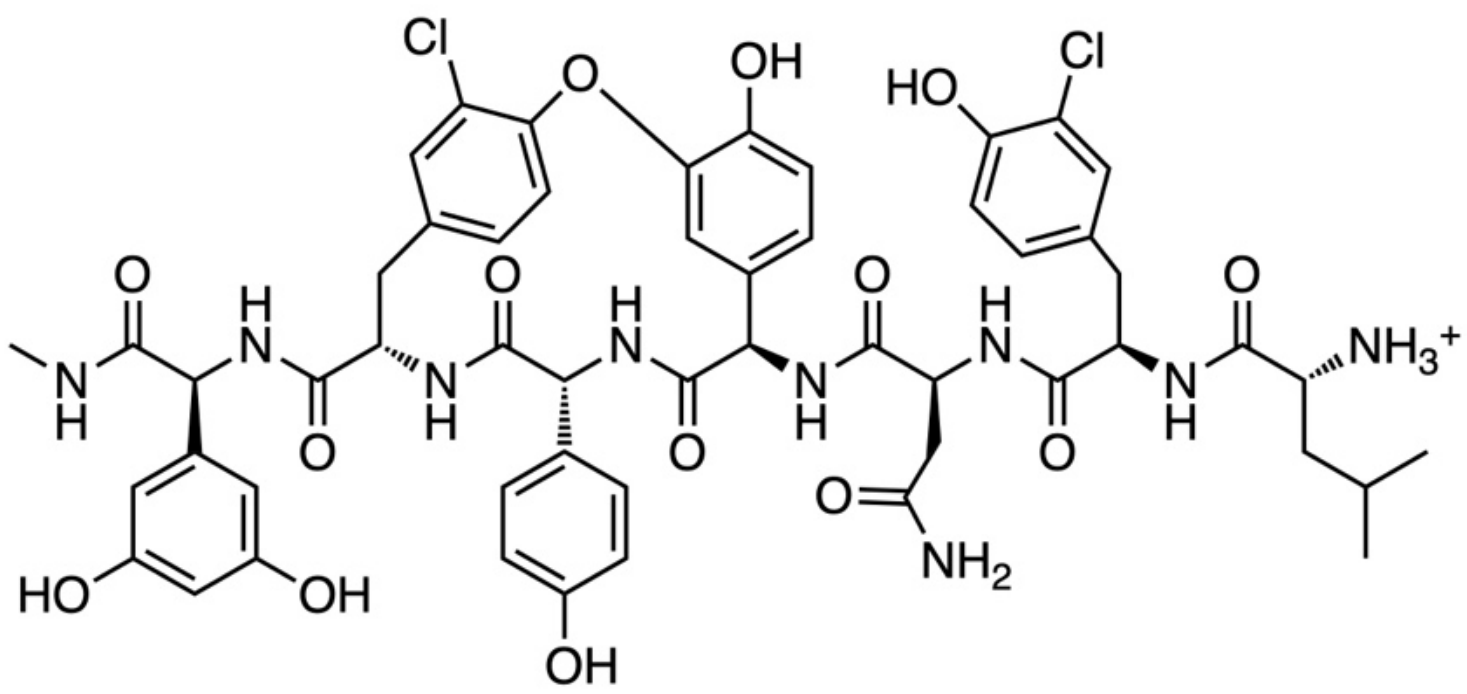

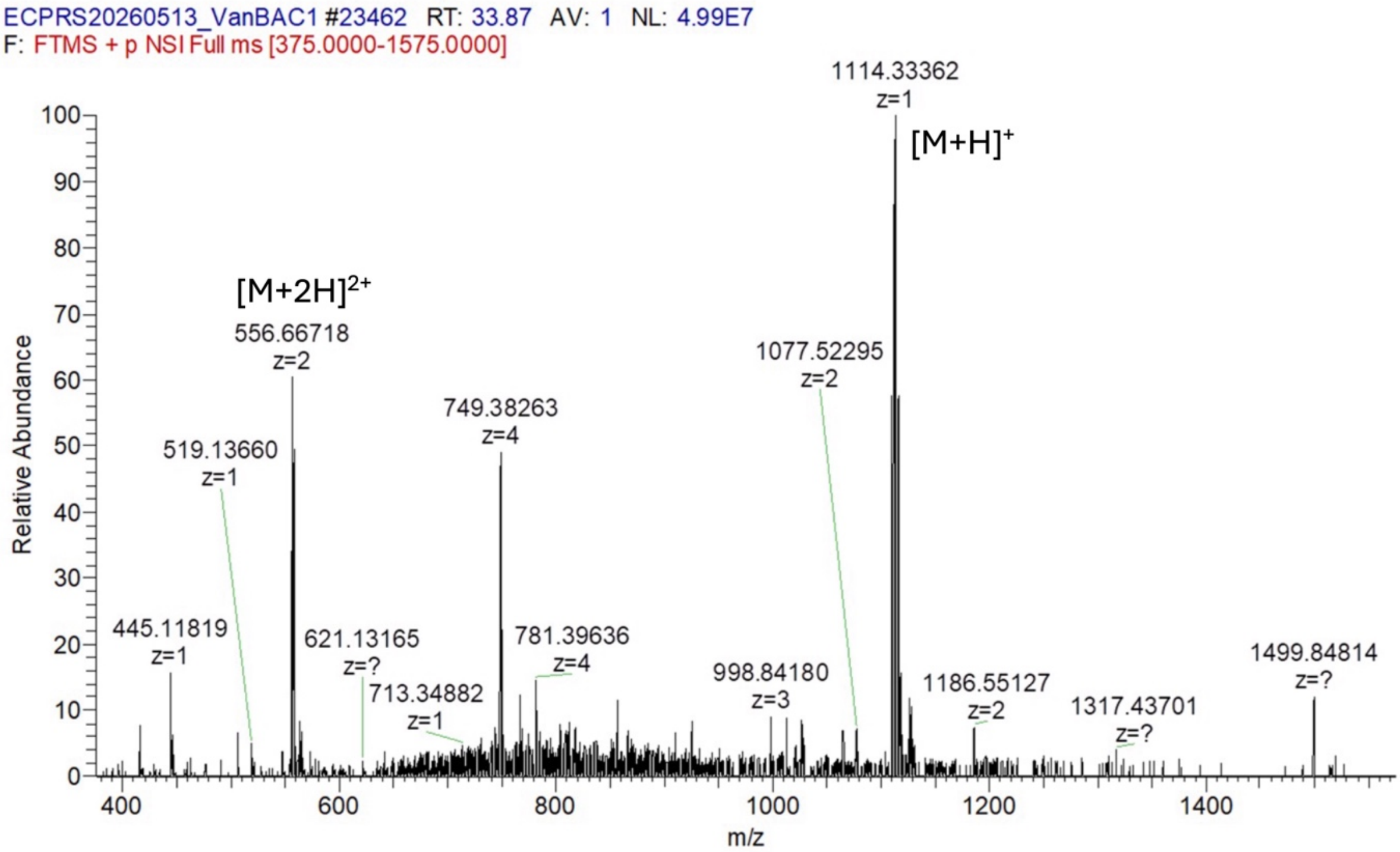

SI Figure 29. HRMS of the monocyclic methylamide product of **5** from crosslinking reaction with $OxyB_{bal}$, $OxyA_{ris}$ and $OxyC_{cep}$.

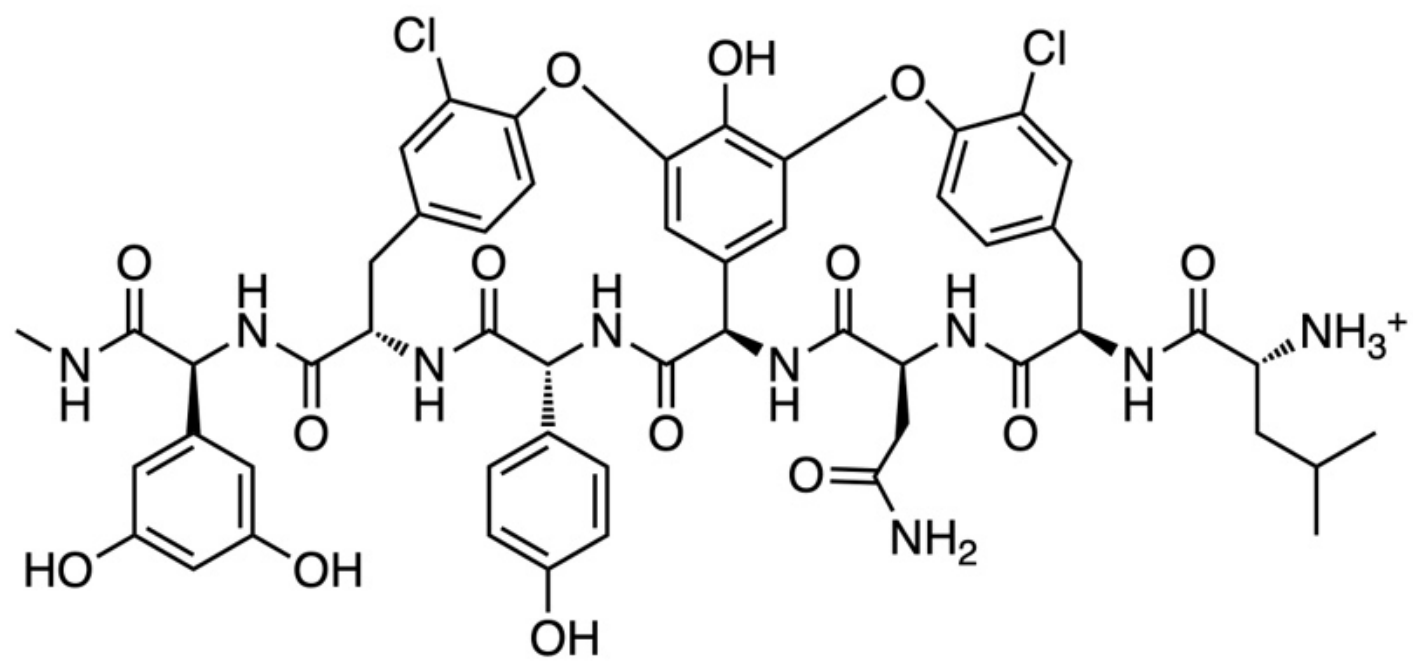

Exact Mass: 1112.33183

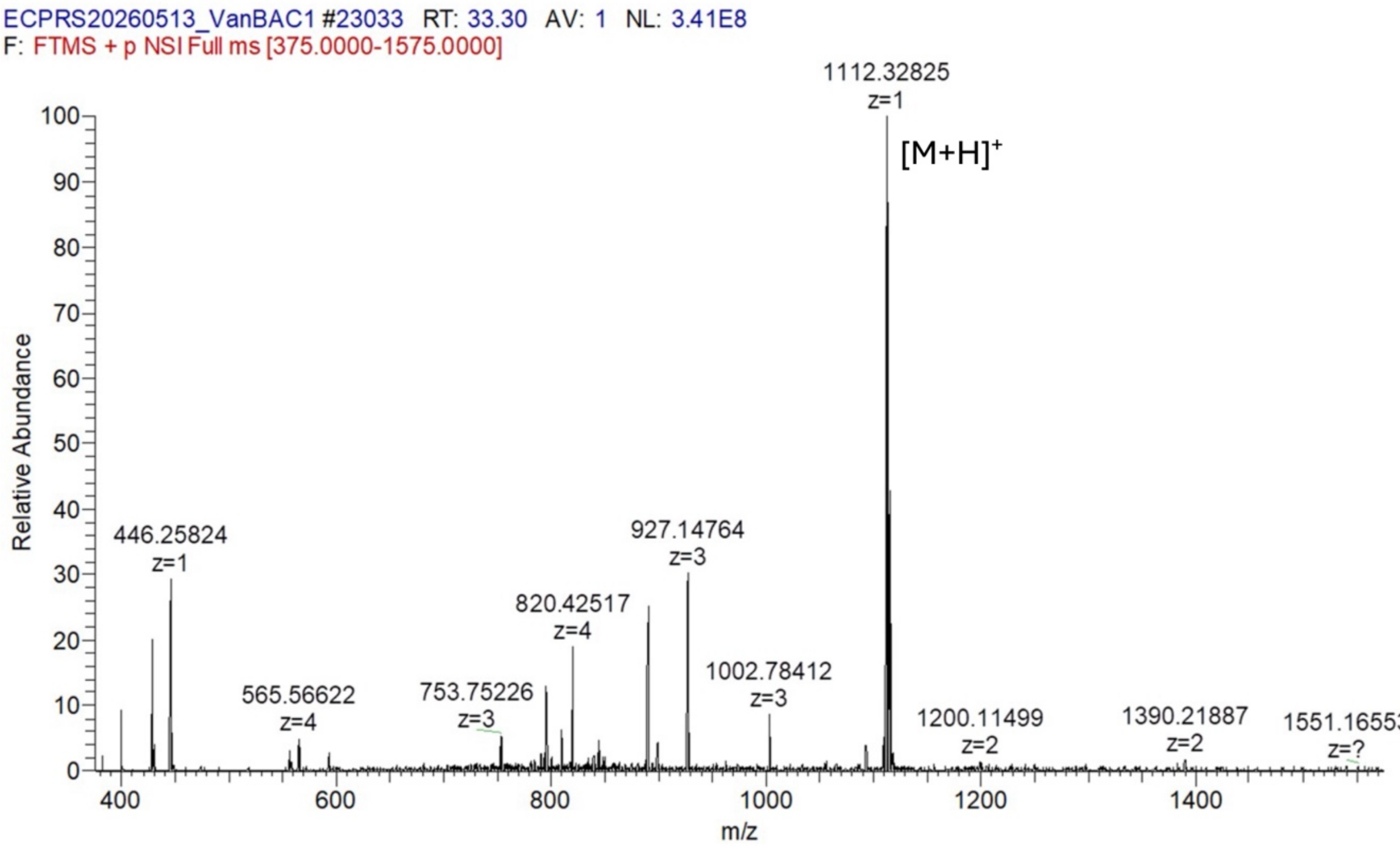

SI Figure 30. HRMS of the bicyclic methylamide product of **5** from crosslinking reaction with $OxyB_{bal}$, $OxyA_{ris}$ and $OxyC_{cep}$.

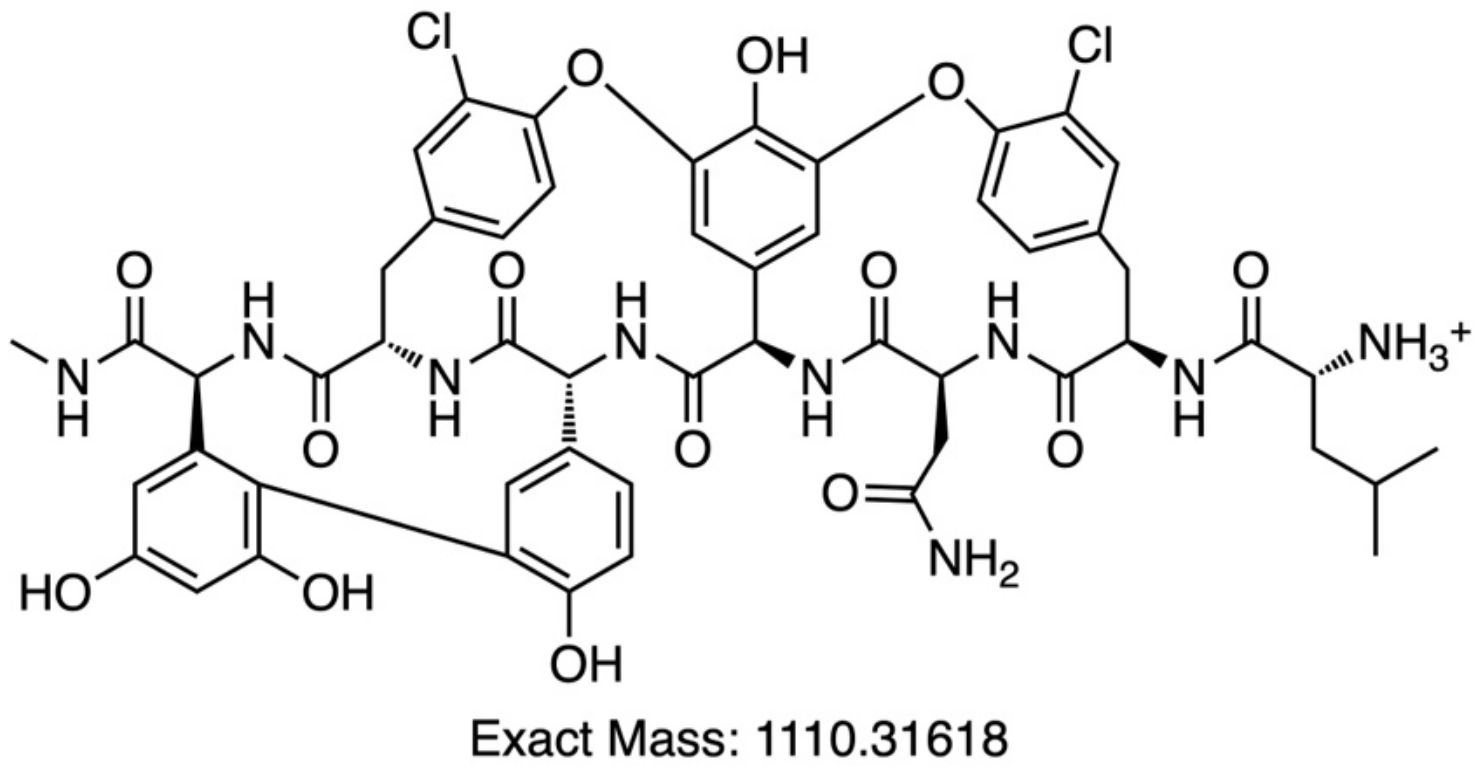

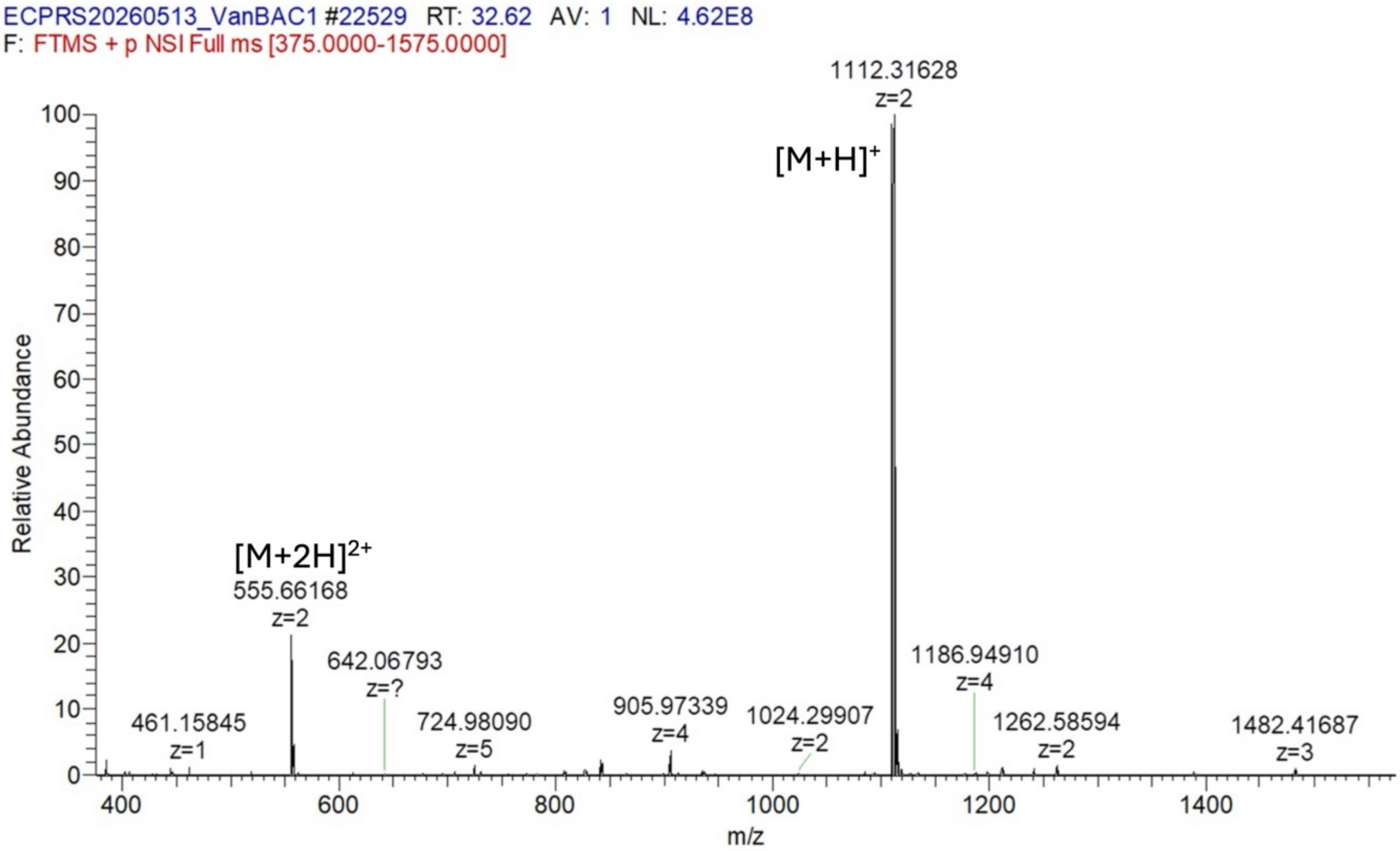

SI Figure 31. HRMS of the tricyclic methylamide product of **5** from crosslinking reaction with $OxyB_{bal}$, $OxyA_{ris}$ and $OxyC_{cep.}$

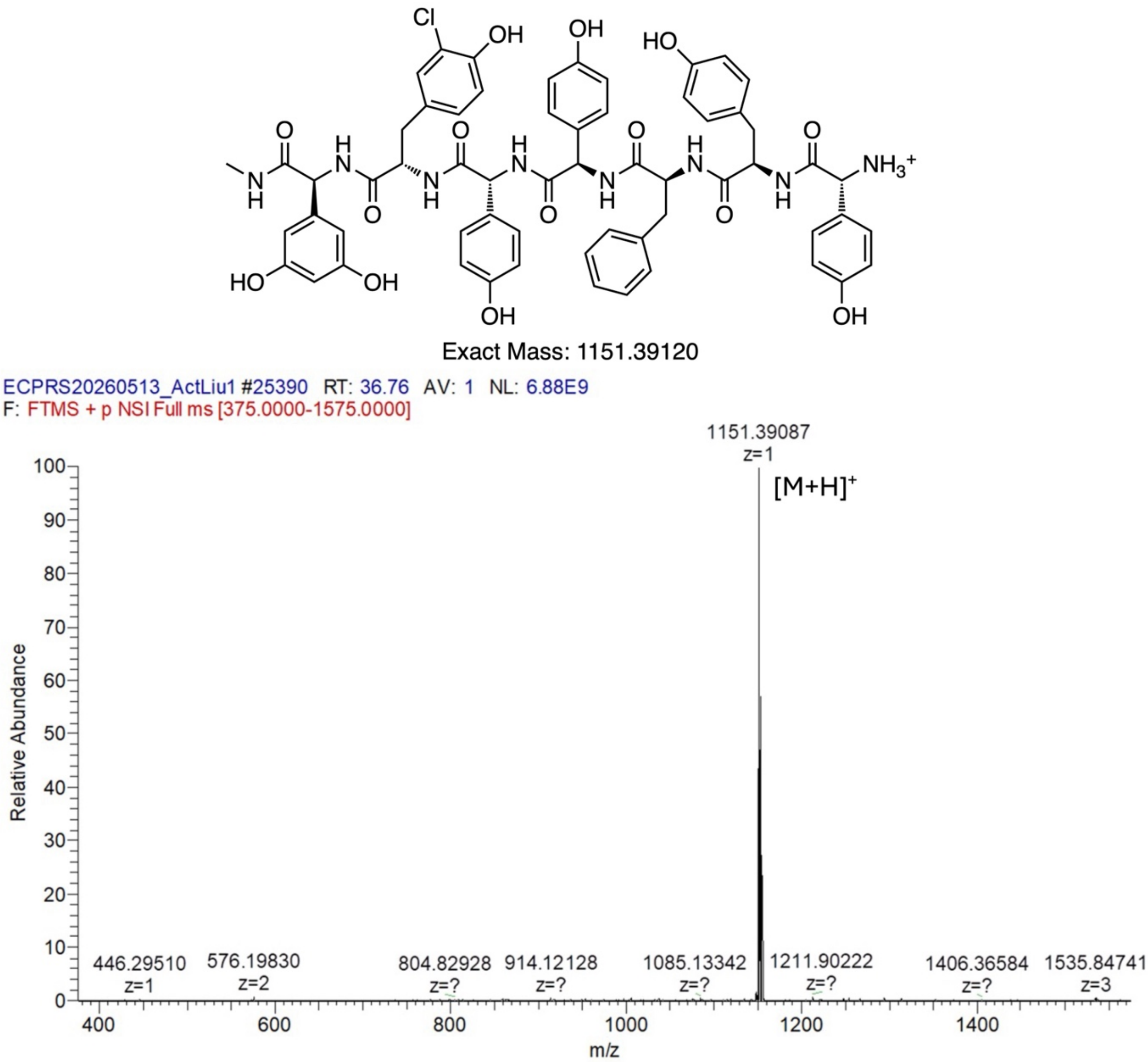

SI Figure 32. HRMS of the linear methylamide product of **2** synthesised via Method A from crosslinking reaction with $OxyB_{bal}$, $OxyA_{ris}$ and $OxyC_{cep.}$

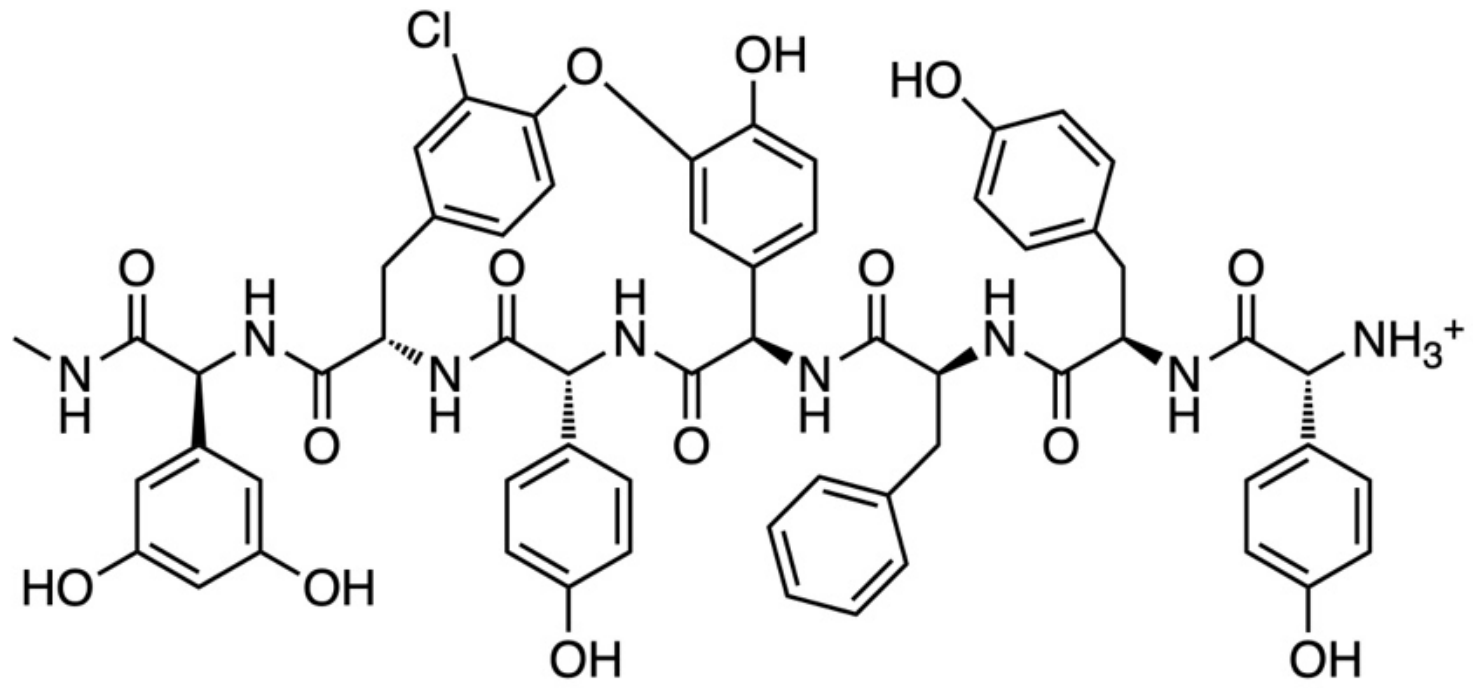

Exact Mass: 1149.37555

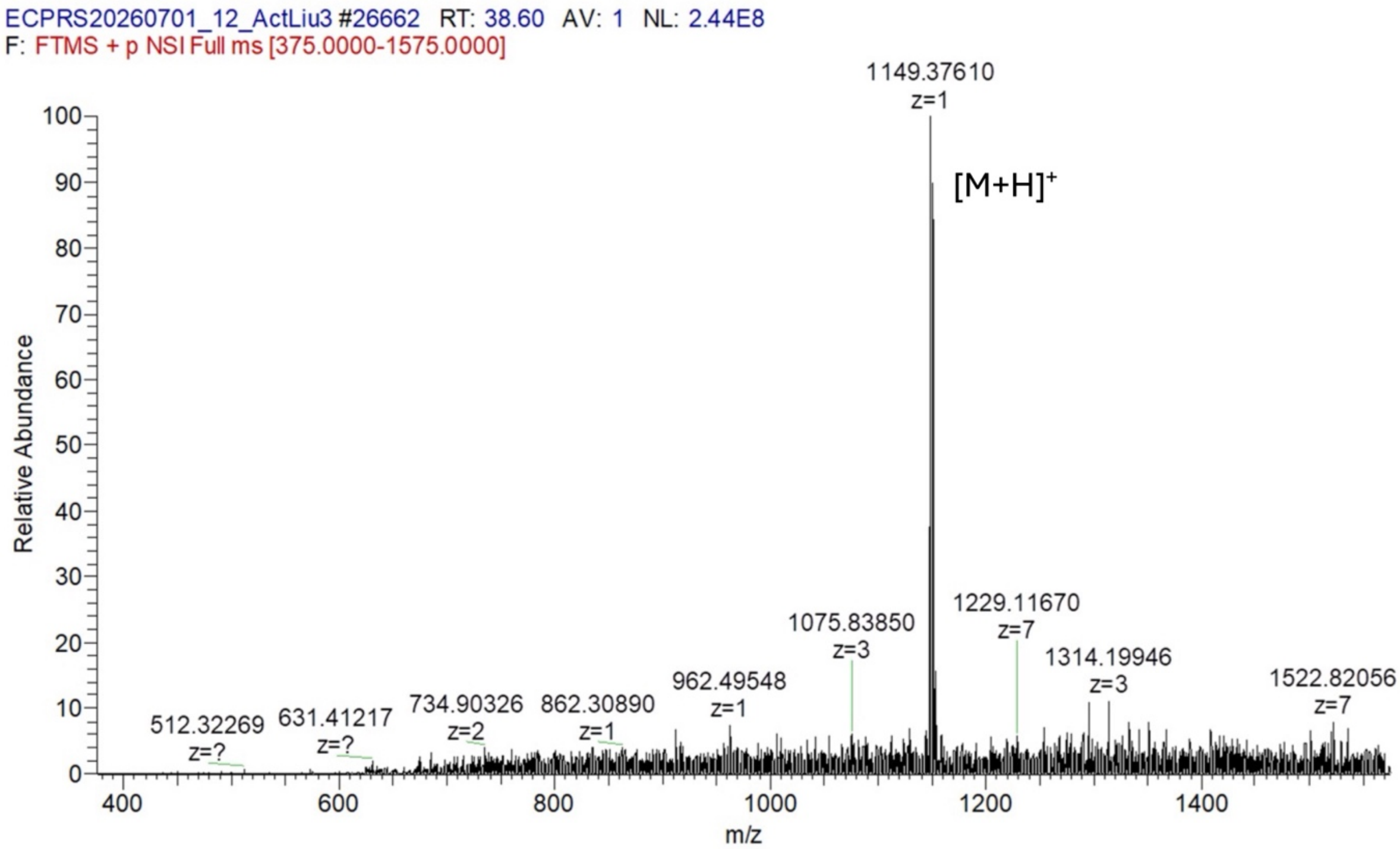

SI Figure 33. HRMS of the monocyclic methylamide product of **2** synthesised via Method A from crosslinking reaction with $OxyB_{bal}$, $OxyA_{ris}$ and $OxyC_{cep.}$

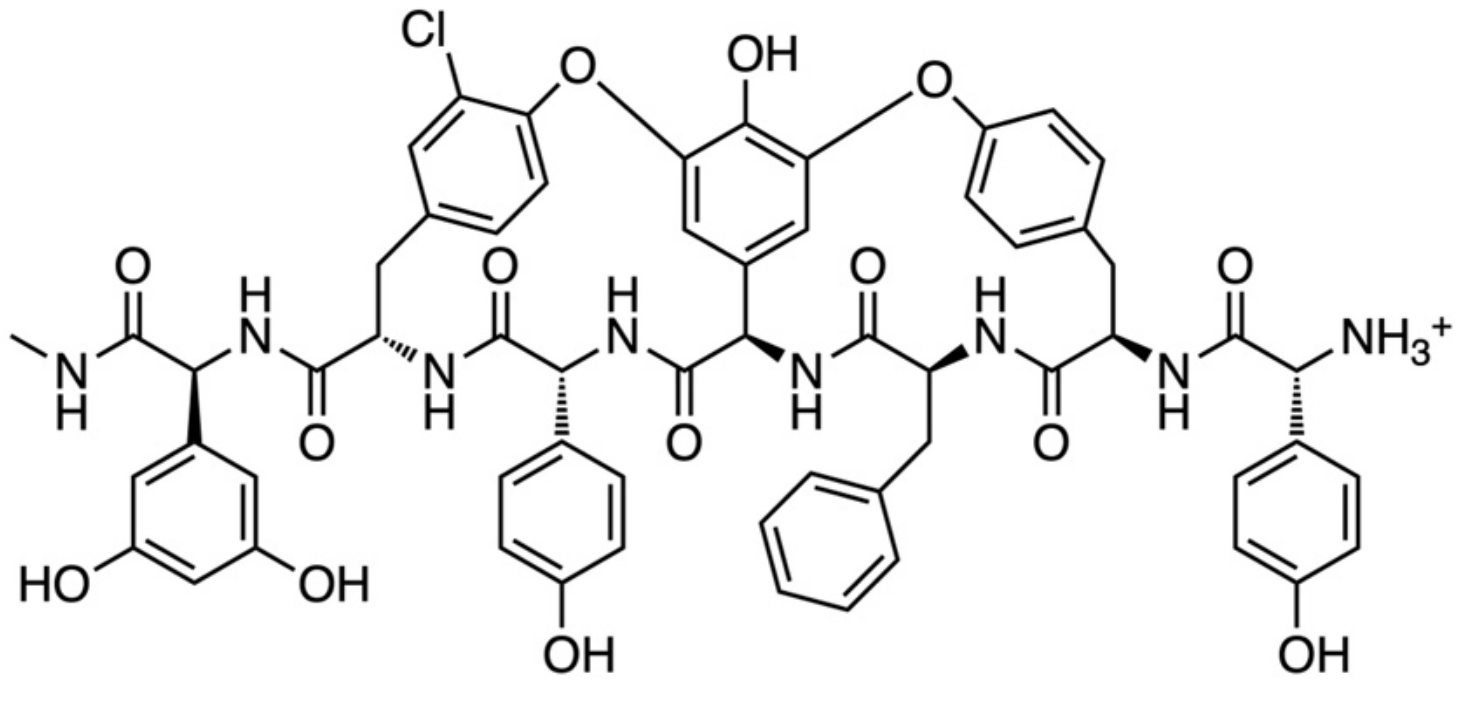

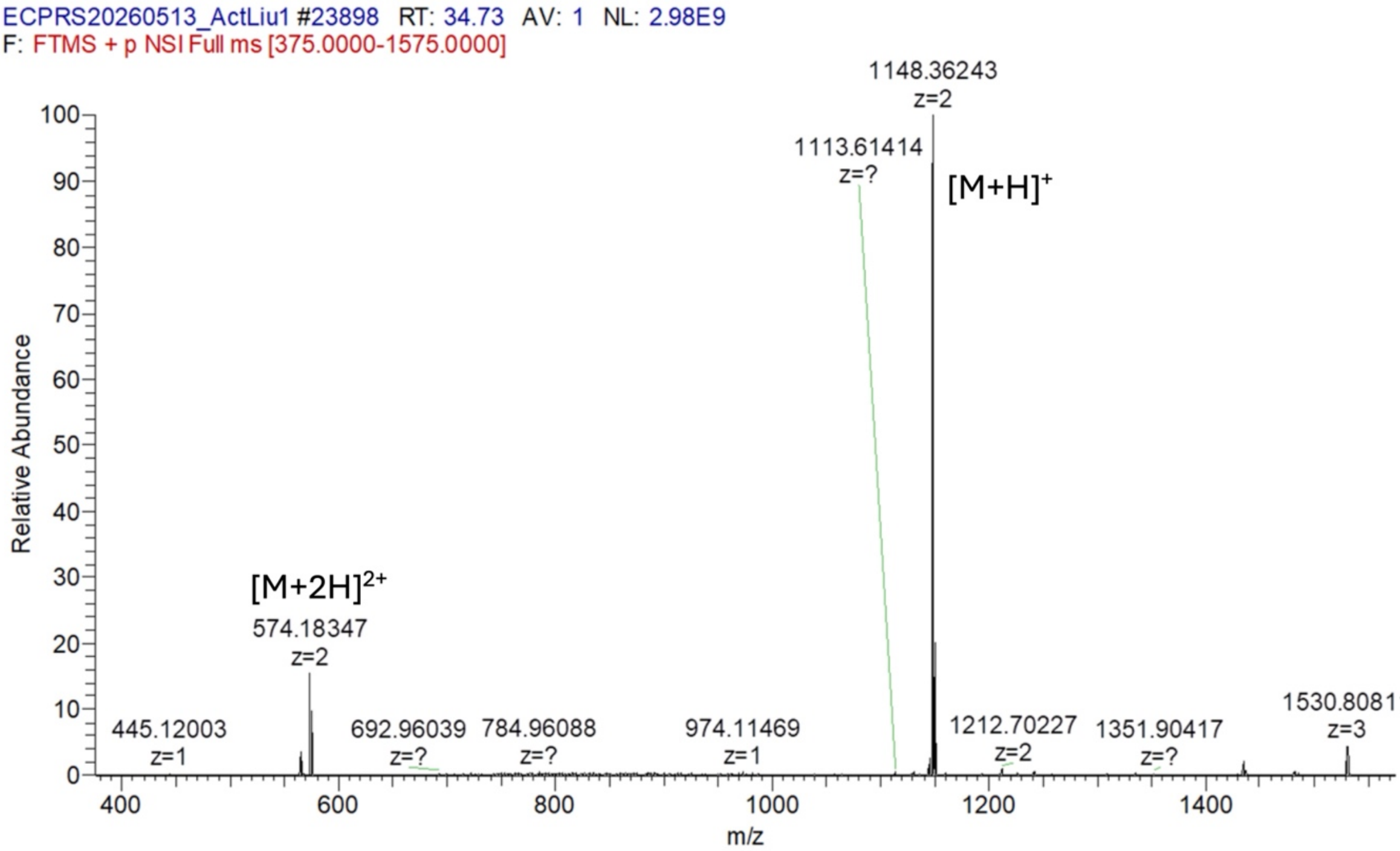

SI Figure 34. HRMS of the bicyclic methylamide product of **2** synthesised via Method A from crosslinking reaction with $OxyB_{bal}$, $OxyA_{ris}$ and $OxyC_{cep.}$

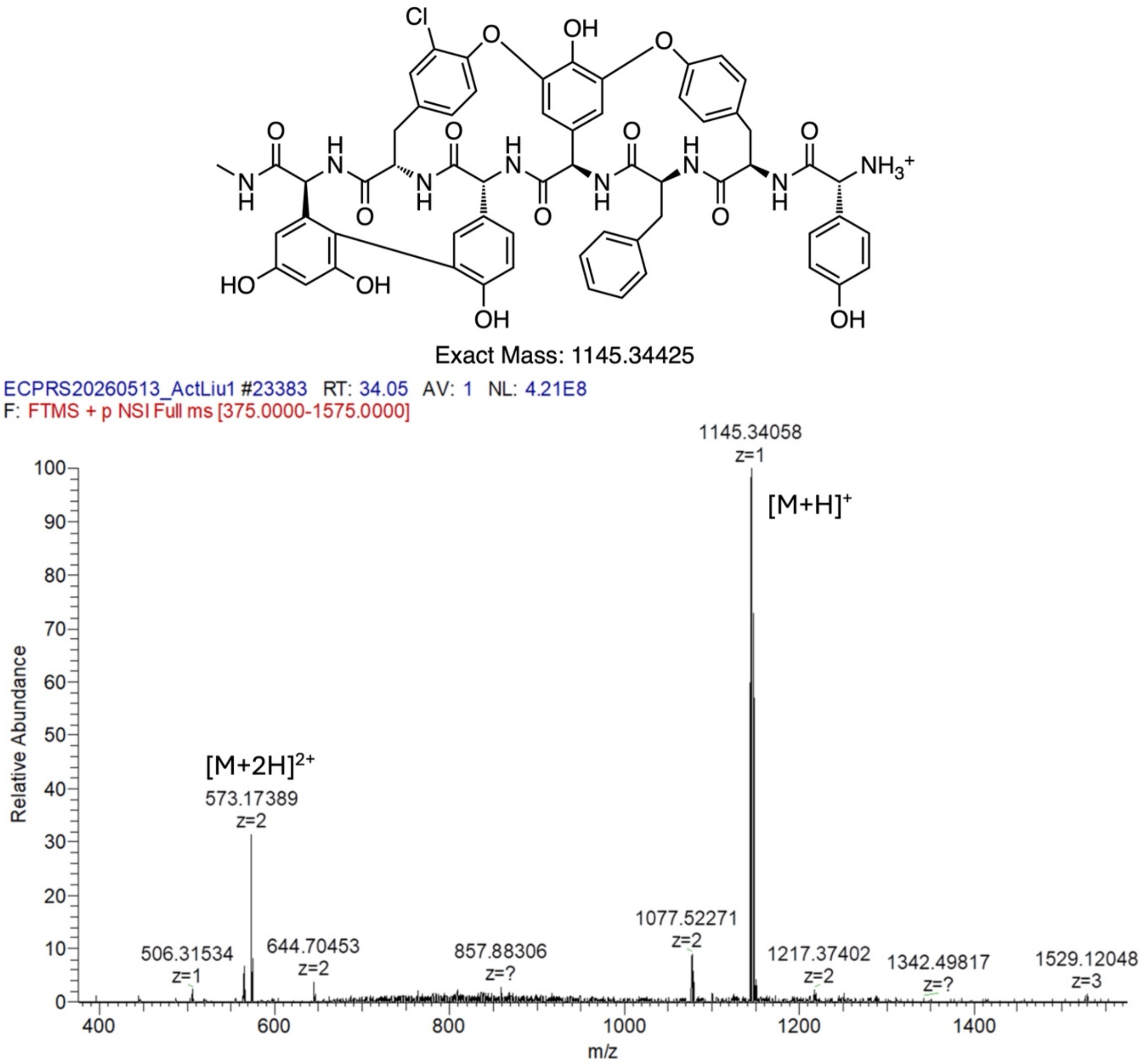

SI Figure 35. HRMS of the tricyclic methylamide product of **2** synthesised via Method A from crosslinking reaction with $OxyB_{bal}$, $OxyA_{ris}$ and $OxyC_{cep.}$

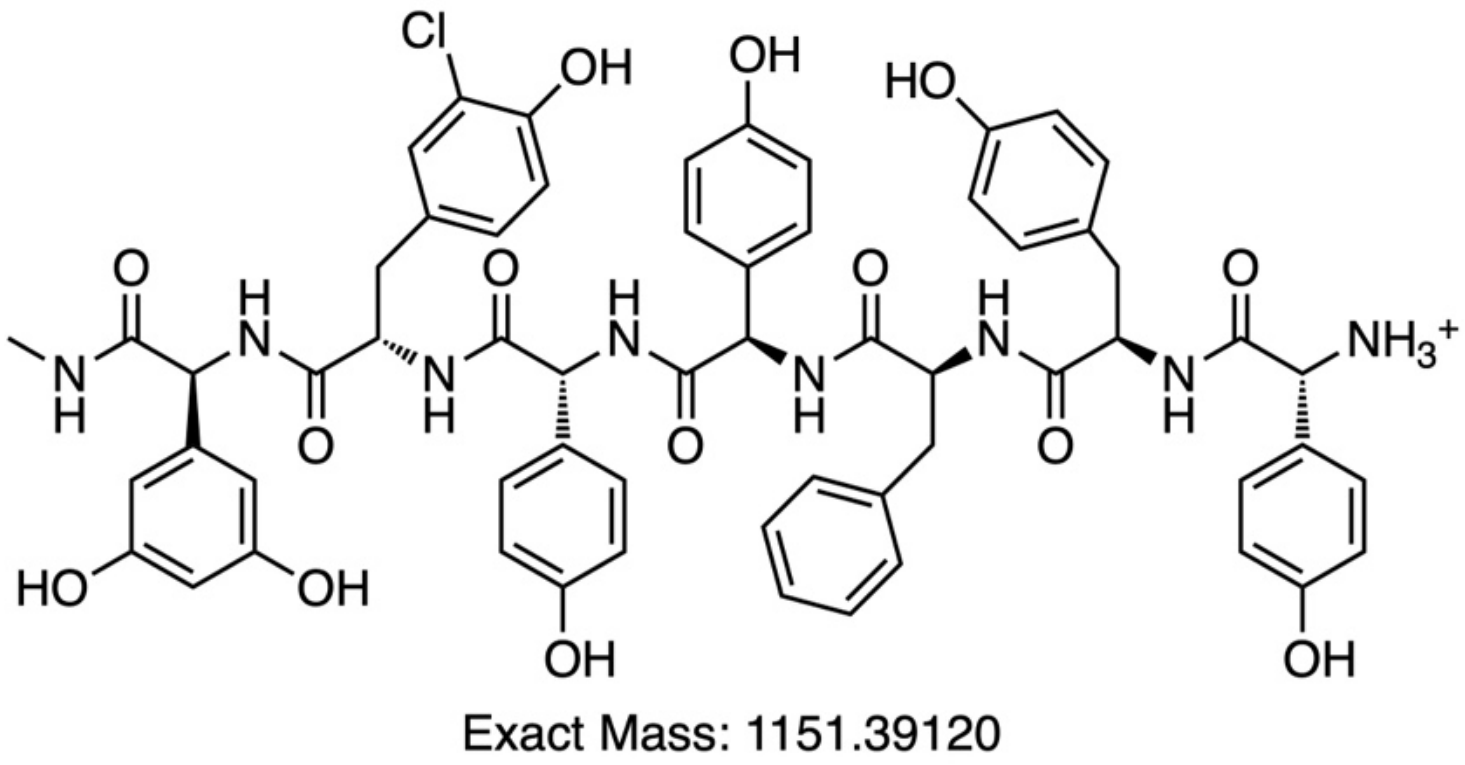

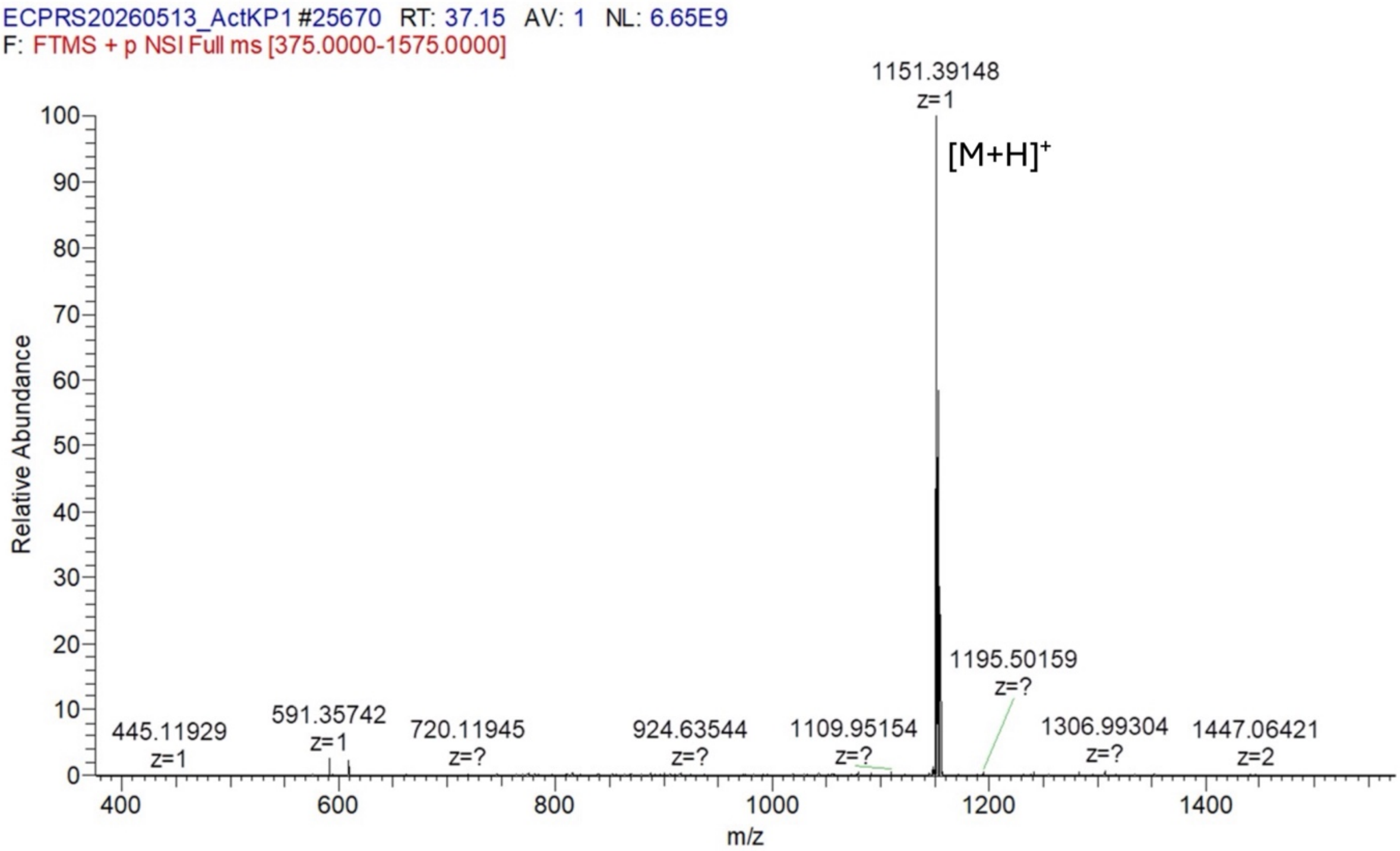

SI Figure 36. HRMS of the linear methylamide product of **2** synthesised via Method C from crosslinking reaction with $OxyB_{bal}$, $OxyA_{ris}$ and $OxyC_{cep}$.

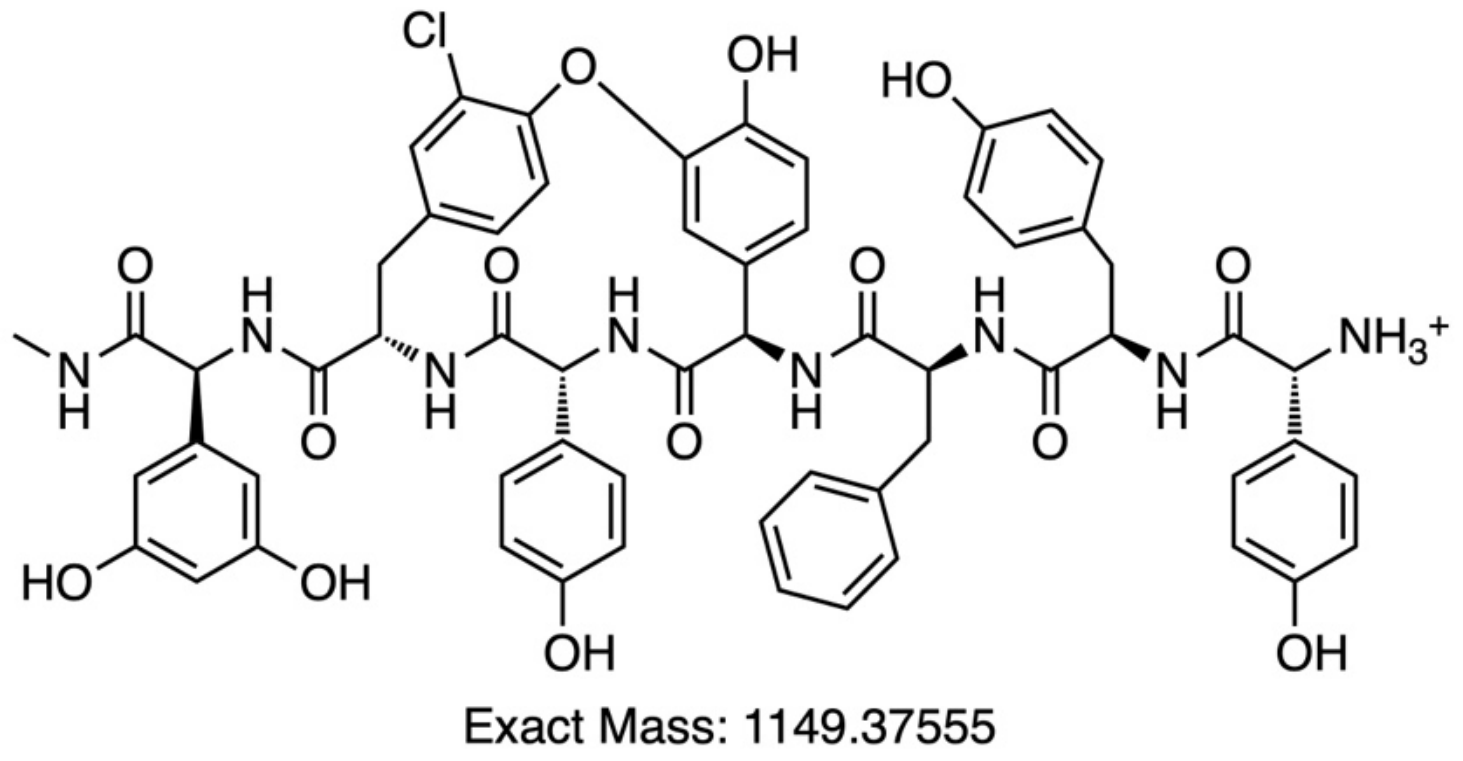

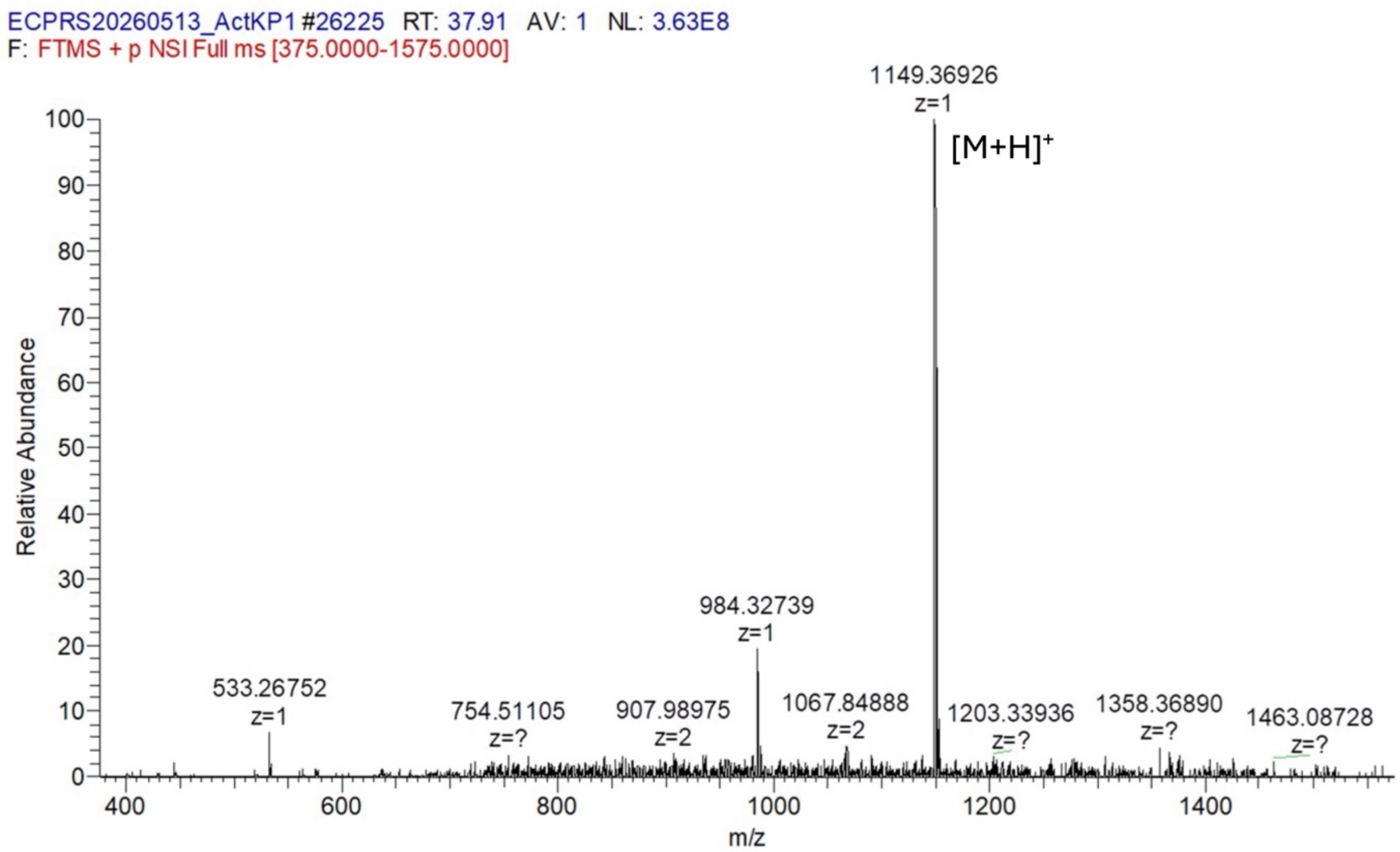

SI Figure 37. HRMS of the monocyclic methylamide product of **2** synthesised via Method C from crosslinking reaction with $OxyB_{bal}$, $OxyA_{ris}$ and $OxyC_{cep.}$

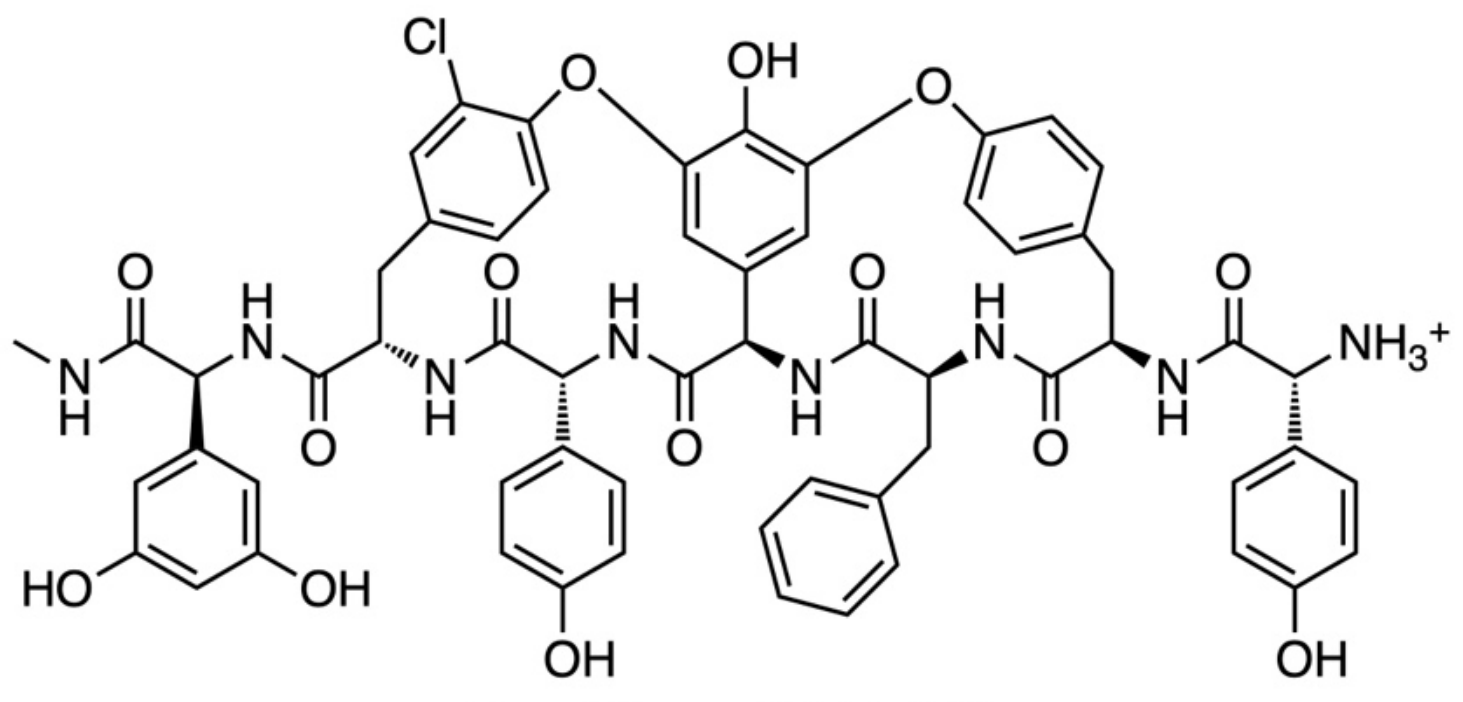

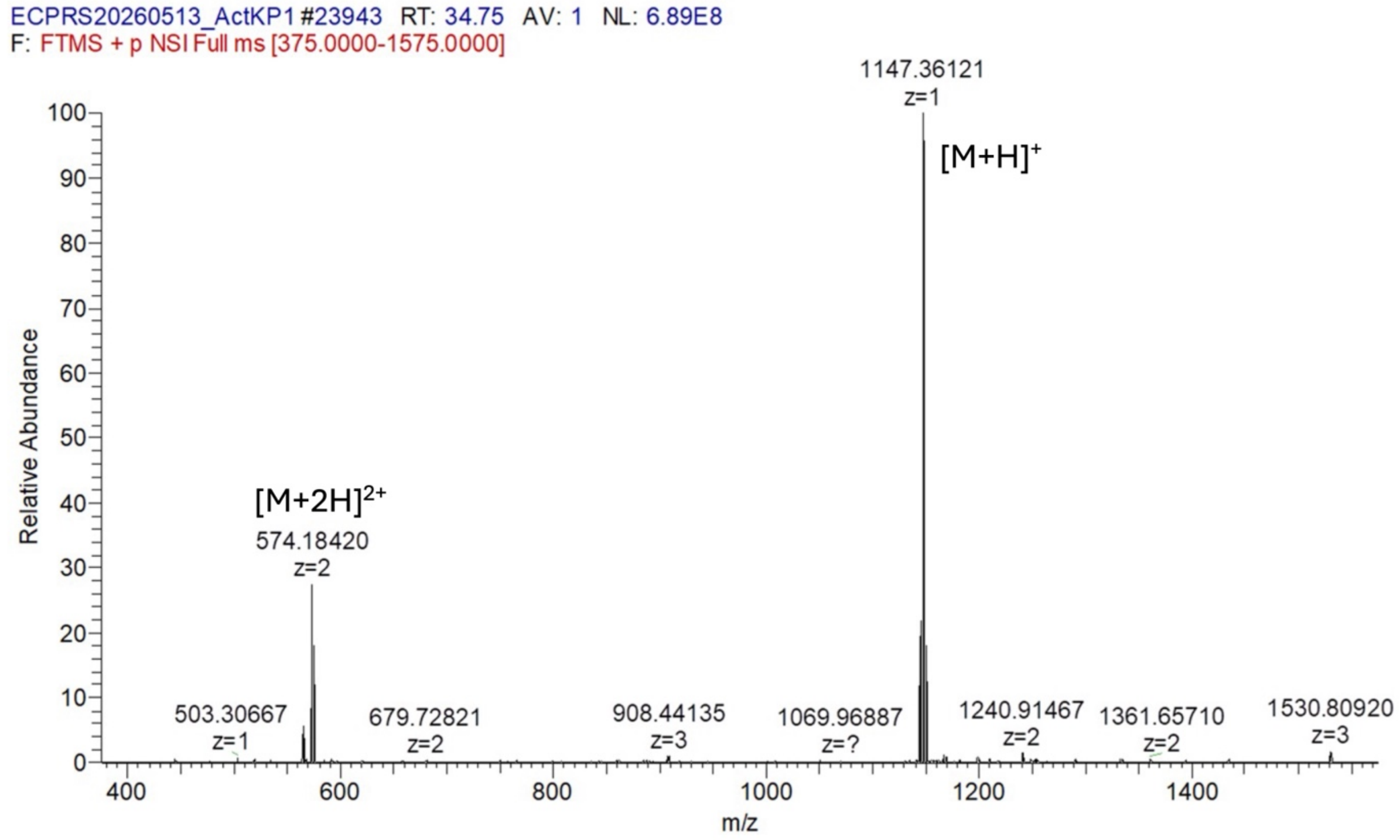

SI Figure 38. HRMS of the bicyclic methylamide product of **2** synthesised via Method C from crosslinking reaction with $OxyB_{bal}$, $OxyA_{ris}$ and $OxyC_{cep.}$

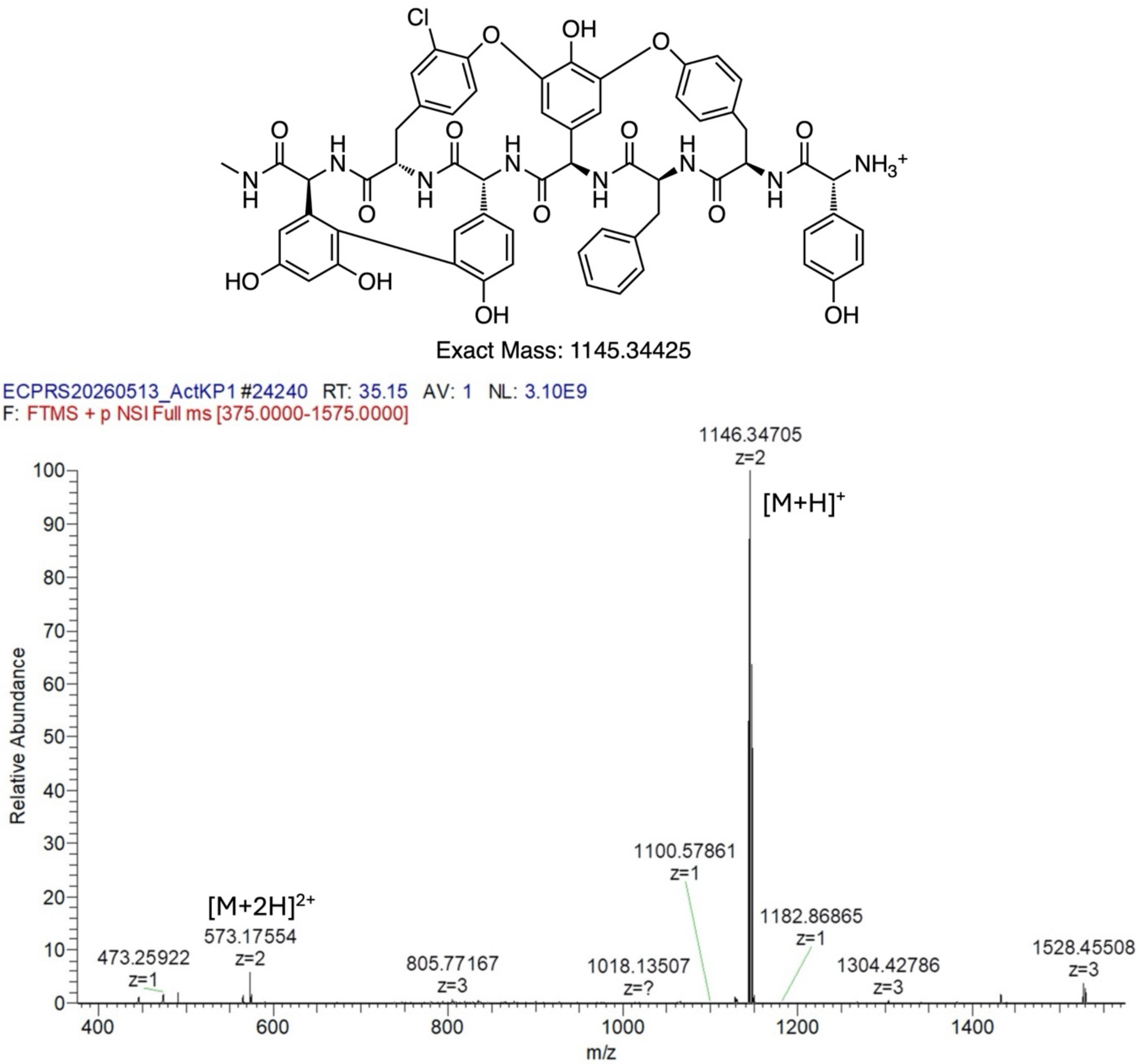

SI Figure 39. HRMS of the tricyclic methylamide product of **2** synthesised via Method C from crosslinking reaction with $OxyB_{bal}$, $OxyA_{ris}$ and $OxyC_{cep.}$

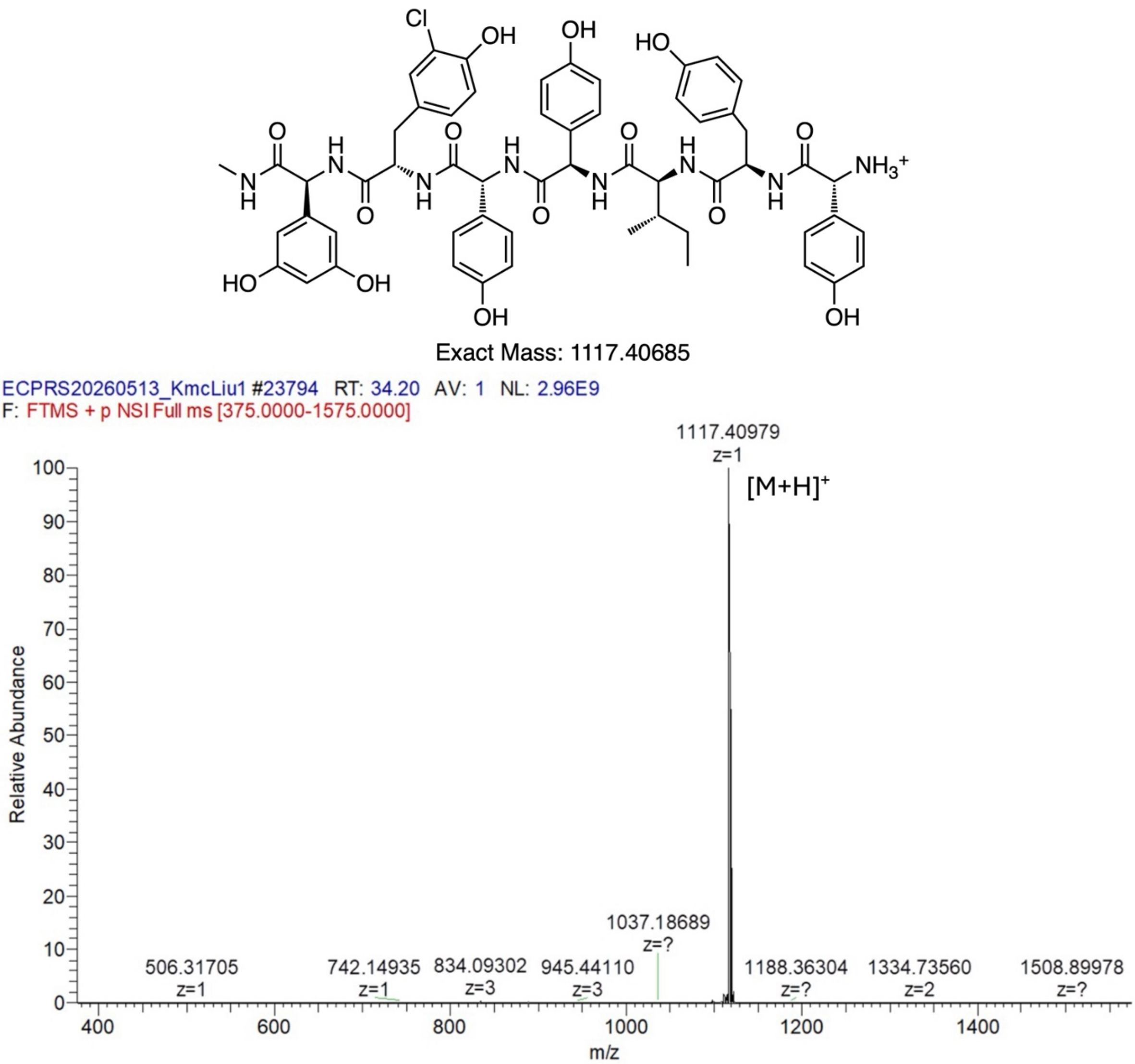

SI Figure 40. HRMS of the linear methylamide product of **3** synthesised via Method A from crosslinking reaction with $OxyB_{bal}$, $OxyA_{ris}$ and $OxyC_{cep.}$

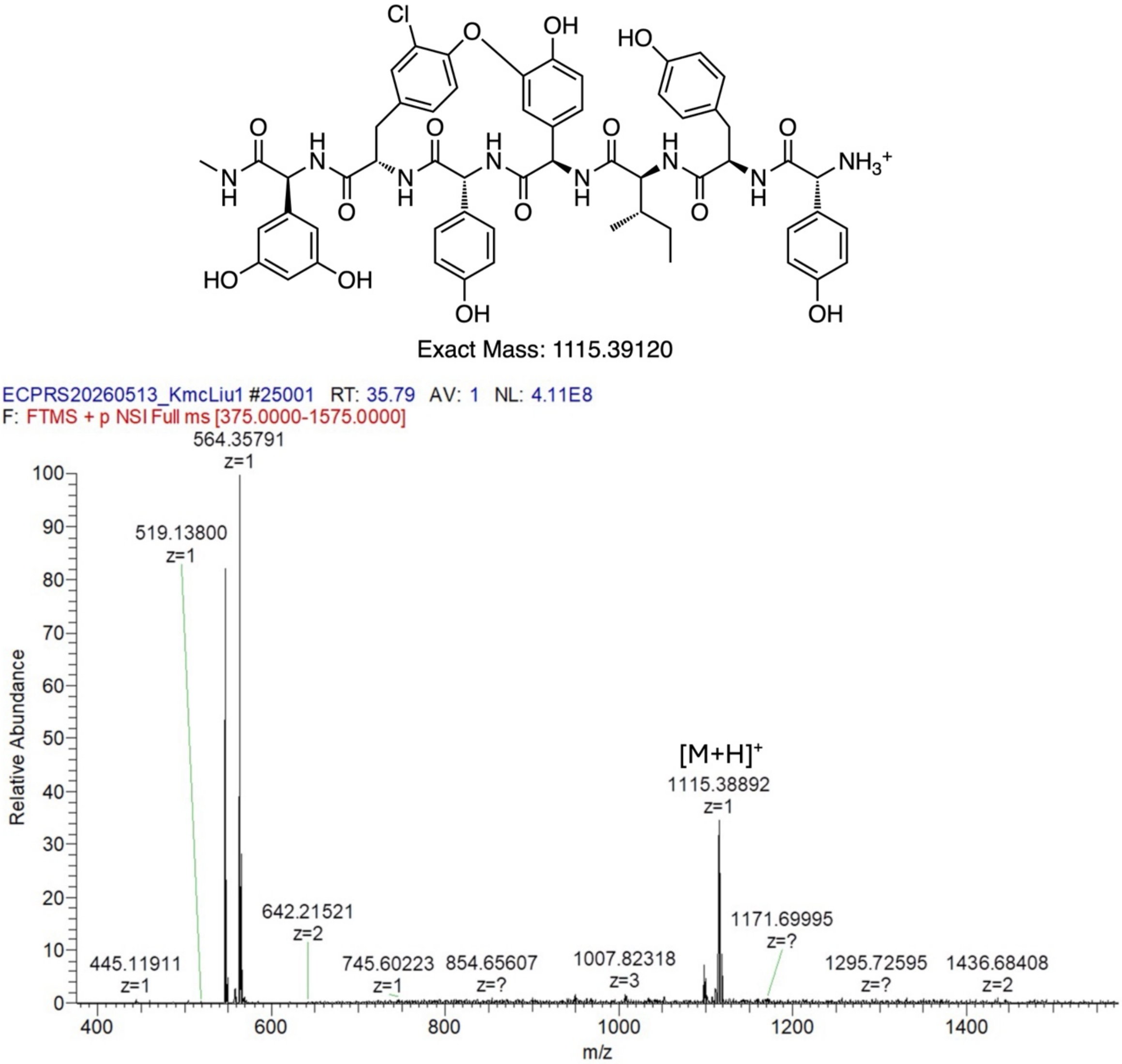

SI Figure 41. HRMS of the monocyclic methylamide product of **3** synthesised via Method A from crosslinking reaction with $OxyB_{bal}$, $OxyA_{ris}$ and $OxyC_{cep}$.

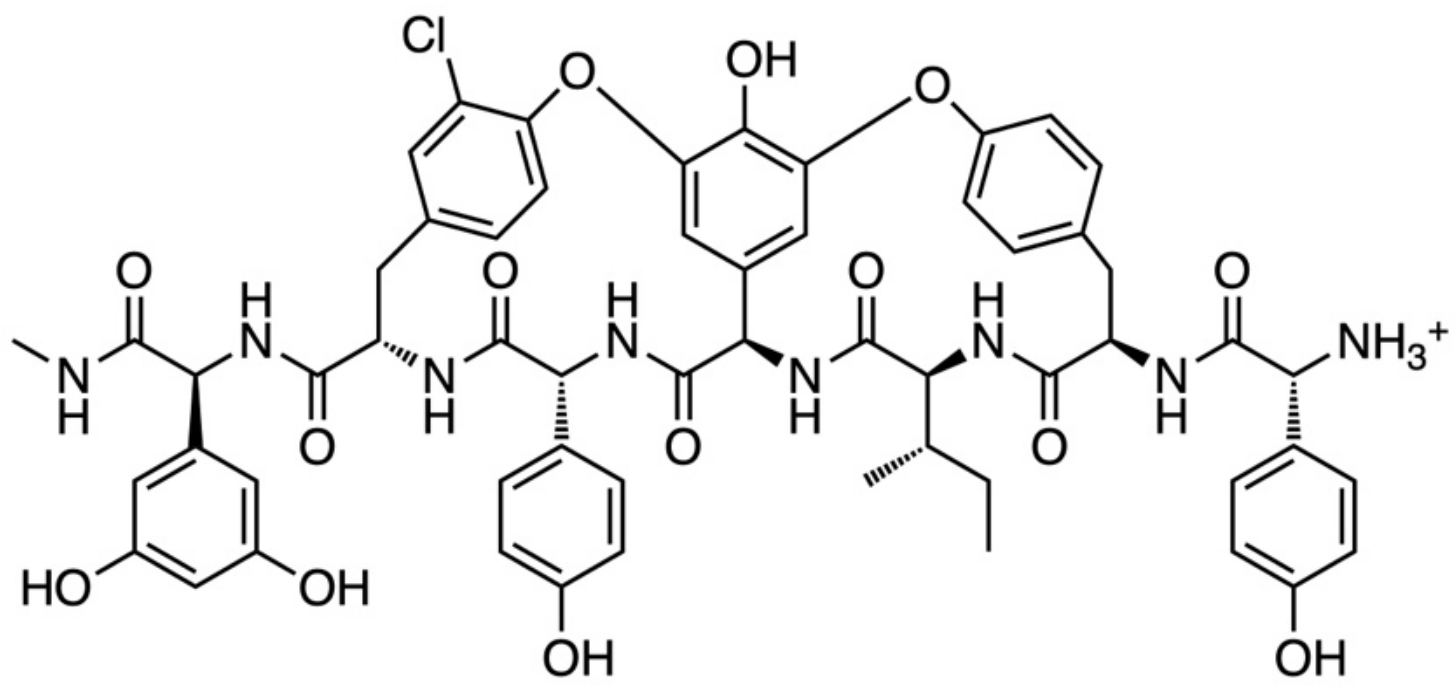

Exact Mass: 1113.37555

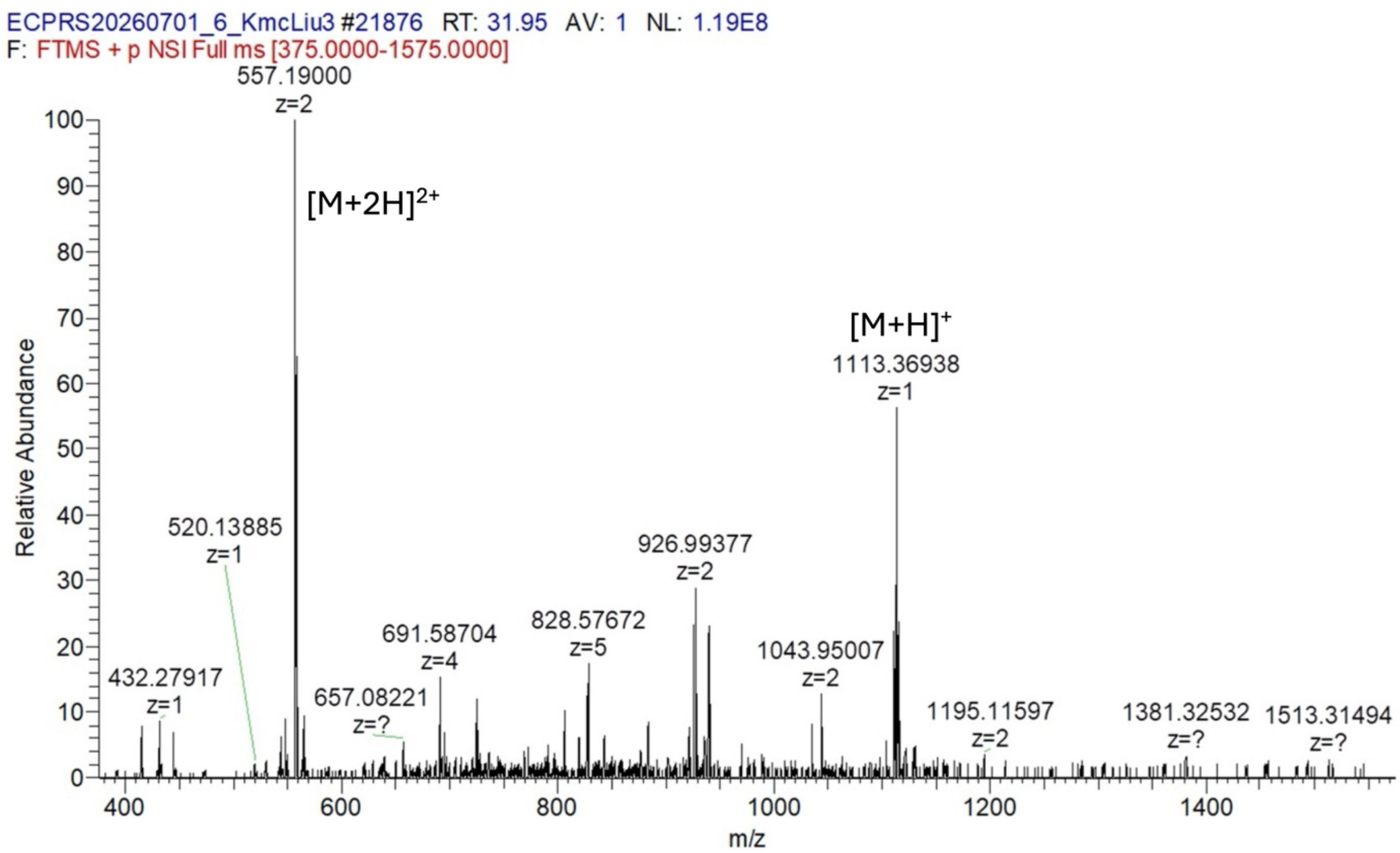

SI Figure 42. HRMS of the bicyclic methylamide product of **3** synthesised via Method A from crosslinking reaction with $OxyB_{bal}$, $OxyA_{ris}$ and $OxyC_{cep.}$

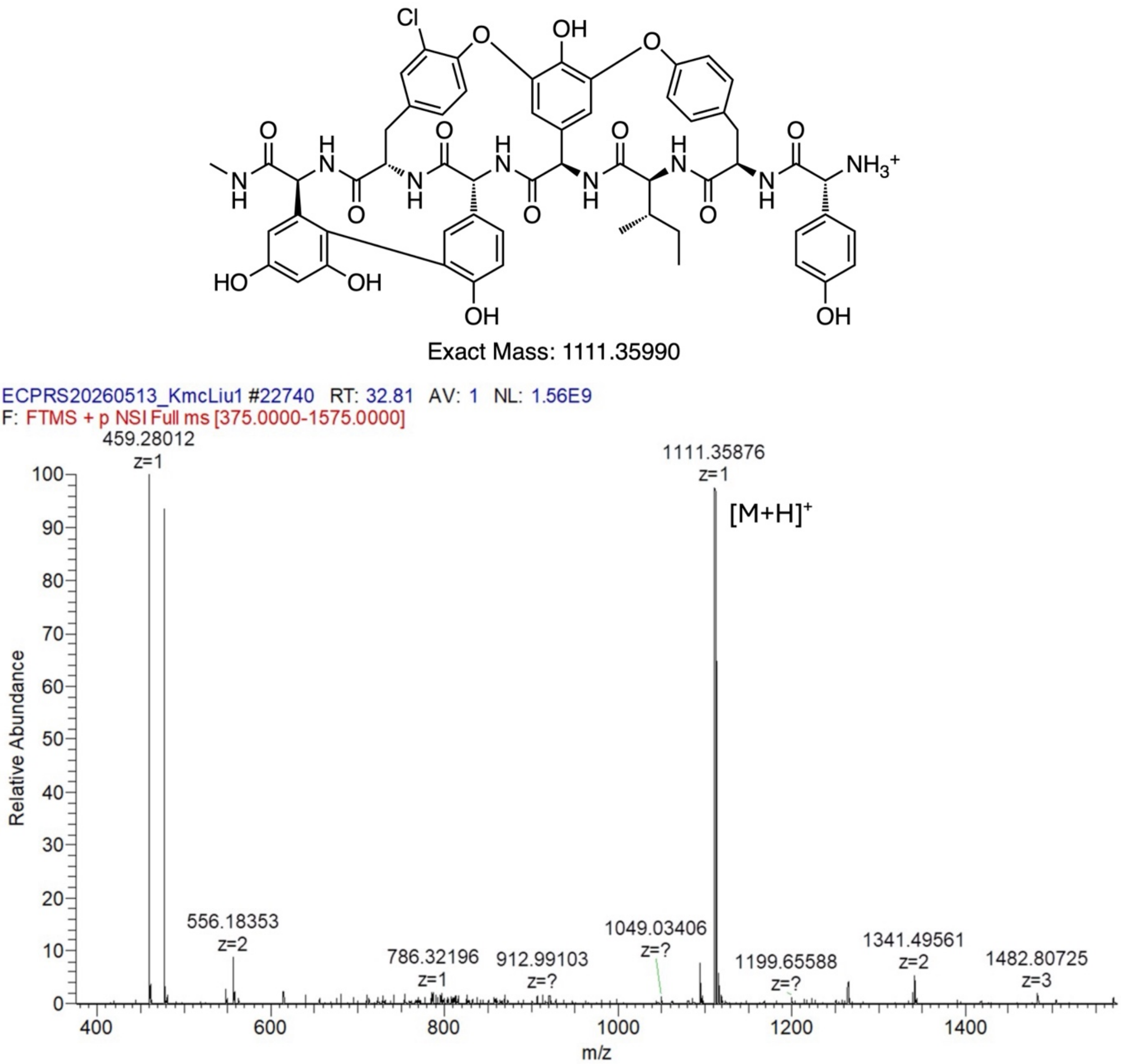

SI Figure 43. HRMS of the tricyclic methylamide product of **3** synthesised via Method A from crosslinking reaction with $OxyB_{bal}$, $OxyA_{ris}$ and $OxyC_{cep.}$

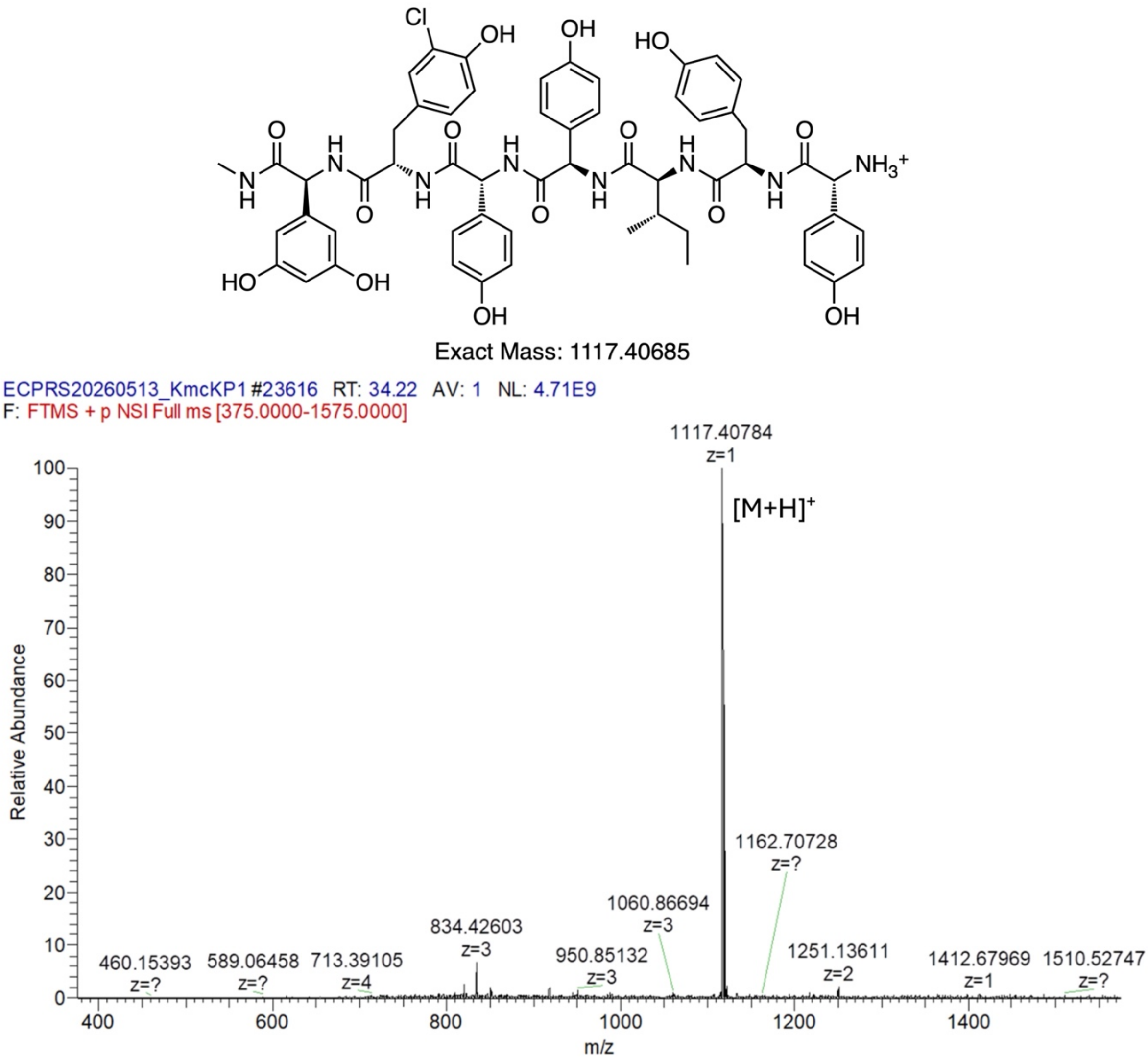

SI Figure 44. HRMS of the linear methylamide product of **3** synthesised via Method C from crosslinking reaction with $OxyB_{bal}$, $OxyA_{ris}$ and $OxyC_{cep.}$

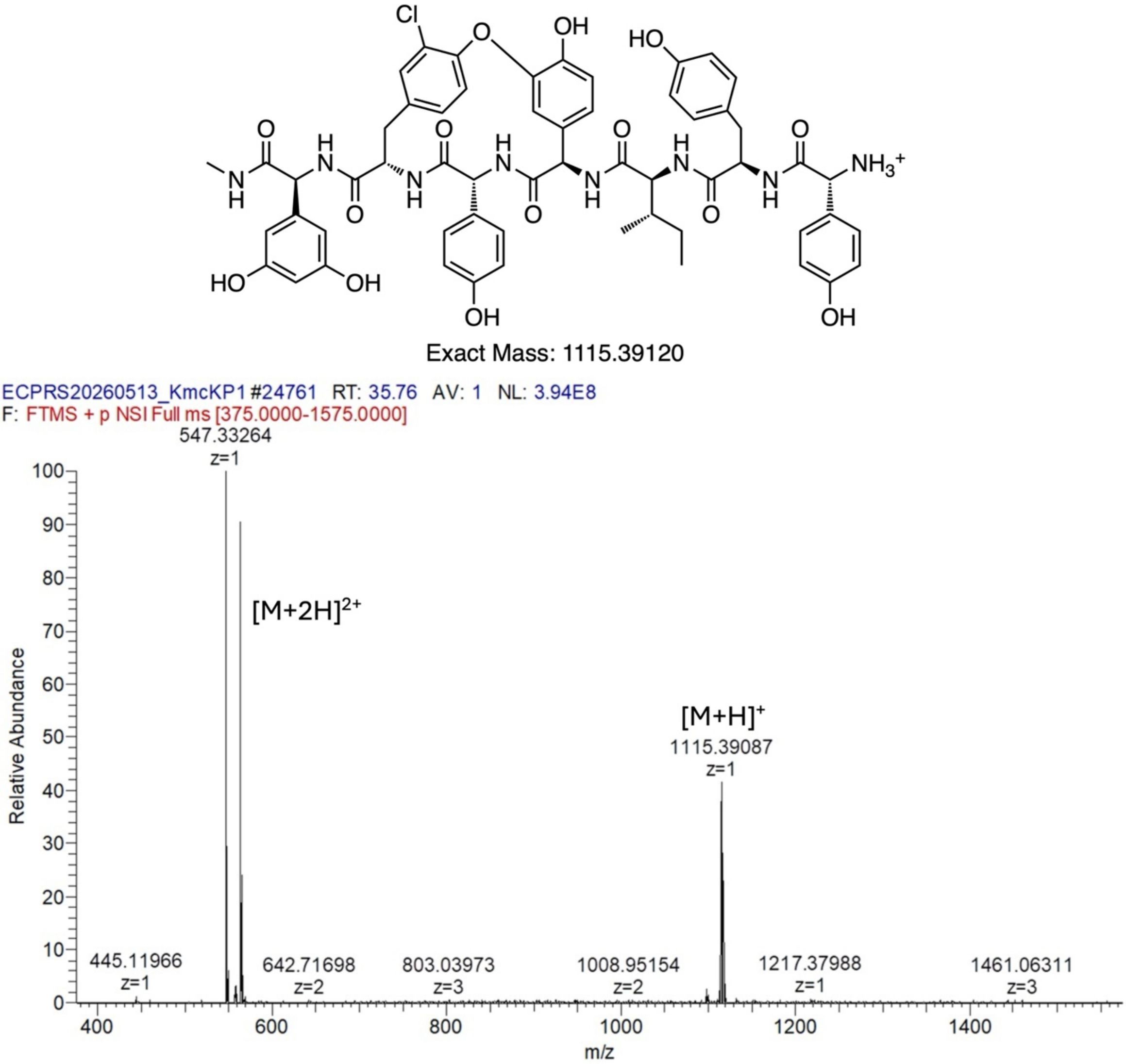

SI Figure 45. HRMS of the monocyclic methylamide product of **3** synthesised via Method C from crosslinking reaction with OxyB$_{bal}$, OxyA$_{ris}$ and OxyC$_{cep.}$

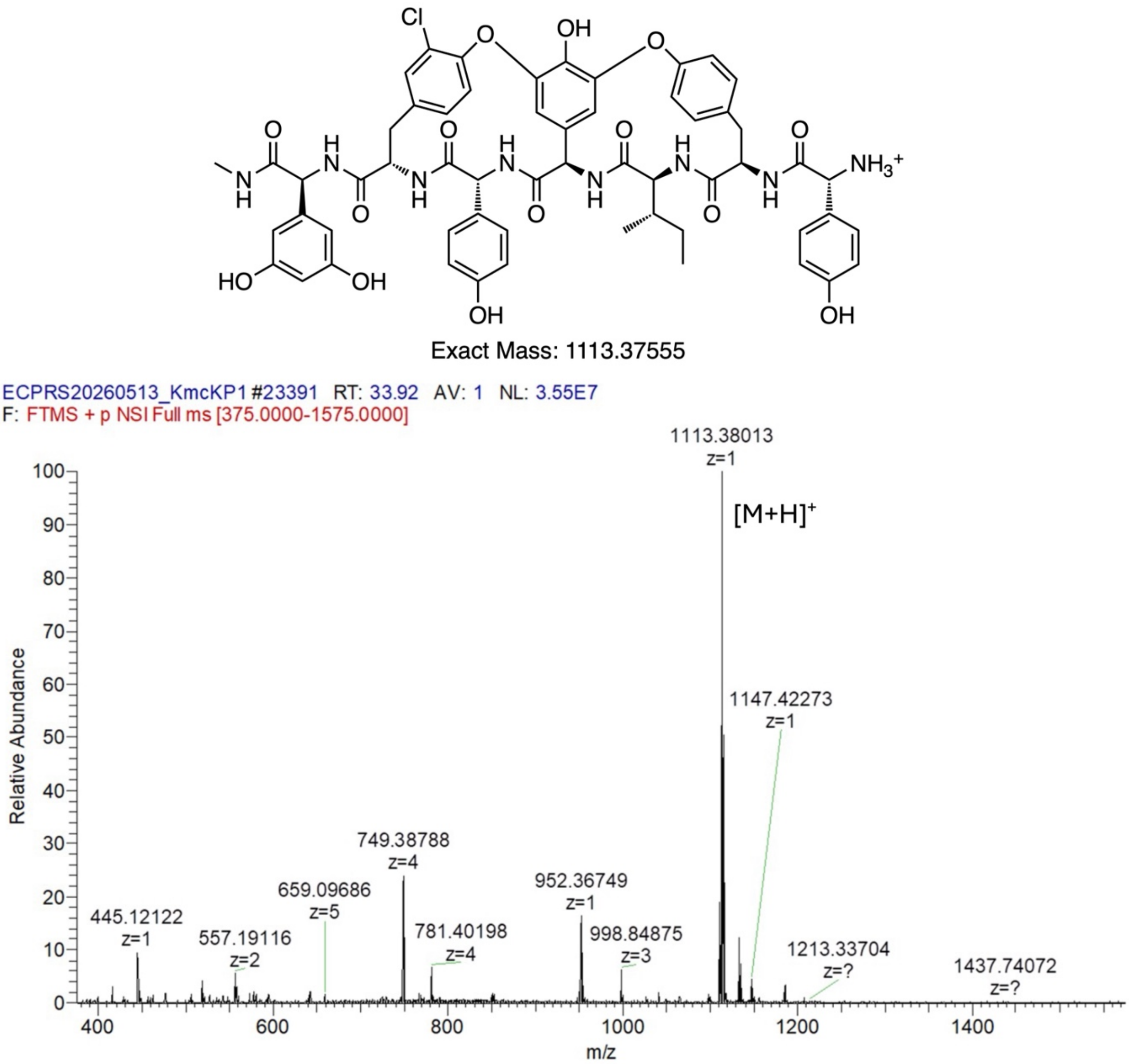

SI Figure 46. HRMS of the bicyclic methylamide product of **3** synthesised via Method C from crosslinking reaction with $OxyB_{bal}$, $OxyA_{ris}$ and $OxyC_{cep.}$

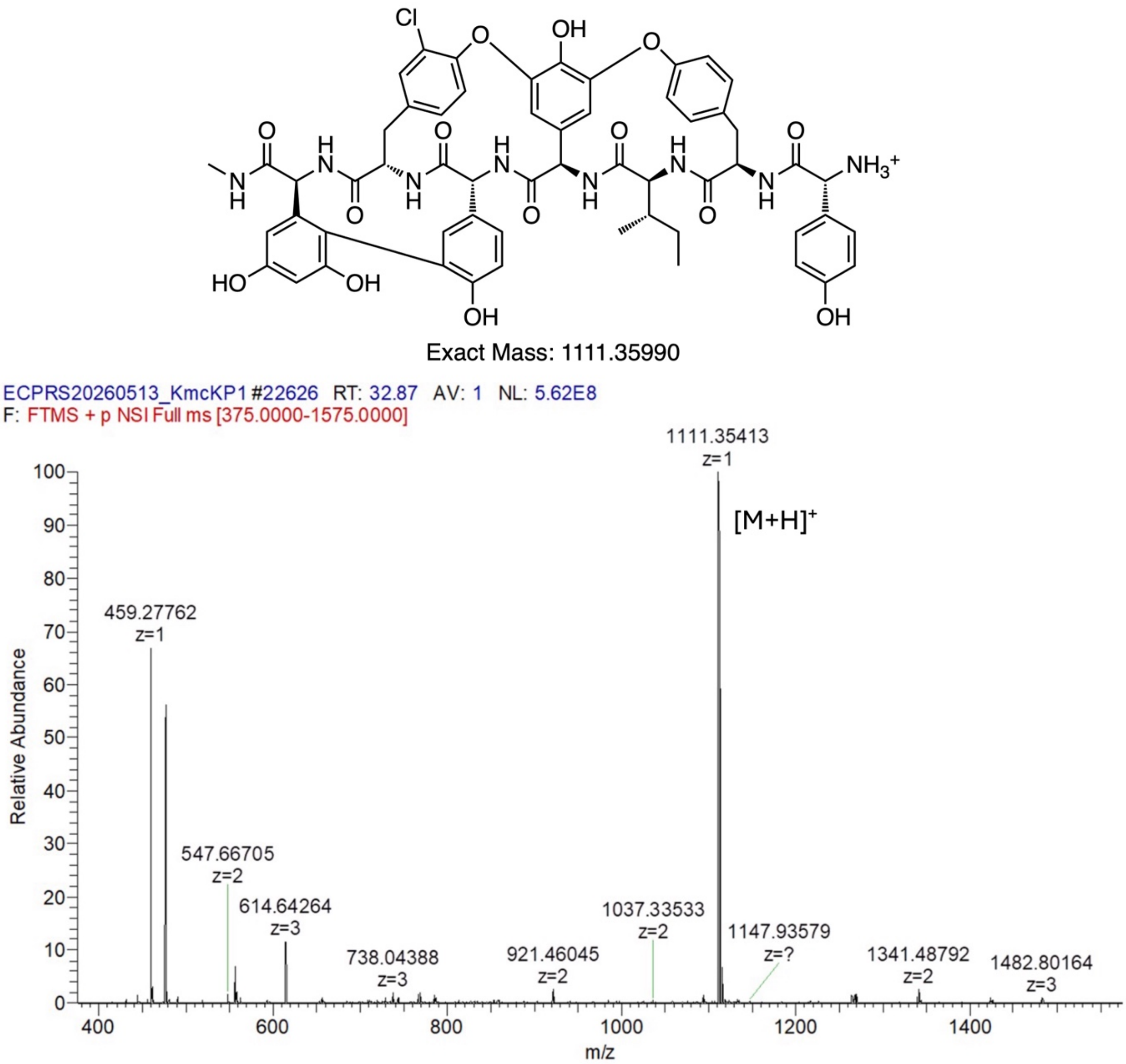

SI Figure 47. HRMS of the tricyclic methylamide product of **3** synthesised via Method C from crosslinking reaction with $OxyB_{bal}$, $OxyA_{ris}$ and $OxyC_{cep.}$

References

1. C. Brieke, M. J. Cryle, *Org. Lett.* **2014**, *16*, 9, 2454-2457.

2. R. Raz, F. Burlina, M. Ismail, J. Downward, J. Li, S. J. Smerdon, M. Quibell, P. D. White, J. Offer, *Angew. Chem. Int. Ed.* **2016**, 55, 13174.

3. J. Tailhades, M. Schoppet, A. Greule, M. Peschke, C. Brieke, M. J. Cryle, *Chem. Commun.* **2018**, *54*, 17, 2146–2149.

4. E. Marschall, R. W. Cass, K. M. Prasad, J. D. Swarbrick, A. I. McKay, J. A. E. Payne, M. J. Cryle, J. Tailhades, *Chem. Sci.* **2024**, *15*, 1, 195–203.

5. G.-M. Fang, Y. M. Li, F. Shen, Y. C. Huang, J. B. Li, Y. Lin, H. K. Cui, L. Liu, *Angew. Chem. Int. Ed.* **2011**, 50, 7645-7649.

6. D. T. Flood, J. C. J. Hintzen, M. J. Bird, P. A. Cistrone, J. S. Chen, P. E. Dawson, *Angew. Chem.* **2018**, 130, 11808.

7. M. Sunbul, N. J. Marshall, Y. Zou, K. Zhang and J. Yin, *J. Mol. Biol.*, **2009**, *387*, 883-898.

8. K. Haslinger, M. Peschke, C. Brieke, E. Maximowitsch and M. J. Cryle, *Nature*, **2015**, 521, 105-109.

9. M. Schoppet, J. Tailhades, K. Kulkarni and M. J. Cryle, *J. Org. Chem.*, **2018**, 83, 7206-7214.

10. S. G. Bell, F. Xu, E. O. D. Johnson, I. M. Forward, M. Bartlam, Z. Rao and L.-L. Wong, *J. Biol. Inorg. Chem.*, **2010**, *15*(3), 315–328.
